# Large gradual solar energetic particle events

**DOI:** 10.1007/s41116-016-0002-5

**Published:** 2016-09-07

**Authors:** Mihir Desai, Joe Giacalone

**Affiliations:** 1 Southwest Research Institute, 6220 Culebra Road, San Antonio, TX 78238 USA; 2 Department of Planetary Sciences, University of Arizona, Tucson, AZ 85721 USA

**Keywords:** Solar activity, Solar energetic particles, Coronal mass ejections, Shocks, Particle radiation, Space weather

## Abstract

Solar energetic particles, or SEPs, from suprathermal (few keV) up to relativistic ($\sim$few GeV) energies are accelerated near the Sun in at least two ways: (1) by magnetic reconnection-driven processes during solar flares resulting in impulsive SEPs, and (2) at fast coronal-mass-ejection-driven shock waves that produce large gradual SEP events. Large gradual SEP events are of particular interest because the accompanying high-energy (${>}10$s MeV) protons pose serious radiation threats to human explorers living and working beyond low-Earth orbit and to technological assets such as communications and scientific satellites in space. However, a complete understanding of these large SEP events has eluded us primarily because their properties, as observed in Earth orbit, are smeared due to mixing and contributions from many important physical effects. This paper provides a comprehensive review of the current state of knowledge of these important phenomena, and summarizes some of the key questions that will be addressed by two upcoming missions—NASA’s Solar Probe Plus and ESA’s Solar Orbiter. Both of these missions are designed to directly and repeatedly sample the near-Sun environments where interplanetary scattering and transport effects are significantly reduced, allowing us to discriminate between different acceleration sites and mechanisms and to isolate the contributions of numerous physical processes occurring during large SEP events.

## Introduction

### Historical perspective: pre-space age

**Fig. 1:**
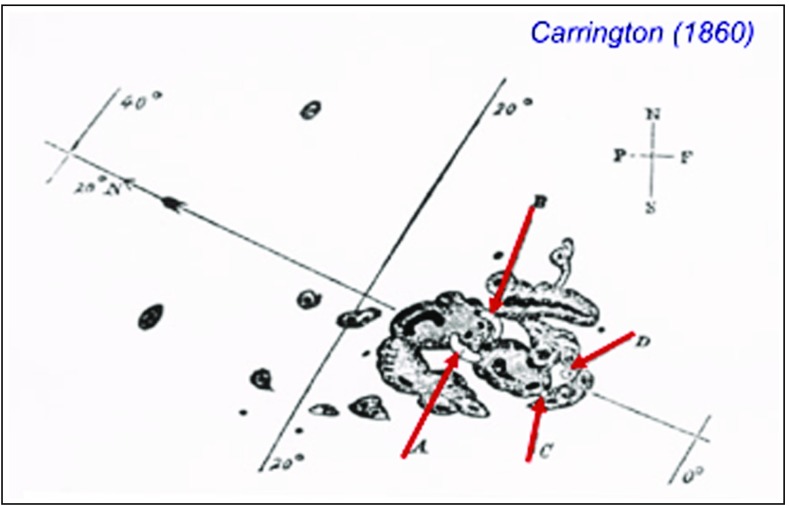
Carrington’s (1859) drawing of sunspot group 520 on September 1, 1859: the first visual record of a solar flare. The initial (*A*, *B*) and final (*C*, *D*) positions of the white-light emission are shown. Solar east is to the *right*

Motivated by the discovery of the sunspot cycle by Schwabe (1844) and an apparent connection between variations in sunspots and geomagnetic activity by Sabine (1852), Richard Carrington embarked on a comprehensive study of sunspots over an ${\sim }8$-year period from November 9, 1853 to March 24, 1861. Carrington’s discoveries included determination of the Sun’s rotation axis, the latitudinal variation of sunspots over a solar cycle, and the differential rotation of the Sun’s poles compared with the equatorial regions. An excellent account of Carrington’s scientific work and its impact on solar and space physics is provided in a review article by Cliver and Keer (2012). September 1, 1859, marks the first visual observation of a solar flare by Carrington (1859), and independently by Hodgson (1859). In an eloquent article, entitled “Description of a Singular Appearance seen in the Sun on September 1, 1859,” Carrington (1859) describes his observations of that day (quoted and paraphrased):

While engaged in the forenoon of Thursday, September 1, in taking his customary observation of the form and positions of the solar spots, an appearance was witnessed which he believed to be exceedingly rare. Describing it as the break out of two patches of intensely white light (identified as A and B in Figure 1), Carrington’s first impression was that by some chance a ray of light had penetrated a hole in the screen attached to the object-glass. After convincing himself that this outburst was real and noticing that it was increasing very rapidly, he ran to call someone else to witness the exhibition. Returning within 60 s, he was then mortified to find that it was already much changed and enfeebled. Shortly afterwards the last trace was gone, and although he maintained a strict watch for nearly an hour, no recurrence took place. He observed the last traces at C and D, the patches having traveled considerably from their first position and vanishing as two rapidly fading dots of white light. Carrington noted that the outburst lasted less than ${\sim }5$ minutes, from ${\sim }1118$ to ${\sim }1123$ Greenwich mean time (GMT).

Later, Carrington also noted that on September 1 at 1120 GMT, the three magnetic elements obtained at Kew Observatory exhibited moderate but very marked variations, and that a great magnetic storm had commenced around 0400 GMT on September 2. Subsequent accounts established that the storm’s effects were “as considerable in the southern as in the northern hemisphere.” Duly noting “the contemporary occurrence of solar activity and the geomagnetic disturbance,” Carrington still did not rush to connect them at that time. Having searched in vain for other instances of the simultaneity of solar eruptions and geomagnetic disturbances, many scientists, including Lord Kelvin (see Ellis 1901), abandoned the notion that an erupting sunspot may have a causal relationship with co-temporal geomagnetic activity. Some 80 years later, following the discovery that bright eruptions in the solar chromosphere cause simultaneous radio fade-outs and distinct terrestrial effects by Fleming (1936), Bartels (1937) provided a complete description of what is now universally known as the 1859 Carrington event.

Describing it as “one of the six outstanding storms observed in the last 100 years,” Bartels (1937) summarized the events of September 1–3, 1859:

The unusually large solar eruption observed by Carrington was accompanied by a simultaneous large magnetic effect lasting less than an hour, presumably caused primarily by a transitory increase of ionization in the ionosphere due to excessive ultra-violet light, and was followed after an interval of $17^\mathrm{h}\,35^\mathrm{m}$, by the outbreak of one of the six most violent storms ever observed, presumably caused primarily by the impact of solar corpuscles.

We now know that radio fade-outs occur when X-rays from solar flares arrive at Earth and increase the ionization of the ionospheric D layer, which in turn results in the absorption of radio communication signals. We have also realized that large flares are typically accompanied by violent expulsions of fast coronal mass ejections (CMEs) into the heliosphere and that, if these CMEs are faster than the speed of the ambient solar wind (SW) ahead, they drive strong interplanetary (IP) shock waves. Violent geomagnetic storms may occur when the IP shock and its driver CME arrive at Earth. It is widely accepted that protons, electrons, and heavier nuclei such as He–Fe are accelerated from a $\sim$few keV up to GeV energies in at least two distinct locations, namely, the solar flare and the CME-driven IP shock. The particles observed in interplanetary space and near Earth are commonly referred to as solar energetic particles or SEPs: those accelerated at flares are known as impulsive SEP events, particle populations accelerated by near-Sun CME-shocks are termed as gradual SEPs, and those associated with CME shocks observed near Earth are known as energetic storm particles or ESP events.

**Fig. 2:**
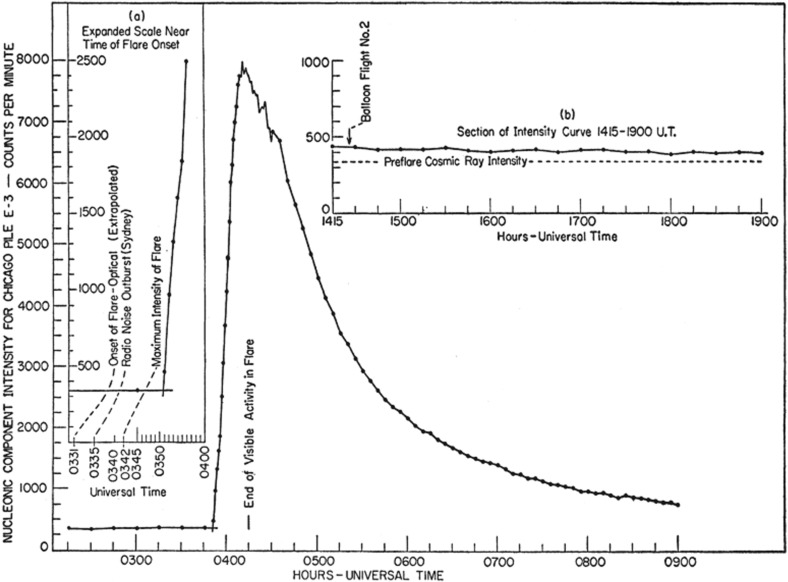
Neutron monitor observations during the 1956 solar flare event. Image reproduced with permission from Meyer et al. (1956), copyright by APS

### Space era: a paradigm shift and the two-class picture

The earliest observations of SEP events extending up to GeV energies were made with ground-based ionization chambers and neutron monitors (Forbush 1946; Meyer et al. 1956). Since such events, also known as ground level enhancements or GLEs (see Fig. 2), were closely associated with H$\alpha$ flares on the Sun, it was presumed that there was a causal relationship between the flare and the energetic particles observed at 1 AU. These and subsequent observations sowed the seeds for a popular scenario—the so-called “solar flare myth” (see Gosling 1993)—that persisted well into the 1990s. In this scenario, large solar flares are the primary cause of large, non-recurrent geomagnetic storms, transient shock wave disturbances in the SW, and major energetic particle events seen in interplanetary space.

Even so, on the basis of a close association between the SEP events and slow-drifting type II and various kinds of type IV radio bursts, Wild et al. (1963) proposed that the energetic particles might be accelerated at magnetohydrodynamic shock waves that typically accompanied the flares. Later, Lin (1970) reported close associations between ‘pure’ electron events and flares that only exhibited metric type III emissions on the one hand, and ‘mixed’ events with protons and relativistic electrons and flares with type II/IV radio events on the other hand, proposing a ‘two-phase’ acceleration process for the SEP events observed in space.

Despite these results, a two-class paradigm for SEP events was not generally accepted until the mid-1990s. The close association between CMEs observed on Skylab and large solar proton events led Kahler et al. (1978) to suggest an important role for the CME either in creating open field lines for flare particles to escape into the interplanetary medium or for the protons to be accelerated near a region above or around the outward moving ejecta far above the flare site. Subsequently, detailed analyses of flare durations, longitudinal distributions from multi-spacecraft observations, high resolution ionic charge state and elemental composition measurements, and clearer associations with radio bursts led most researchers to accept the view that the SEP events observed at 1 AU belong to two distinct classes, impulsive and gradual (e.g., Kahler et al. 1978, 1984; Cliver et al. 1982; Kocharov 1983; Luhn et al. 1984; Mason et al. 1984; Cane et al. 1986; Reames 1988a). We now know that the arrival of “solar corpuscles” as discussed by Bartels (1937) heralds the arrival of fast coronal mass ejections or CMEs (see Gosling 1993).

**Fig. 3:**
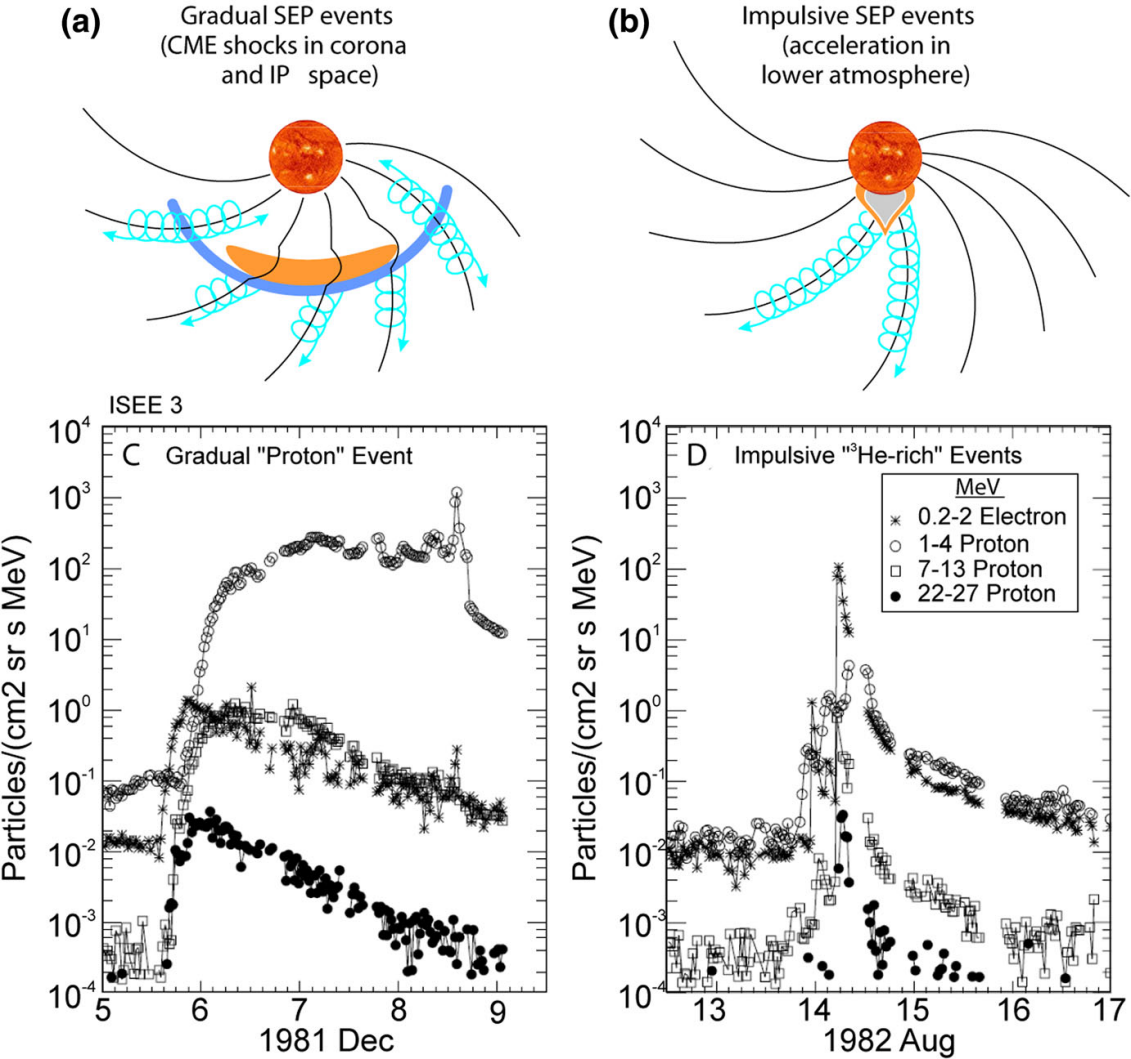
The two-class picture for SEP events where **a** the gradual event is produced by a large-scale CME-driven shock wave that accelerates the SEPs and populates interplanetary magnetic field (IMF) *lines* over a large longitudinal area, and **b** the impulsive event is produced by a solar flare that populates only those IMF lines well-connected to the flare site. Intensity-time profiles of electrons and protons in **c** a large gradual SEP event, and **d** a small impulsive SEP event (adapted from Reames 1999)

By the end of the 1990s, a two-class picture (see Fig. 3; Table 1) for SEP events had emerged. Here the gradual events occurred as a result of diffusive acceleration at CME-driven coronal and interplanetary (IP) shocks, while the impulsive events were attributed to acceleration during magnetic reconnection in solar flares (e.g., Reames 1999). The gradual or CME-related events typically lasted several days and had larger fluences, while the impulsive or flare-related events lasted a few hours and had smaller fluences. Impulsive events were typically observed when the observer was magnetically connected to the flare site, while ions accelerated at the expanding large-scale CME-driven shocks can populate magnetic field lines over a significantly broad range of longitudes (Cane et al. 1988). The distinction between impulsive and gradual SEP events was further justified on the basis of the energetic particle composition and radio observations (e.g., Cane et al. 1986). For instance, the flare-related impulsive SEP events were electron-rich and associated with type III radio bursts. These events also had $^{3}\hbox {He}/^{4}$He ratios enhanced between factors of $10^3$–$10^4$, Fe/O ratios enhanced by up to a factor of 10 over the corresponding SW values, and had Fe with ionization states up to $\sim$20. In contrast, the gradual events were proton-rich, had average Fe/O ratios of $\sim$0.1 with Fe ionization states of $\sim$14, had no measurable enhancements in the $^{3}\hbox {He}/^{4}$He ratio, and were associated with type II bursts (e.g., Reames 1999; Cliver 2000). It is now believed that CME-driven coronal and interplanetary shocks are the most prolific producers of SEPs that pose radiation hazards for us, our environment, and our assets on Earth and in space (Reames 1999).

**Table 1:** Two-class paradigm of SEPs (from Reames 1995b; Kallenrode 2003)

Property	Impulsive	Gradual
Electron/proton	${\sim }10^{2}$–$10^{4}$	${\sim }50$–100
$^{3}$He/$^{4}$He	${\sim }1$	${\sim }4\times 10^{-4}$
Fe/O	${\sim }1$	${\sim }0.1$
H/He	${\sim }10$	${\sim }100$
Q$_{\mathrm{Fe}}$	${\sim }20$	${\sim }14$
SEP duration	${<}1$–20 h	${<}1$–3 days
Longitude cone	${<}30^{\circ }$	${<}100^{\circ }$–$200^{\circ }$
Seed particles	Heated Corona	Ambient Corona or SW
Radio type	III	II
X-ray duration	${\sim }10$ min–1 h	${\gtrsim }1$ h
Coronagraph	N/A	CME
Solar eind	N/A	IP shock
Events/year	${\sim }1000$	${\sim }10$

This review attempts to provide a comprehensive picture of the observations and theoretical concepts relevant to large gradual SEP events. A subsequent review will discuss the $^{3}$He-rich or impulsive SEP events. We start in Sect. 2 by describing state-of-the-art observations that focus on the origin, acceleration, and transport of remotely accelerated large SEP events. In Sect. 3, we describe observations of the locally measured CME-shock accelerated particle populations known as ESP events. In Sect. 4, we discuss the extremely large SEP events, known as GLEs, that create signatures in ground-based cosmic ray neutron monitors. In Sect. 5, we review observational and theoretical ideas concerning the origin and acceleration of the poorly measured and understood suprathermal population, which serves as a source of material for CME-driven shocks. Section 6 presents the current status of multi-spacecraft, longitudinally separated SEP observations that have challenged existing notions about source sizes and locations, as well as ways in which particles are transported in the inner heliosphere. Section 7 provides a summary of the theoretical concepts that are relevant to SEP acceleration and transport. In Sect. 8, we discuss the future outlook for SEP studies, particularly how measurements from new inner heliospheric missions such as Solar Probe Plus (SPP) and Solar Orbiter (SolO) during the next decade (2017–2027) will revolutionize and overturn many of our existing notions about the relationships between CMEs, shocks, seed populations, turbulence and waves, and large SEP events. Finally, we conclude this review by emphasizing the fact that, in order to maximize the return of these new missions, we also need to make critical near-Earth in-situ measurements that serve as the ground-truth for SEP acceleration and transport models. Satellites near Earth orbit are critical for measuring the convolved and combined end effects of multiple physical processes that contribute to SEP events.

## Large gradual solar energetic particle events

As discussed above and in Sect. 3, an ESP event is observed when an IP shock arrives at a given location; at ${\sim }1\,\mathrm{AU}$ this is typically $\sim$2–4 days after the driver CME leaves the Sun. Somewhat earlier in its lifecycle, however, the near-Sun CME shock is likely to be substantially faster and therefore should drive a stronger shock that is far more efficient at accelerating particles than its near-Earth counterpart (e.g., Kallenrode et al. 1993; Rice et al. 2003). The ion populations accelerated by near-Sun CME shocks arrive significantly earlier compared with the IP shocks and their associated ESP events, and are known as large gradual SEP events. However, since CMEs and solar flares are nearly co-temporal and occur when the same or nearby active regions erupt, the precise origin of the remotely accelerated SEPs continues to be hotly debated (see Sects. 2.6.1, 2.6.3 for the opposing viewpoints of Cane et al. 2006; Tylka et al. 2005). This situation is exacerbated by the fact that properties of large gradual SEPs are influenced by a confluence of multiple processes and effects; by the time they are observed at 1 AU, scattering during transport plays an important role. Other important factors include: (1) origin and variability of the suprathermal seed populations; (2) the efficiency with which populations from different sources and with distinct distribution functions are injected into the shock acceleration mechanisms; (3) factors that control the efficiency with which particles are accelerated (e.g., CME speed, kinetic energy); (4) the presence or absence of multiple, interacting CMEs; (5) the type, level, and characteristics of the waves and turbulence present near the shock and in the interplanetary medium; and (6) the charge-to-mass (Q/M)-dependence of scattering and transport through the turbulent interplanetary medium. This section summarizes the observational evidence that points to the importance of these factors in large SEP events.

### Early multi-spacecraft observations

**Fig. 4:**
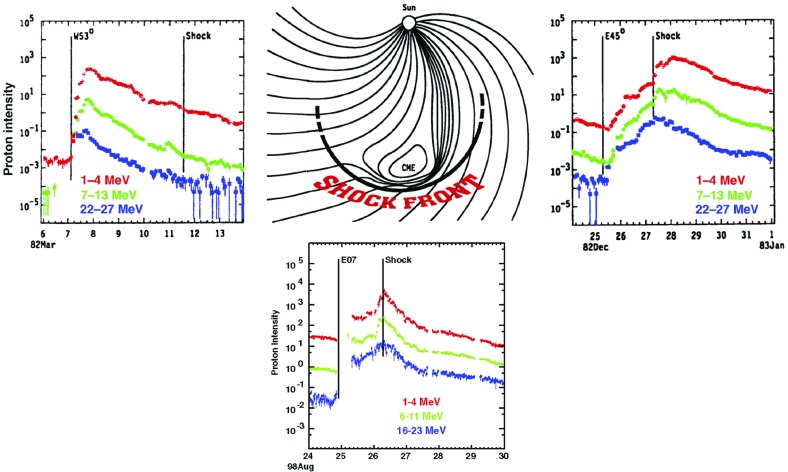
Intensity-time profiles of $\sim$1–30 MeV protons during gradual SEP events observed at three different solar longitudes relative to the flare or CME lift-off location (see text; after Cane et al. 1988; Reames 1999; Cane and Lario 2006)

Approximately 20 years of multi-spacecraft SEP observations show that the time-intensity histories of $\sim$1–30 MeV protons in large gradual SEP events can be understood if the strongest acceleration occurs near the “nose” of a CME shock that moves radially outward from the Sun (see Fig. 4; Cane et al. 1988; Reames 1995b; Reames et al. 1997). For spacecraft (s/c) located east of the source (left panel), the intensities show abrupt increases and peak relatively earlier during the event when it is magnetically connected to the nose of the CME shock near the Sun. The intensities decay slowly as the shock moves outward and the s/c becomes magnetically connected to the eastern flanks of the shock. In contrast, for sources located near the central meridian, the intensities peak when the nose of the shock reaches the s/c location. Spacecraft located to the west (right panel) of the source observe a slow increase in the intensities that peak well after the shock is observed locally. Based on the distinct time histories shown in Fig. 4, Cane et al. (1988) established the role of CME-driven shocks in large SEP events.

**Fig. 5:**
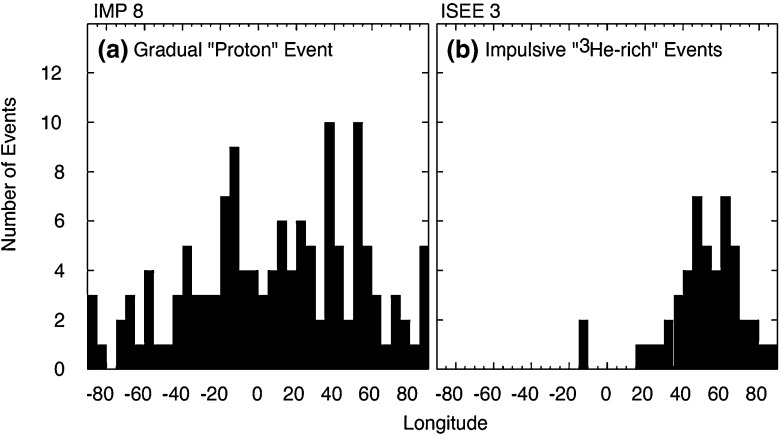
Longitudinal distributions of the solar sources associated with **a** gradual and **b** impulsive SEP events. Image reproduced with permission from Reames (1999), copyright by Springer

Figure 5 shows the longitude distribution of the associated flare for several gradual and impulsive events. Gradual events are observed regardless of the relative location (east-or-west) of the flare longitude, while impulsive events are observed primarily when the observer is magnetically well-connected to the flare site on the western hemisphere. This comparison shows that the broad longitudinal distribution of gradual events is unlikely to occur as a result of rapid coronal diffusion or cross-field transport, because such effects should also occur during the smaller impulsive SEP events. Rather, the observed longitudinal spread of gradual SEPs provides further support for the notion that a CME shock that accelerates particles across its surface can easily populate a broad swath of of interplanetary magnetic field (IMF) lines as it moves further out into the heliosphere. The more recent, multi-spacecraft observations of large SEP events and their implications are discussed further in Sect. 6.6.

### Evidence for CME shocks in the solar corona

**Fig. 6:**
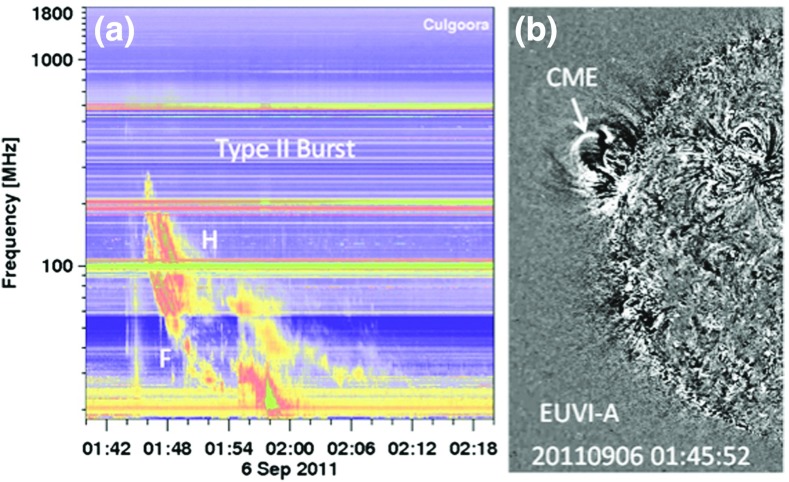
**a** Dynamic spectrum from the Culgoora radio observatory showing a type II burst with fundamental (F) and harmonic (H) structure. The fundamental component starts around 150 MHz. **b** A section of the nearest STEREO A EUVI-A image showing the CME. The CME height can be directly measured from this frame as $1.29\,R_{S}$. Image reproduced with permission from Gopalswamy et al. (2013), copyright by COSPAR

An important source of information about the formation and properties of CME-driven shocks in the solar corona at distances below ${\sim }10\,R_{S}$ comes from observations of the so-called type II solar radio bursts. These bursts appear as slowly drifting pairs of band-like features in the dynamic spectra (frequency vs. time, with color-coded intensity; see Fig. 6). The pairs differ in frequency by a factor of ${\sim }2$ and are attributed to CME-shock accelerated electrons that drive Langmuir waves near the electron plasma frequency, $f_{p}$, and produce radio emission near $f_{p}$ and $2f_{p}$ (e.g., Wild et al. 1963; Cairns et al. 2003; Gopalswamy et al. 2013). Interplanetary type II bursts with similar pairs of band-like features are also found in association with transient CME-driven shock waves (e.g., Cane et al. 1982; Reiner et al. 1998; Bale et al. 1999). Recently, detailed magnetohydrodynamic (MHD) simulations of CME initiation and propagation combined with multi-point measurements of type II radio bursts, extreme ultraviolet (EUV) spectroscopy, and white-light coronagraph images have greatly expanded our understanding of CME shock formation in the low solar corona below ${\sim }5\,R_{S}$ (e.g., Schmidt et al. 2013). It is now accepted that CME shock formation can occur at heights substantially below ${\sim }1.5\,R_{S}$ (e.g., Gopalswamy et al. 2013), which is critical for understanding the physics of particle acceleration, e.g., the release times of SEPs during GLEs (Reames 2009a).

### SEPs and CME properties

**Fig. 7:**
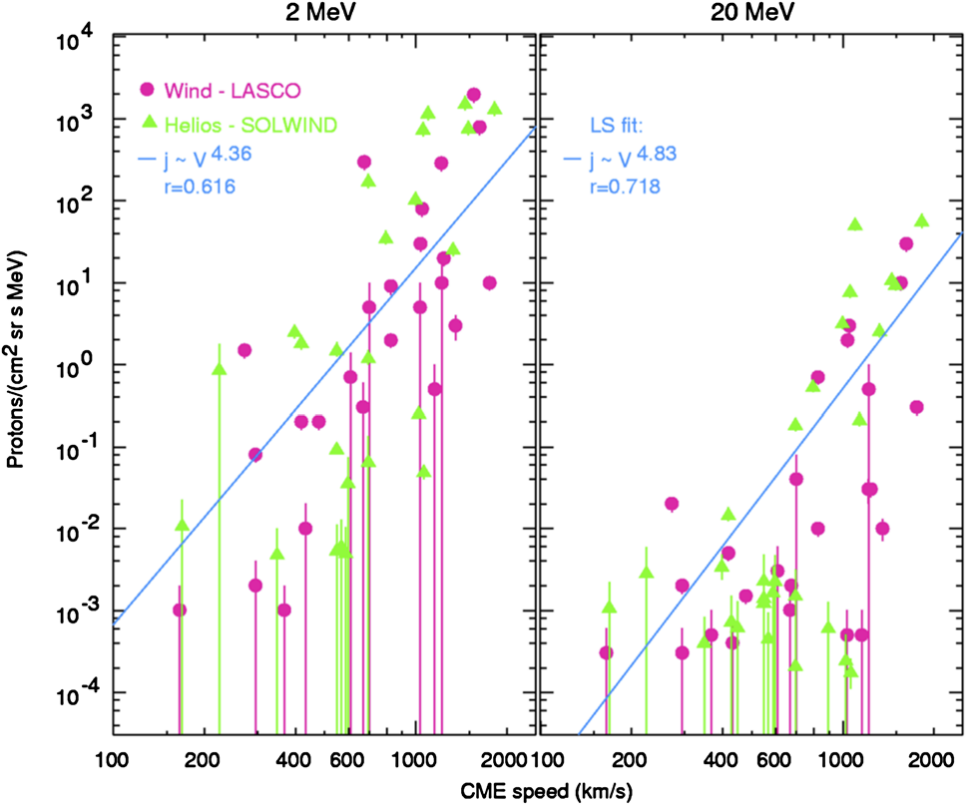
Peak proton intensity in SEP events at two energies versus CME speed. *Pink circles* represent data from wind/energetic particles—acceleration, composition, and transport/low energy matrix telescope (EPACT/LEMT) and SoHO/LASCO; *green triangles* show data from *Helios* and Solwind, P78-1; *blue lines* are linear least-squares fits, *r* are the corresponding correlation coefficients. Image reproduced with permission from Kahler (2001), copyright by AGU

Comparisons between CME or IP shock and SEP properties have revealed significant scatter from clear correlations. Depending on the ambient SW speed ahead, faster CME drivers are generally thought to drive stronger shocks (e.g., Rice et al. 2003), and may therefore be important for particle acceleration. However, Fig. 7 shows that CMEs with similar speeds are associated with huge variations (${\sim }3$–4 orders of magnitude) in the intensities of the associated SEPs at 1 AU (Kahler 2001), posing real challenges in our ability to model and predict SEP properties based on known CME properties. Likewise, Sect. 3 shows that the lack of clear relationships between various properties (e.g., peak intensities, spectral indices, etc.) of ESP events and the locally measured IP shock parameters (e.g., compression ratio) indicates that many factors can contribute to the local diffusive shock acceleration processes and cause the event-to-event variability.

Emslie et al. (2012) found that only 22 of the 38 largest solar eruptive events are associated with large SEP events, while Gopalswamy et al. (2008) found that some of the most energetic CMEs are not associated either with type II radio bursts or large SEPs. More recently, Kahler (2013a) calculated three different SEP event timescales: (a) the time from inferred CME launch at $1\,R_{S}$ to the time of the 20 MeV SEP onset at Wind, (b) the time from SEP onset to the time the intensity reached half the peak value, and (c) the time during which the intensity remained above half the peak value. These three timescales ranged between about an order of magnitude and were then compared with CME properties such as speed, acceleration, width, and location. The main results of this survey are that the onset time (a), decreased with CME speed and width, while the timescales that characterized the peak intensity, i.e., (b) and (c), increased with CME width and speed. These results confirm that faster (and wider) CMEs drive shocks and accelerate SEPs over longer times to produce events with longer timescales and larger fluences.

**Fig. 8:**
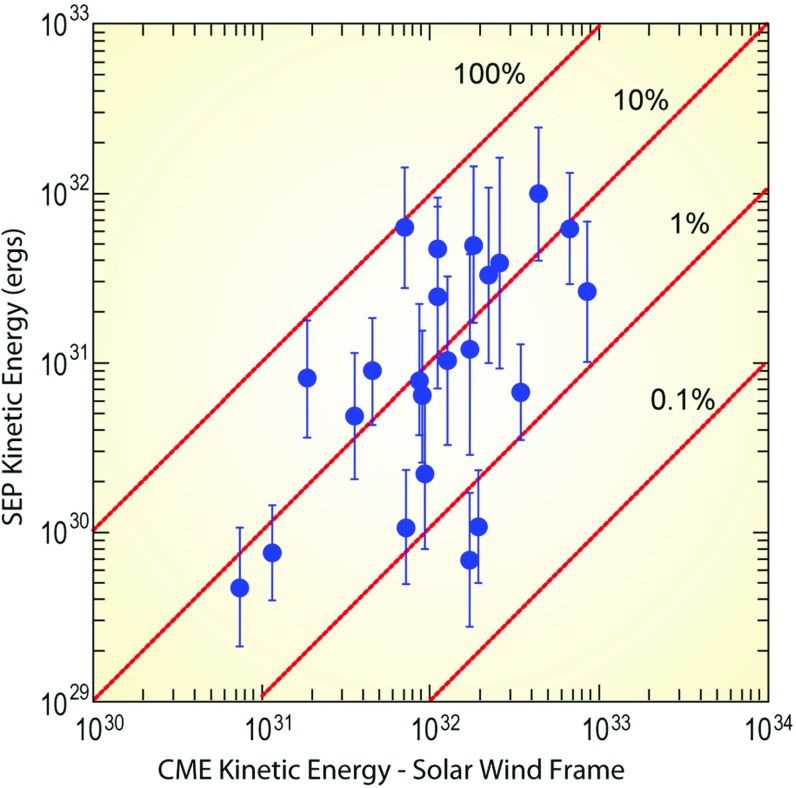
Scatter-plot of CME kinetic energy versus SEP kinetic energy for 23 large SEP events from solar cycle 23. Image adapted from Mewaldt et al. (2008)

Other studies have estimated that CMEs associated with large SEP events can expend different fractions of their total kinetic energy into accelerating SEPs (see Fig. 8). Mewaldt et al. (2008) estimated SEP kinetic energies during 23 of the largest SEP events of cycle 23 using the fluence spectra measured by instruments on advanced composition explorer (ACE), solar, anomalous, and magnetospheric particle explorer (SAMPEX) and geostationary operational environmental satellites (GOES) from ${\sim }0.03$ to ${\sim }500\,\mathrm{MeV/nucleon}$. These estimates take into account the source locations and the longitude distribution of large SEPs, the possibility that SEPs can cross Earth-orbit multiple times, and transport effects such as adiabatic deceleration and pitch-angle scattering. The kinetic energies of the associated CMEs were measured by the Large Angle and Spectrometric Coronagraph experiment (LASCO) on Solar and Heliospheric Observatory (SoHO) (see, e.g., Ontiveros and Vourlidas 2009). Using these parameters and the measured proton spectra, and integrating over energy, time, and space, Mewaldt et al. (2008) and Emslie et al. (2012) compared the CME and SEP kinetic energies in the rest frame of the SW (e.g., see Fig. 8), and found that CMEs with energies of ${\sim }10^{32}\,\mathrm{ergs}$ could use between ${<}0.4$ and ${\sim }20~\%$ of their energies in accelerating SEPs. These authors also found that, on average, CMEs use ${\sim }5$–$10\,\%$ of their kinetic energy into accelerating SEPs. Similar estimates are obtained for supernovae shocks that accelerate galactic cosmic rays that fill the galaxy (e.g., Ptuskin 2001). Finally, Mewaldt et al. (2005b, (2008) also found that the so-called GLE events were associated with ${\sim }30\,\%$ of the very energetic CMEs with kinetic energies ${\gtrsim }1.2\times 10^{32}\,\mathrm{ergs}$ (also see Gopalswamy 2006).

**Fig. 9:**
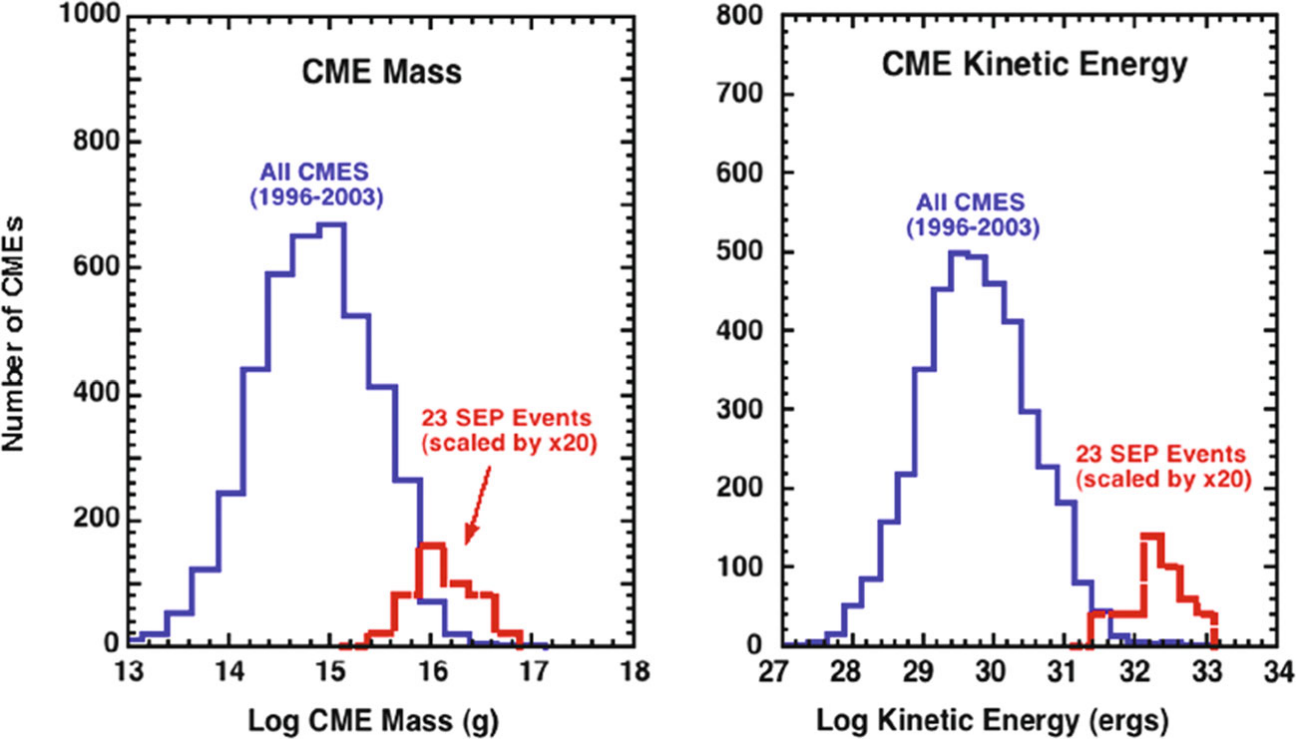
*Left* Comparison of the mass distribution of all CMEs observed from 1996–2003 (Gopalswamy 2006) to the masses of CMEs associated with 23 of the 50 largest SEP events of solar cycle 23 (scaled up by 20). *Right* Comparison between the distributions of the kinetic energy of CMEs associated with 23 large SEP events from solar cycle 23 and all CMEs observed from 1996–2003. Images reproduced with permission from Mewaldt et al. (2008), copyright by AIP

The largest SEP events are associated with the fastest ${\sim }1$–$2\,\%$ of CMEs. The CMEs have typical speeds ${>}1500\,\mathrm{km/s}$, although a few have speeds as low as ${\sim }700$–$800\,\mathrm{km/s}$ (Kahler 2001). Figure 9 compares the mass (left) and energy (right) distributions of all CMEs (in blue) with those associated with 23 of the 50 largest SEP events (in red) from solar cycle 23. Similarly, Yurchyshyn et al. (2005) found that the distributions of the plane-of-sky-speeds for ${>}$4000 CMEs, whether they are accelerating or decelerating, showed no physical distinction and exhibited log-normal forms similar to the ones shown in Fig. 9. The figure clearly shows that large SEP events are associated with CMEs that have masses ${>}10^{15}\,\mathrm{g}$ and kinetic energies ${>}3\times 10^{31}\,\mathrm{ergs}$, with the kinetic energy of the CME being more indicative of whether the associated SEP event is also likely to be large and intense.

### Size distribution of SEP events

The size distribution of SEP events has often been characterized in terms of a power-law in the peak proton flux or fluence and then compared to the peak soft X-ray (SXR) flux in flares (see, e.g., Hudson 1978; Belov et al. 2007; Cliver et al. 2012, and references therein). However, the power-law characterizing SEP size is significantly flatter than that of the SXR flux. This is not surprising, given that large SEP events are believed to be produced by CME shock acceleration rather than by the associated flare (Reames 1999). Furthermore, Cliver et al. (2012) showed that the steeper SXR flux distribution occurs because there exist two other types of X-ray flares that are not associated with large SEP events: (1) those associated with the smaller $^{3}$He-rich SEP events (e.g., Mason et al. 2004), and (2) compact flares not associated with any escaping interplanetary SEP component. In fact, Cliver et al. (2012) showed that the difference in the slopes of the power-law size distributions of solar flares and SEP events arises primarily because the flares associated with large gradual SEPs represent an energetic subset of all flares that are also accompanied by fast (${>}1000\,\mathrm{km/s}$) CMEs. They also showed that the small difference of ${\sim }0.15$ between the slopes of the distributions of SEP events and the peak SXR fluxes during the associated flares is consistent with the observed variation of SEP event peak flux with SXR peak flux. Finally, using several lines of evidence, Kahler (2013b) argued against using scaling laws to describe the relationship between the SEP event peak fluxes and SXR peak fluxes, and therefore, against a close physical connection between flares and SEP production. They instead suggest that the differences in the power-law distributions of the SXR peak fluxes and that of the SEP peak fluxes can be understood in terms of the fractal-diffusive self-organized criticality model proposed by Aschwanden (2012), which decouples the causal and physical connections between flares and large gradual SEPs events.

### SEPs associated with interacting or twin-CMEs

**Fig. 10:**
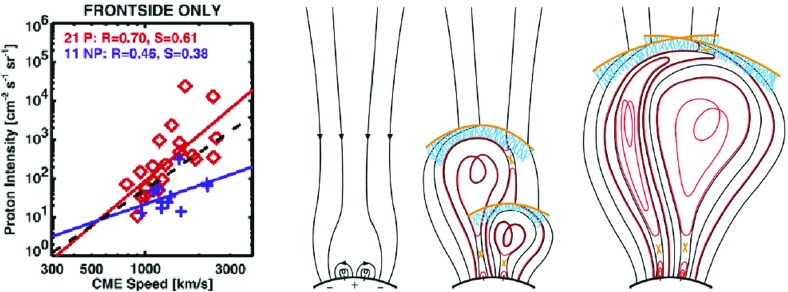
*Left* Peak proton intensity versus CME speed for SEP events with a preceding frontside CME (P; *red diamonds*) and for no preceding CME (NP; *plus symbols*). *Solid lines are regression lines* for the P and NP groups. The *dashed regression line* is for all data points. *Right* The “twin-CME” scenario for a large SEP event. Two CMEs erupt from the same or nearby source active regions. Interchange reconnection between open magnetic field lines and those draping the first CME can release seed particles accelerated by the first CME shock into the disturbed downstream region, which has enhanced turbulence levels. This material can then be subsequently accelerated by the second CME shock. Images reproduced with permission from (*left*) Gopalswamy et al. (2004), copyright by AGU, and (*right*) Li et al. (2012), copyright by Springer

Timing and correlation studies of cycle 23 SEP events show that (see Fig. 10, left) fast and wide CMEs erupting from an active region that also produced fast (${\sim }488\,\mathrm{km/s}$) and wide (${\ge }60^\circ$) CMEs within the preceding ${\sim }24$-h interval are almost always associated with large SEP events. Gopalswamy et al. (2004) suggest that the preceding CMEs may provide seed particles for CME-driven shocks that follow, and that this is the primary reason why SEP intensities in events without preceding CMEs are lower; in other words, the differences in SEP properties may not have resulted due to inherent properties of the CMEs themselves. The Li et al. (2012) survey of 16 GLEs in solar cycle 23 showed that fast and wide CMEs from the same active region are associated with GLEs, even if the preceding CMEs were slower (${>}300\,\mathrm{km/s}$) and narrower, and occurred within ${\sim }9$-h intervals (see Sect. 4; Li et al. 2012).

Some of the physical mechanisms that could account for these observations are: (1) the first CME shock disturbs the ambient coronal and interplanetary environment and enhances turbulence levels, which increase the efficiency of the second CME shock (e.g., Li and Zank 2005; Ding et al. 2013; 2) the first CME shock produces a suprathermal-through-energetic particle population whose intensities decay slowly with e-folding times of ${\sim }8$–$16\,\mathrm{h}$, thereby creating a pre-accelerated particle population that the second CME shock can readily inject and re-accelerate (e.g., Gopalswamy et al. 2004; Reames et al. 1997, 2013; Mewaldt et al. 2012a; 3) a pseudo-streamer-like pre-eruption magnetic field configuration leads to reconnection between closed field lines that drape the first CME and its shock as well as the open field lines that drape the second CME, creating enhanced seed populations and higher turbulence levels in front of the second CME shock (see Fig. 10, right); and (4) differences in open and closed field-line geometry and a decrease in Alfvén velocity creates a stronger shock in front of the second CME (Gopalswamy et al. 2004).

In contrast with the above studies, Kahler and Vourlidas (2014) argue against the interacting or twin-CME scenario as a direct cause of enhanced SEP intensities because they did not find any pre-CME property (e.g., number of CMEs, timing, widths, speeds) that correlated either with enhanced SEP proton intensities above ${\sim }20\,\mathrm{MeV}$ or with SEP event timescales. These results provided no clue as to how the preceding CMEs could interact with the primary CMEs and produce larger SEP events. Instead, they found that the SEP event intensities and the occurrence rates of pre-CMEs increases with the pre-event 2 MeV proton intensities. They suggested an alternate explanation for the association between pre-CMEs and enhanced SEP proton intensities: the 2 MeV pre-event particles serve as seed populations for the higher (${\sim }20\,\mathrm{MeV}$) energy SEPs, and that both, the CME occurrence rates and the increases in the pre-event 2 MeV SEP intensities are manifestations of higher solar activity. Re-analyzing the data from Gopalswamy et al. (2004) and Ding et al. (2013), Kahler and Vourlidas (2014) also found no correlation between enhanced SEP intensities and the ${\sim }2\,\mathrm{MeV}$ intensities measured during a significantly shorter (${\sim }2$ h) interval prior to the onset of the primary CME; the previous studies used 1-day intervals to measure the pre-event ${\sim }1\,\mathrm{MeV}$ intensity. On this basis, Kahler and Vourlidas (2014) ruled out the contributions of providing enhanced seed populations by the preceding CMEs. These results clearly imply that the origin of the enhancements in the pre-event seed population intensities remains unclear, and that the relationship between enhanced proton intensities in larger SEP events and CME interactions is still not well understood.

### Spectral variability

**Fig. 11:**
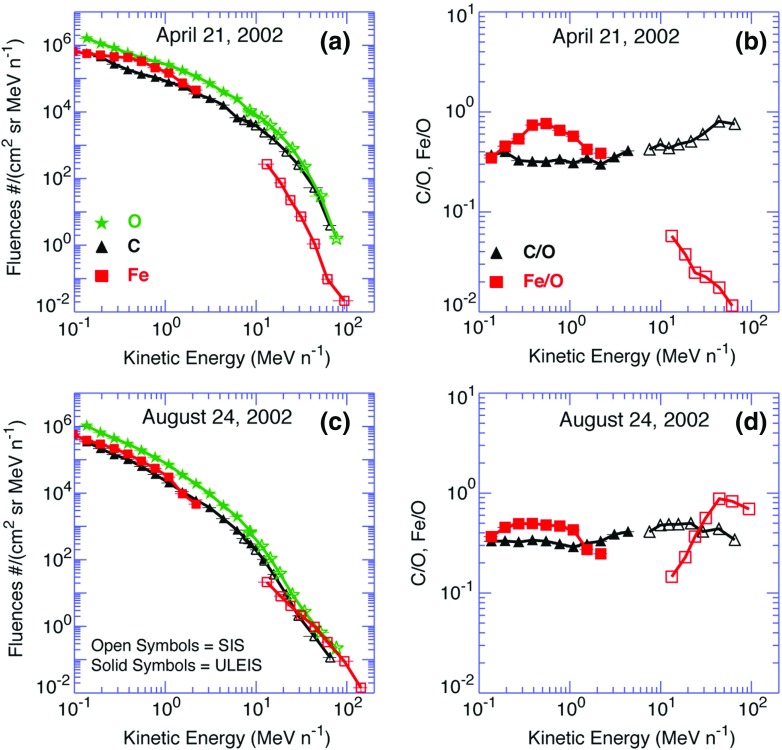
*Left* Event-integrated fluence spectra of C, O, and Fe. *Right* C/O and Fe/O ratios in two large SEP events measured by the Ultra Low Energy Isotope Spectrometer (ULEIS) and the Solar Isotope Spectrometer (SIS) on board ACE (adapted from, Tylka et al. 2005; Desai et al. 2006a)

One of the most puzzling aspects of large SEP observations of cycle 23 is the variability in the energy-dependent behavior of the Fe/O ratio between 0.1 and 100 MeV/nucleon. Figure 11 provides an example of such variability, as seen in the August 24, 2002 SEP event and the April 21, 2002 SEP events observed at ACE. Both events were associated with western hemisphere flares near $\sim$W80 and CMEs with similar speeds of ${\sim }2000\,\mathrm{km/s}$ (e.g., Cohen et al. 2003; Tylka et al. 2005), yet the associated heavy ion spectral behaviors were remarkably different. Diffusive shock acceleration (DSA) processes tend to accelerate ions with higher mass-per-charge (M/Q) ratios less efficiently than those with lower M/Q ratios (e.g., Desai et al. 2003). Since, Fe has higher M/Q than O, and since the abundances are normally measured in energy/nucleon rather than rigidity, the Fe/O ratio at equal energy/nucleon in large CME-shock accelerated SEP events is expected to decrease with increasing energy. Particle rigidity is defined as momentum per unit charge. Also, since C and O have similar M/Q ratios, the DSA processes are not expected to significantly alter the SEP C/O ratio with increasing energy. Thus, the nearly energy-independent C/O ratio observed in both SEP events in Fig. 11 is generally consistent with DSA of species with similar M/Q ratios. Likewise, the decrease in the Fe/O ratio during the April 21, 2002 event with increasing energy is also qualitatively consistent with shock acceleration models wherein Fe with higher M/Q ratio is accelerated less efficiently than O. However, the Fe/O ratio in many large SEP events of cycle 23, as seen during the August 24, 2002 SEP event, increased with increasing energy (e.g., Tylka et al. 2005; Cane et al. 2006), which is inconsistent with M/Q-dependent processes and poses serious challenge to DSA models.

The differences in the energy-dependent behavior of Fe/O could not be attributed to observed differences in the sources and their locations relative to ACE. Three plausible ideas could account for the increase in the Fe/O at higher energies: (1) direct flare contribution above ${\sim }10\,\mathrm{MeV/nucleon}$ (e.g., Cane et al. 2003, 2006), (2) re-acceleration of suprathermal and energetic particles from previous or accompanying flares (e.g., Mason et al. 1999; Desai et al. 2006a), or (3) preferential injection of flare suprathermals at quasi-perpendicular shocks (e.g., Tylka et al. 2005). In the remainder of this section, we discuss the observational evidence and arguments used in favor for each of these scenarios.

#### Direct flare contributions

**Fig. 12:**
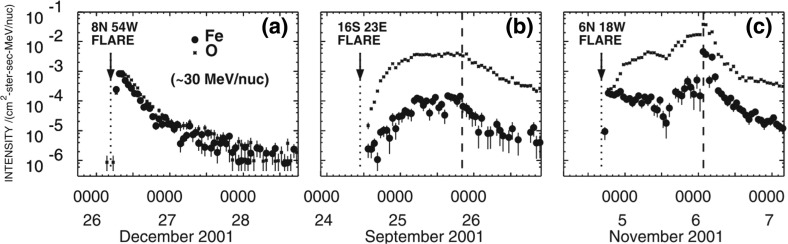
Fe and O intensity-time profiles at ${\sim }30\,\mathrm{MeV/nucleon}$ during three large gradual SEP events measured by ACE/SIS. Image reproduced with permission from Cane et al. (2003), copyright by AGU

Cane et al. (2003, (2006) examined temporal variations in the intensity profiles of Fe and O and in the Fe/O ratio during individual SEP events above ${\sim }25\,\mathrm{MeV/nucleon}$ (see Fig. 12) and proposed that many large SEP events are a mixture of flare-accelerated and shock-accelerated populations. As shown in Fig. 12, Cane et al. argue that the relative contributions from flares and CME shocks at a given energy depend on properties of the flare, the strength of the CME shock, and the observer’s magnetic connection to the flare site.

In this scenario, well-connected western hemisphere events associated with longer duration flares and weaker CME shocks are dominated by flare-accelerated material above ${\sim }10\,\mathrm{MeV/nucleon}$, causing the intensities to rise promptly and the Fe/O to increase significantly over the corresponding SW value, as in Fig. 12a; this effect could also account for the increasing Fe/O ratios with increasing energy during the August 24, 2002 event. On the other hand, eastern hemisphere SEP events (Fig. 12b) have broader time profiles and Fe/O ratios similar to or lower than the corresponding SW values. Finally, central meridian events (Fig. 12c) have two components: a prompt rise accompanied by higher Fe/O ratios due to flare particle contributions earlier in the event, followed by a larger IP shock-accelerated component with Fe/O $\le 0.1$ superposed on the flare population. Thus, in the Cane et al. scenario, the CME shock during the April 21, 2002 event is sufficiently strong to accelerate ${>}10\,\mathrm{MeV/nucleon}$ particles at 1 AU and cause the Fe/O to decrease with increasing energy.

It is worthwhile mentioning that the Cane et al. (2003, (2006) two-component assertion essentially implies that the ${>}10\,\mathrm{MeV}$ proton intensities in some SEP events should be completely dominated by either the flare or the CME shock associated component. In particular, this suggests that some SEP events with CMEs too slow to drive fast and wide shocks might still be associated with significant ${>}10\,\mathrm{MeV}$ proton intensity increases due to the flare component. However, Kahler et al. (2000) searched for SEP events in association with posteruptive arcades following CMEs, and identified 30 CME-arcade cases with no detectable increases in the ${>}10\,\mathrm{MeV}$ proton intensities. While this study does not rule out pre-CME flare contributions to large gradual SEP events, it does provide evidence that magnetic reconnection in posteruptive coronal arcades do not contribute to large gradual SEP events.

More recently, Cane et al. (2010) examined the association between SEP properties, such as the peak intensities, time-intensity profiles, the electron-to-proton and Fe/O ratios, in 280 solar proton events that extended above $\sim$25 MeV during 1997–2006 and properties of the accompanying flare, CME, and radio emissions. They found that the events do not separate into groups, as expected from the simple two-class picture, but instead exhibit continuous distributions. Based on these results, Cane et al. (2010) concluded that both flare and CME shock acceleration could contribute in the majority of the largest SEP events.

#### Suprathermal seed populations: $^{3}$He and heavy ion abundances

**Fig. 13:**
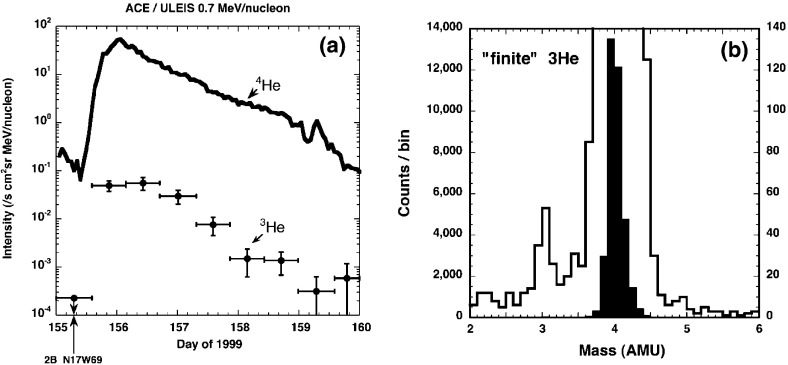
**a** Temporal profiles of ${\sim }0.7\,\mathrm{MeV/nucleon}~^{3}$He and $^{4}$He ions in a large CME-related SEP event. **b** 0.5–2.0 MeV/nucleon He mass histogram obtained during several large SEP events. The *right scale* corresponds to the open histogram. Image reproduced with permission from Mason et al. (1999), copyright by AAS

Understanding remotely accelerated large SEP events is difficult because the acceleration processes occur near the Sun, and other effects (e.g., propagation to 1 AU) have to be considered. Nevertheless, the mere presence of rare tracer ions like $^{3}$He can be used to identify the origin of the seed population. Figure 13a shows time-intensity profiles for 0.5–$2.0\,\mathrm{MeV/nucleon}$ $^{3}$He and $^{4}$He ions in a large CME-related SEP event that occurred on June 4, 1999 (from Mason et al. 1999). The temporal profiles of the two species are remarkably similar, which indicates that they probably share the same acceleration and transport history. In this particular event, the $^{3}$He is enriched by a factor of $16\,\pm \,3$, while the Fe/O ratio (not shown) is simultaneously enhanced by about a factor of 10 relative to the corresponding SW values. Since the M/Q ratio for Fe is larger than that of O while that of $^{3}$He is smaller than that of $^{4}$He, these results cannot be reconciled with M/Q- or rigidity-dependent acceleration mechanisms in which the shock operates solely on a SW-like seed population.

The event in Fig. 13a was selected from a list of large CME-related NOAA Space Environment Center events that produced significant 10 MeV proton intensity enhancements at 1 AU. A substantial fraction (${\sim }50\,\%$) of these events had $^{3}$He enrichments (e.g., Mason et al. 1999; Wiedenbeck et al. 2000). Figure 13b shows the low energy He mass histogram from several such events. Notice that the $^{3}$He is clearly resolved from $^{4}$He and the background. These enhancements are attributed to the presence of residual or remnant flare-accelerated $^{3}$He-rich suprathermal material in the seed population for CME-driven shocks near the Sun.

**Fig. 14:**
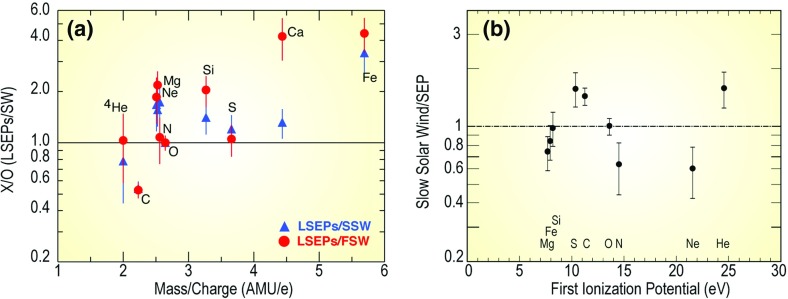
**a** Average heavy ion abundances at ${\sim }0.32$–$0.45\,\mathrm{MeV/nucleon}$ in 64 large SEP events events relative to those measured in the fast and slow solar wind, normalized to oxygen and plotted versus M/Q (adapted from Desai et al. 2006a). **b** Average abundances measured in the slow solar wind divided by those measured in 40 large CME-related SEP events above ${\sim }5\,\mathrm{MeV/nucleon}$, plotted versus the FIP of each element (adapted from Mewaldt et al. 2002)

ACE measurements have also allowed us to explore whether the heavier ions originate from the SW peak. Desai et al. (2006a) compared the ${\sim }0.4\,\mathrm{MeV/nucleon}$ heavy ion abundances averaged over 64 large SEP events with those measured in the fast and slow SW (see Fig. 14a) as a function of the ion’s M/Q ratio. In Fig. 14b, Mewaldt et al. (2002) normalized the ${>}5\,\mathrm{MeV/nucleon}$ abundances averaged over ${\sim }40$ large SEP events to those measured in the SW and plotted them versus the first ionization potential (FIP). The figure shows that SEP abundances at both energies are not organized in any systematic fashion by the M/Q ratio or the FIP. Rather, the heavy ion abundances are scattered randomly about the 1:1 line. Kahler et al. (2009) directly compared SEP abundances with corresponding abundances in three different types of background solar wind in which the SEPs were observed; this study found no differences in SEP composition among the three types of SW; fast, slow, and intermediate. These results are yet another indication that the material accelerated in large SEP events is quite distinct from that measured in the solar wind; and therefore, the SEP heavy ions are unlikely to originate from the bulk solar wind.

**Fig. 15:**
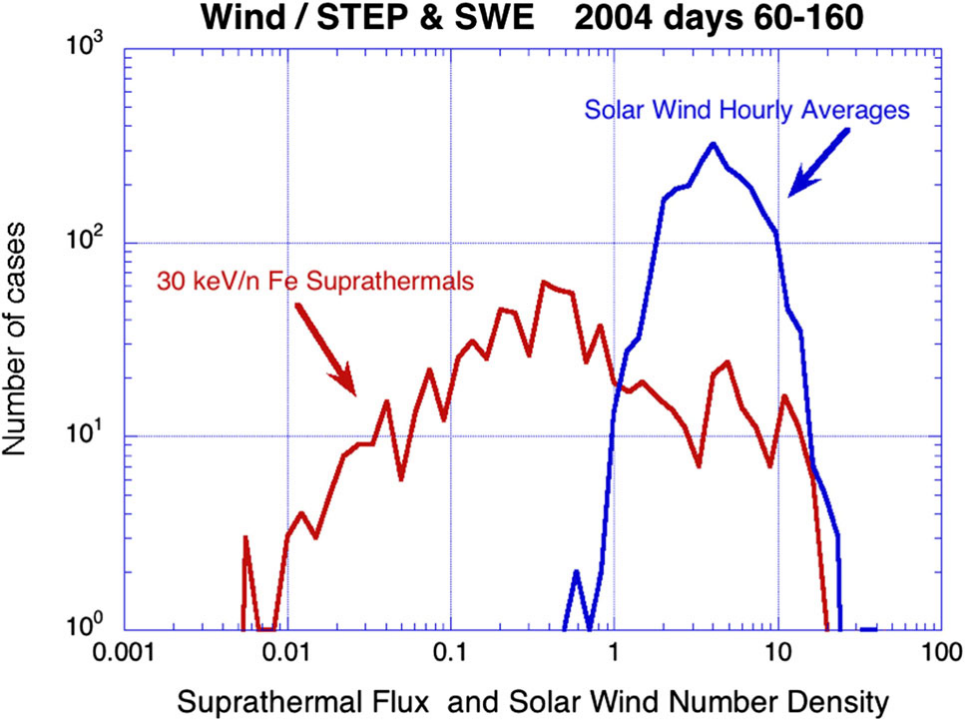
Hourly averaged intensity of suprathermal ${\sim }30\,\mathrm{keV/nucleon}$ Fe (*red*) and number density of solar wind Fe (*blue*) during a 100-day period in 2004. Image reproduced with permission from Mason et al. (2005), copyright by AIP

Figure 15 shows that, over a 100-day interval, solar wind densities (blue) vary only by about a factor of 10, while the 30 keV/nucleon suprathermal Fe intensity (red) varies by nearly three orders of magnitude. This large variation could play a critical role in determining the peak intensities and SEP kinetic energies in SEPs that show an extremely large range for CMEs of the same speed, mass, or kinetic energy, as seen in Figs. 7, 8 and 9.

**Fig. 16:**
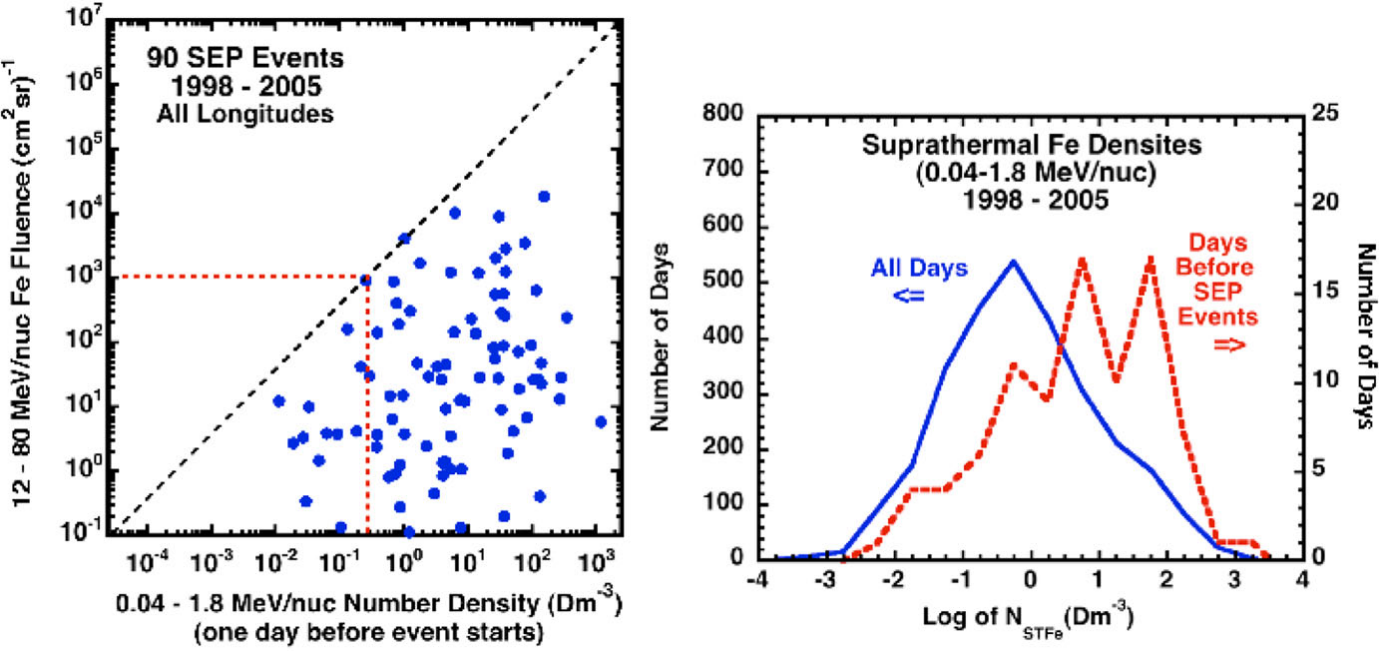
*Left* Fluences of 12–80 MeV/nucleon Fe in large SEP events from solar cycle 23 versus the suprathermal Fe density averaged over the day before the SEP event. The *dashed black line* is 3333 times the number-density scale. *Right* Histogram of daily averaged suprathermal Fe densities for all days from March 1998 to December 2005 (*left scale*) compared to a histogram of suprathermal Fe densities, measured one day before the associated SEP events (*right scale*). Image reproduced with permission from Mewaldt et al. (2012b), copyright by AIP

Mewaldt et al. (2012a) investigated whether pre-existing suprathermal ion densities are related to SEP fluences. Figure 16 (left) compares the Fe fluence in 90 large SEP events, defined as events with ${>}12\,\mathrm{MeV/nucleon}$ Fe fluences ${>}0.1/(\mathrm{cm^{2}\ sr}$), from 1998–2005, with the number density of suprathermal Fe at 1 AU one day before the SEP event occurred, i.e., on the day before the solar flare and CME eruption. Days with high fluences [e.g., ${>}10^{3}\,\mathrm{Fe/(cm^{2}\,sr}$); red dashed line] only occur when the density of pre-existing suprathermal Fe was ${>}0.3\,\mathrm{Dm}^{-3}$. Figure 16 (right) shows that the suprathermal Fe densities are generally significantly greater before the occurrence of these large SEP events compared to all other days, perhaps indicating that the presence of high-density suprathermal Fe is necessary for SEP events with large Fe fluences to occur. Mewaldt et al. (2012a, (2012b) speculated that the inner heliosphere served as a reservoir of suprathermal ions from a variety of sources, including $^{3}$He- and Fe-enriched material accelerated in flares and suprathermal material accelerated at previous CME shocks. This material is subsequently re-accelerated by the CME shock that produced the large SEP event (also see Mason et al. 1999; Desai et al. 2006a).

#### Shock geometry and compound seed populations

**Fig. 17:**
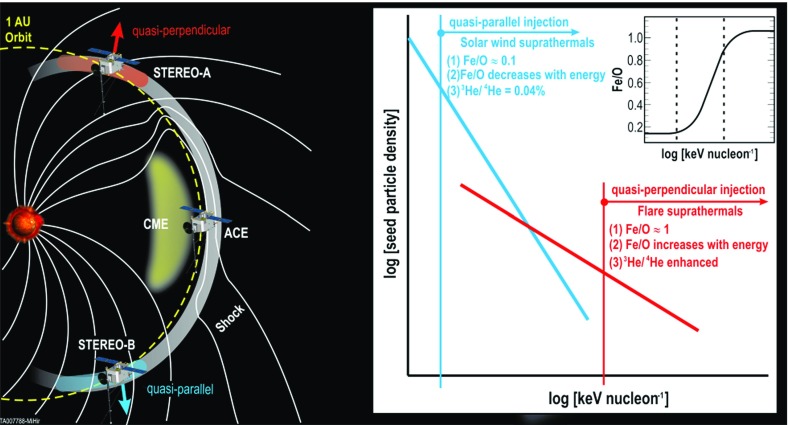
*Left* Schematic of a CME-driven shock as seen at azimuthally-separated 1 AU spacecraft illustrating the variation in shock obliquity and the corresponding regions of variable injection threshold speeds (adapted from Zank et al. 2006). *Right* According to the Tylka and Lee (2006) model, the suprathermal seed population for shock-accelerated ESPs and SEPs comprises both coronal (or solar wind) and flare-accelerated ions. Flare suprathermals are more likely to be accelerated at quasi-perpendicular shocks with higher injection thresholds. The inset shows the energy-dependence of Fe/O ratio in the accelerated population (adapted from Tylka et al. 2005)

In contrast to Cane et al. (2003, (2006), Tylka et al. (2005) suggest that the extreme Fe/O behavior in the SEP event in Fig. 11 could occur if different orientations of the shock normal relative to the upstream magnetic field result in the injection and acceleration of vastly different seed populations. In this scenario, illustrated in Fig. 17, Tylka et al. assume that perpendicular shocks are unable to accelerate low-energy ions and have a higher injection threshold, therefore they predominantly accelerate the Fe-rich, suprathermal-through-energetic particle population associated with solar flares (e.g., Forman and Webb 1985). This causes the Fe/O ratio to increase with increasing energy as in the August 24, 1998 SEP event. On the other hand, Tylka et al. further assume that since quasi-parallel shocks have a lower injection threshold energy, they can accelerate the ambient solar wind (or coronal suprathermal ions), which causes the Fe/O ratio to decrease with increasing energy as in the April 21, 2002 SEP event. While there is some theoretical justification for the assumptions regarding the existence of injection threshold energy in DSA processes, this issue is not simple; in fact, there may exist situations where there is no dependence of the injection threshold energy on the shock-normal angle. This issue is discussed further in Sect. 7.2.6.

While the Tylka et al. (2005) model uses the shock orientation near the Sun and requires the presence of suprathermal flare seed populations, it is essentially independent of the longitude of the observer relative to that of the source or the flare location. In contrast, the observer’s relative longitude is a critical feature of the Cane et al. (2003, (2006) scenario. Based on large enhancements in the Fe/O during the initial phases of two large SEP events observed at Wind and Ulysses when the two s/c were separated by ${>}60^{\circ }$ in longitude, Tylka et al. (2013) argue that the initial Fe/O enhancements cannot be construed as evidence for direct flare contributions, but rather that such enhancements are better understood in terms of radial diffusion and transport-related effects (see Sect. 2.8). Likewise, the Mason et al. (1999) scenario also requires that CME-driven shocks have access to flare material en route to 1 AU, but it does not depend on the relative longitude of the observer. Thus, while somewhat distinct, both the Tylka et al. (2005) and Mason et al. (1999) scenarios require the re-acceleration of flare suprathermals at CME-driven shocks.

#### Constituents of suprathermal seed populations

While there is little doubt that flare suprathermals can occasionally contribute to the seed population for large gradual SEPs, it is still unclear just how much of the source material is actually composed of flare-accelerated ions. Desai et al. (2006a), for instance, compared the average ${\sim }0.38\,\mathrm{MeV/nucleon}$ heavy ion abundances in 64 large SEP events to event-averaged, heavy-ion abundances in ${^3}$He-rich SEPs (Mason et al. 2004) and in large gradual SEP events at ${\sim }5$–$12\,\mathrm{MeV/nucleon}$ (Reames 1995a) to show that the average large SEP seed population could comprise up to ${\sim }75\,\%$ flare-rich material and ${>}25\,\%$ ambient coronal material. In contrast, Mewaldt et al. (2006) concluded that, on average, the remnant or residual suprathermal Fe densities observed during quiet days prior to the occurrence of several large Fe-rich SEP events were not sufficient to account for the observed ${\sim }10\,\mathrm{MeV/nucleon}$ Fe fluences, and that an additional source of Fe was necessary; possible sources considered were the co-temporal flare, lower-energy material, suprathermal tails, interplanetary coronal mass ejection (ICME) material in the case of multiple CMEs, and previous gradual and IP shock events.

**Fig. 18:**
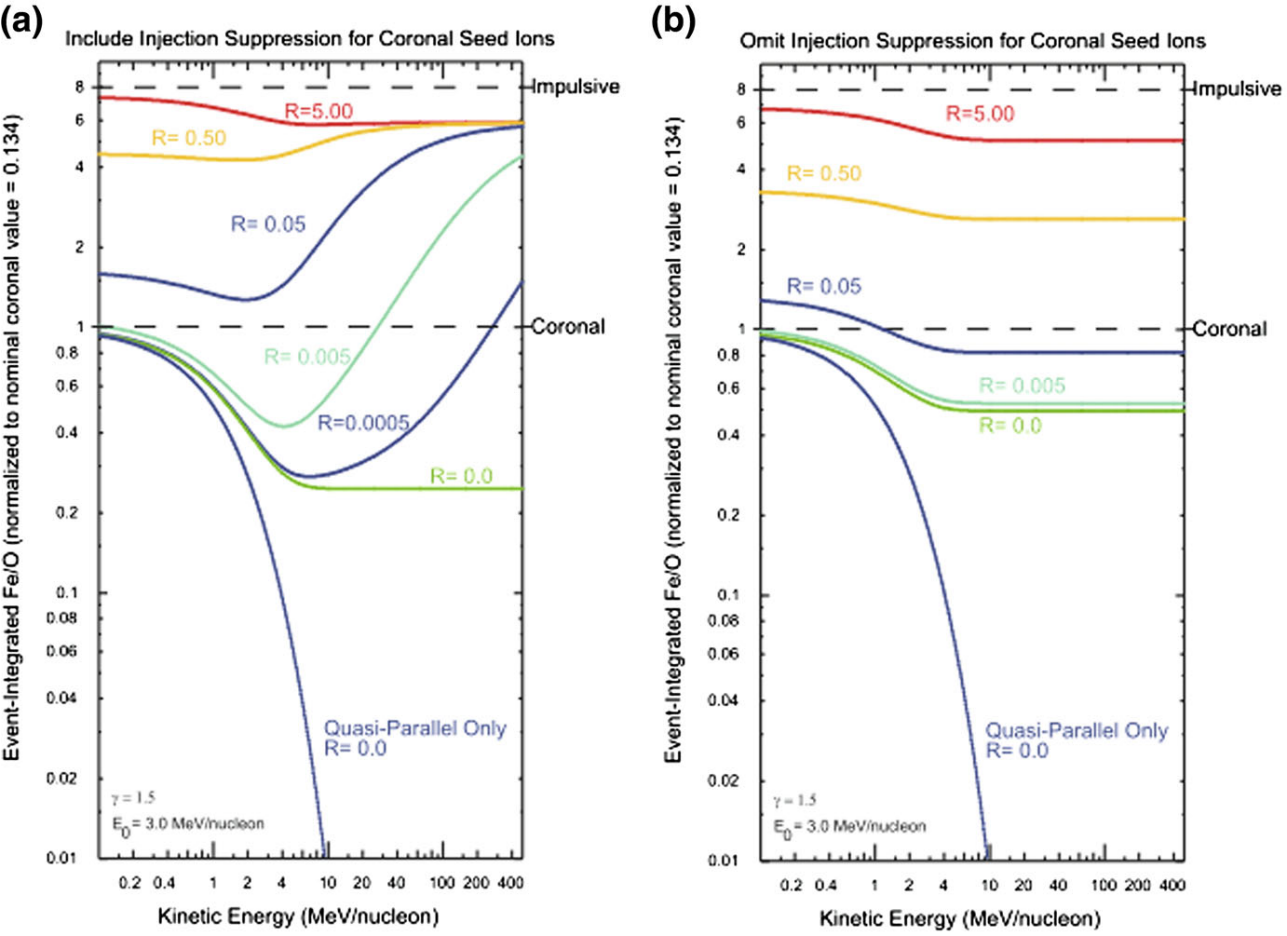
Model calculations for the Fe/O ratio versus energy. The Fe/O ratio is normalized to 0.134, which is taken as typical of the coronal population. The *bottom curve* in both panels shows the quasi-parallel case in which the spectra are averaged over $0^\circ \le \theta _{Bn}\le 60^\circ$, while the *rest of the curves* represent quasi-perpendicular shocks where the spectra are averaged over the full range of $0^\circ \le \theta _{Bn}\le 90^\circ$. The calculations are performed by assuming different fractions of the flare component in the seed population, as specified by the parameter *R* (see text for more details). Other energetic particle parameters are fixed: spectral index $\gamma =1.5$ and $E_{0}=3.0\,\mathrm{MeV/nucleon}$. **a** Injection of ions from the coronal component is suppressed at quasi-perpendicular shocks. **b** Same calculations without coronal seed suppression. Image reproduced with permission from Tylka and Lee (2006), copyright by AAS

In an attempt to account for the dramatically distinct behavior of Fe/O in Fig. 11, Tylka and Lee (2006) formalized the ideas put forward by Tylka et al. (2005) in an analytical model. The results of these model calculations are shown in Fig. 18. Two cases are shown: (a) includes injection threshold for quasi-perpendicular shocks, i.e., suppresses the injection of coronal seed population at quasi-perpendicular shocks; and (b) no injection threshold at quasi-perpendicular shocks. The parameter $R\equiv C_{\mathrm{Fe,Flare}}/C_{\mathrm{Fe,Coronal}}$, reflects the relative strengths of the remnant flare and coronal source contributions at a parallel shock, where seed ions from both populations are injected with equal efficiency.

The April 21, 2002, SEP event in Fig. 11 is best represented by $R \sim 0$, while the August 24, 2002, SEP event is best represented by $R \sim 0.05$. In the August 24 event, substantially different Fe/O ratios in the two components imply that $C_{\mathrm{Fe,Flare}}/C_{\mathrm{Fe,Coronal}}=0.79$. Thus it appears that the increase in Fe/O ratio above ${\sim }10\,\mathrm{MeV/nucleon}$ in some SEP events may reflect the fact that the seed population comprises substantial amounts of flare (${\sim }40\,\%$) material mixed with the ambient coronal population. Strikingly, this simple analytical model could also account for the Q/M-fractionation of ${\sim }12$–$60\,\mathrm{MeV/nucleon}$ event-averaged C–Fe abundances, as originally reported by Breneman and Stone (1985). Above ${\sim }1\,\mathrm{MeV/nucleon}$, the Tylka and Lee (2006) model calculations were in reasonable agreement with the observed behavior of: (1) Fe/O versus energy, (2) the $^{3}$He/$^{4}$He ratio, and (3) the mean ionic charge state of Fe. However, the same model was unable to reproduce observations below ${\sim }1\,\mathrm{MeV/nucleon}$. Tylka and Lee (2006) suggest that this discrepancy occurred probably because their calculations, which averaged the effects of $\theta _{Bn}$ over all shock normal angles, are likely to be valid near the Sun but not for the lower-energy SEPs, most of which are probably accelerated later during the CME shock’s transit from the Sun to the observer.

More recently, Reames (2014) used the systematic correlation between the enhancements and depletions in the ${\sim }3.2$–$5\,\mathrm{MeV/nucleon}$ Fe/O and the ${\sim }2$–$15\,\mathrm{MeV/nucleon}$ Fe spectral index in 54 large SEP events to argue that most of the temporal and spatial variations in the abundances and energy spectra of heavy ions occur *after* acceleration and are therefore due to rigidity-dependent scattering during transport. Reames (2014) concludes that the strongest effects of the seed population occur above the spectral knee energies, which depend on both the species Q/M ratio and $\theta _{Bn}$ (see Tylka and Lee 2006), and that even a small amount of flare material in the seed population can have a large effect above $\sim$10s MeV/nucleon, where spectral knees become dominant. Using these results, Reames (2014) measured the ${\sim }2$–$15\,\mathrm{MeV/nucleon}$ heavy ion abundances using appropriate sampling and averaging time intervals to retrieve compositional information about the coronal source material, which is remarkably similar to that reported earlier by Reames (1995a).

#### Ionic charge states in gradual SEPs

**Fig. 19:**
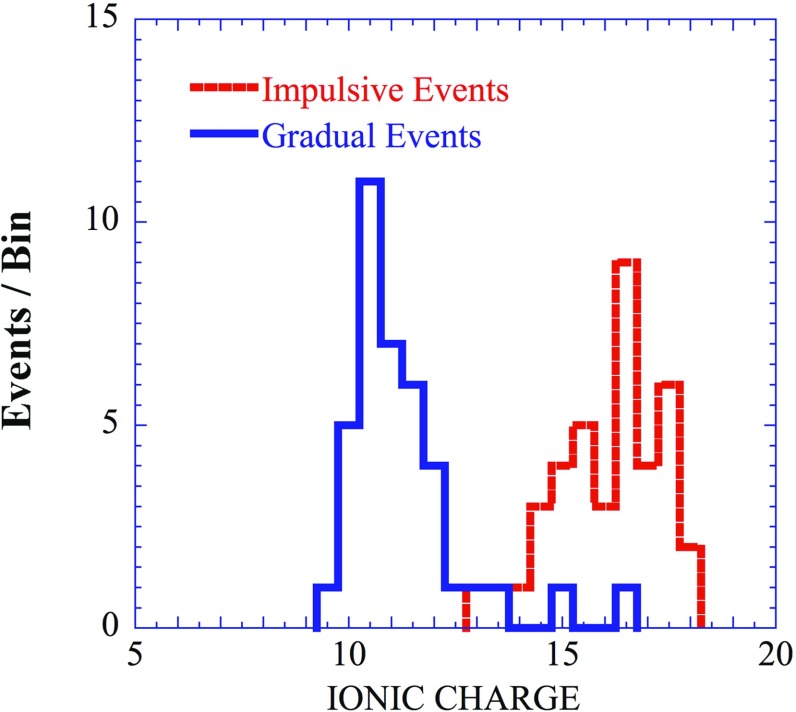
*Left* Average ionic charge of Fe in the energy range 0.18–0.24 MeV/nucleon in ${\sim }40$ impulsive and ${\sim }40$ gradual SEP events; see text for details. *Right* Mean ionic charge Q$_{\mathrm{Fe}}$ at 0.18–0.25 MeV/nucleon versus that at 0.36–0.43 and 28–65 MeV/nucleon. Image reproduced with permission from Klecker et al. (2007), copyright by Springer

Ionic charge states provide another key diagnostic of SEP acceleration locations and conditions, as well as of the source populations. Since the acceleration and transport processes depend on the ion’s M/Q-ratio and also on its velocity, variations in the mean ionic charge of heavy ions, e.g., Fe at ${\sim }1\,\mathrm{MeV/nucleon}$, are often used to distinguish flare-accelerated material from CME-shock accelerated ions (e.g., Reames 1999). In particular, gradual SEPs have mean Fe charge states, Q$_{\mathrm{Fe}}$, consistent with coronal source temperatures of ${\sim }1.5$–$2\times 10^{6}\,\mathrm{K}$. In contrast, the $^{3}$He-rich or flare-associated SEPs have Q$_{\mathrm{Fe}}$ $\sim$15–20, consistent with charge-stripping in the low corona (e.g., see Fig. 19 (left) and review by Klecker et al. 2007; Dröge et al. 2006).

The Q$_{\mathrm{Fe}}$ in many large SEP events is essentially energy-independent up to few MeV/nucleon, which is consistent with CME shock acceleration in the tenuous high corona or in interplanetary space. This is because processes such as: (1) charge-changing effects resulting from ionization by thermal electrons and ions, (2) mixing of sources with different ionic charge distributions, and (3) M/Q-dependent energy spectra, do not significantly affect the ionic charge states as a function of energy (see e.g., Kovaltsov et al. 2001; Klecker et al. 2007).

However, ionic charge state measurements over an extended energy range from SAMPEX, ACE, and Wind during solar cycle 23 showed significant energy-dependent variations in individual large SEP events. For instance, below ${\le }1\,\mathrm{MeV/nucleon}$, typical observed values of Q$_{\mathrm{Fe}}$ were ${\sim }9$–12, which remained essentially constant with energy or, in some cases, increased with energy by up to 4 charge units (see Bogdanov et al. 2000; Möbius et al. 1999, 2000; Mazur et al. 1999). In general, these sub-MeV/nucleon Fe charge states are similar to those measured in the solar wind (Ko et al. 1999). Figure 19 (right) shows event-averaged values for Q$_{\mathrm{Fe}}$ in three energy ranges between 0.18 and 65 MeV/nucleon in several large SEP events. The figure shows that the largest variations and differences in these three SEP events are observed above ${\sim }10\,\mathrm{MeV/nucleon}$, with Q$_{\mathrm{Fe}}$ $\sim$15–20 (e.g., Leske et al. 1995; Labrador et al. 2005; Oetliker et al. 1997). These results challenged the previously held notion that Fe charge states are related only to an equilibrium plasma temperature reflecting that of the ambient solar corona.

Barghouty and Mewaldt (1999) developed a model in which the energy-dependence of Q$_{\mathrm{Fe}}$ in large SEP events occurs when charge-changing processes associated with ionization–recombination and energy-changing processes due to shock acceleration have similar timescales. This dynamic interplay results in an equilibrium that reflects an accelerated seed population with its own characteristic temperature and non-thermal energy spectrum. In contrast, when these two processes operate on vastly different timescales, Q$_{\mathrm{Fe}}$ is largely energy-independent and reflects both the pre-accelerated and accelerated populations. In contrast, Reames et al. (1999) developed a model in which large energy-dependent variations of Si and Fe charge states in the November 6, 1997 event occur primarily due to electron stripping in moderately dense coronal plasma during shock acceleration. We note that, even though the Tylka and Lee (2006) model was developed to explain the large heavy ion compositional variations and spectral features, it also predicts an energy-dependent increase in the mean ionic charge for quasi-perpendicular shocks that inject flare suprathermals. As noted above, this model is able to account for large SEP observations above ${\sim }10\,\mathrm{MeV/nucleon}$ but not below ${\sim }1\,\mathrm{MeV/nucleon}$. Alternatively, in the Cane et al. (2003, (2006) scenario, a direct flare component with high Fe charge states and high Fe/O ratios dominates above ${\sim }10$s MeV/nucleon, while the CME shock-accelerated component with coronal-like Q$_{\mathrm{Fe}}$ and Fe/O values dominates at lower energies.

To summarize, recent measurements of the energy dependence of ionic charge states in large SEP events have yielded important clues about: (1) conditions and locations of particle acceleration, (2) source populations, and (3) physical processes contributing to the charge-stripping processes. For instance, shock acceleration of a coronal seed population and electron impact ionization starting in the lower corona at ${\sim }1.5$–$2\,R_{S}$ can account for a large increase in Q$_{\mathrm{Fe}}$ with increasing energy between ${\sim }0.1$ and $1\,\mathrm{MeV/nucleon}$ (Kocharov 2006), i.e., at much lower energies than previously thought. In contrast, large SEP events with nearly constant Q$_{\mathrm{Fe}}$ below ${\sim }1\,\mathrm{MeV/nucleon}$ accompanied by a large increase in Q$_{\mathrm{Fe}}$ above ${\sim }10$s of MeV/nucleon and an enhancement in the Fe/O ratio point to contributions from both a coronal source and a highly ionized, heavy ion-enriched flare population.

Finally, as noted by Tylka et al. (2013), observations of highly charged Fe accompanying Fe/O abundance enhancements during the initial phases of large SEPs may provide evidence of direct flare contributions, because at $\sim$ MeV/nucleon energies the flare-accelerated Fe and O ions are nearly fully ionized, therefore Fe ions do not have significantly different M/Q ratios compared with O ions. In such cases, rigidity-transport related effects (see Sect. 2.8) cannot cause large enhancements in the Fe/O ratio. However, even in such situations, the flare-accelerated, highly charged Fe ions could be subsequently energized by the CME-shock (see Tylka and Lee 2006). The lack of instruments with sufficient sensitivity and geometric factor for measuring charge states in the $\sim$MeV/nucleon energy range during SEP event onsets, when the count rates are low, continues to fuel the ongoing controversy, i.e., do flares contribute directly to large SEP events above ${\sim }10$s MeV/nucleon or do they contribute to the seed population for further acceleration by the CME-driven shock.

### Scattering during acceleration

Following Zank et al. (2000), Cohen et al. (2005b) reported that the position of the breaks in the heavy ion spectra and the resulting energy-dependent behavior in the Fe/O ratio during the October–November 2003 SEP events can be understood in terms of leakage from the shock region, if the mean free path $\lambda _{\parallel }$ is proportional to a power-law with index $\alpha$ in ion rigidity (i.e., $\lambda _{\parallel }\propto (Mv/Q)^{\alpha }$, where *v* is the ion speed, and *M* and *Q* are the mass and charge in units of proton mass, $m_{p}$, and electronic charge, *e*, respectively.) Specifically, Cohen et al. (2005b) noted that the breaks in the energy spectra for different species should occur at the same value of the diffusion coefficient, $\kappa$, and used this to calculate a single value for $\alpha$ that allowed a scaling in kinetic energy per nucleon between an element ‘X’ relative to O as:

1 $$\begin{aligned} \frac{E_{X}}{E_{O}}=\left[ \frac{(Q/M)_{X}}{(Q/M)_{O}}\right] ^{2\alpha /(\alpha +1)}. \end{aligned}$$

The value of $\alpha$ was selected so that the abundance ratios between $\sim$0.3 and 30 MeV/nucleon were relatively constant.

**Fig. 20:**
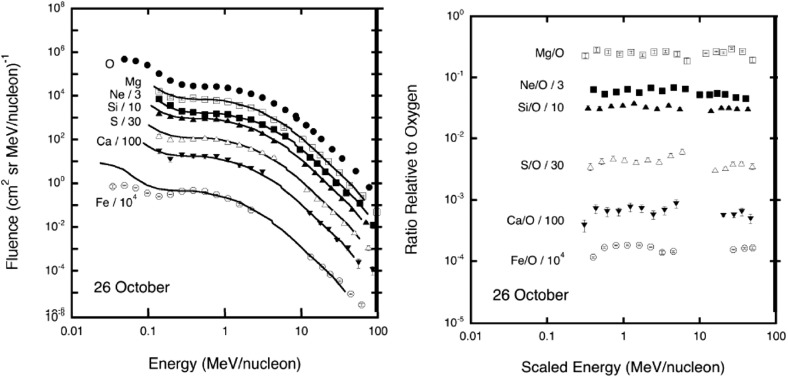
*Left* Event-integrated fluences of O, Ne, Mg, Si, S, Ca, and Fe plotted versus energy during the large SEP event on October 26, 2003. All the spectra except O and Mg have been scaled to better compare the spectral shapes. The *solid lines* are the oxygen spectra scaled appropriately in energy (see text). *Right* Abundance ratios relative to oxygen, calculated from the spectra shown on the left, plotted versus scaled energy. Image reproduced with permission from Cohen et al. (2005b), copyright by AGU

An example of this energy scaling technique is shown in Fig. 20 for the October 26, 2003, event where $\alpha =1$. Thus, the spectral behavior of Fe and O in the October 26, 2003, SEP event is better organized in terms of ion rigidity, as predicted by shock acceleration theory (see Zank et al. 2000). Cohen et al. (2005b) used this technique to infer the Q/M-dependence of the scattering mean free path in the vicinity of the shock where the ions were accelerated and suggested that such rigidity dependence is consistent with a source of enhanced wave turbulence near the shock.

### Interplanetary scattering

**Fig. 21:**
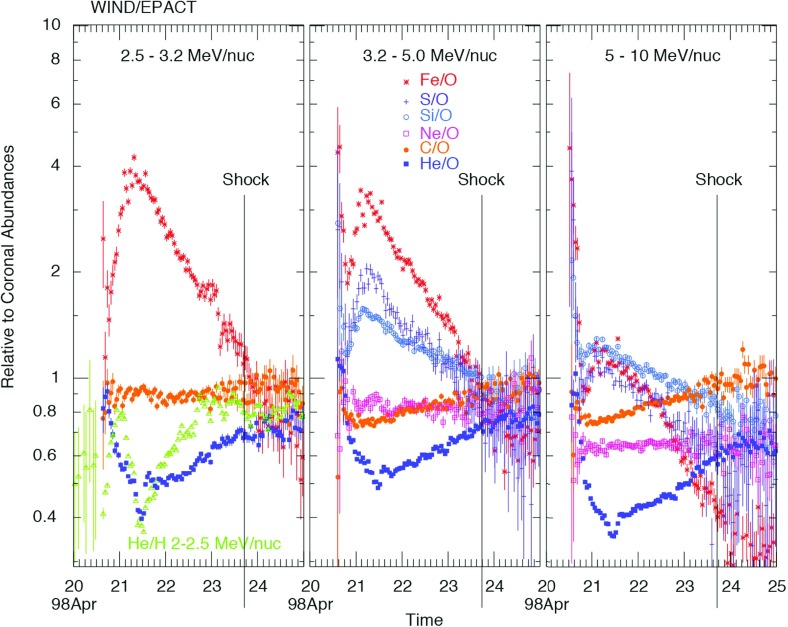
One-hour averaged elemental composition normalized to coronal values (Reames 1995a) measured by Wind/EPACT/LEMT during the April 20, 1998 SEP event. Image reproduced with permission from Tylka et al. (1999), copyright by AGU

Tylka et al. (1999) and Ng et al. (1999) modeled the energy spectra and systematic temporal evolution of the elemental abundances of ${\sim }5$–$10\,\mathrm{MeV/nucleon}$ He, C, O, Ne, Si and Fe ions in two large SEP events (e.g., Fig. 21) in terms of rigidity-dependent scattering by Alfvén waves generated by streaming energetic protons accelerated at CME-driven shocks. These studies showed that, when compared at the same kinetic energy-per-nucleon, elemental abundances such as Si/O and Fe/O exhibited strong enhancements during SEP event onsets because Si and Fe have higher M/Q (i.e., higher rigidity) values when compared with O, which allows them to escape from the scattering region near the shock more easily and to be observed earlier than O at a distant s/c. As the CME shock expands and propagates out into the heliosphere, its ability to accelerate particles and create waves declines, thereby causing a reduction in the Si/O and Fe/O ratios with time. Evidence for self-generated Alfvén waves comes from the opposite evolution of the ${\sim }2$–$10\,\mathrm{MeV/nucleon}$ He/H ratios, as seen in Fig. 21. Note that, although the relative M/Q values of Fe and O are similar to those of He and H, the He/H ratios at all energies drop at the start of the event, indicating that the scattering is due to a dynamic wave spectrum generated by streaming energetic protons rather than a background Kolmogorov-like wave spectrum (Ng et al. 1999).

**Fig. 22:**
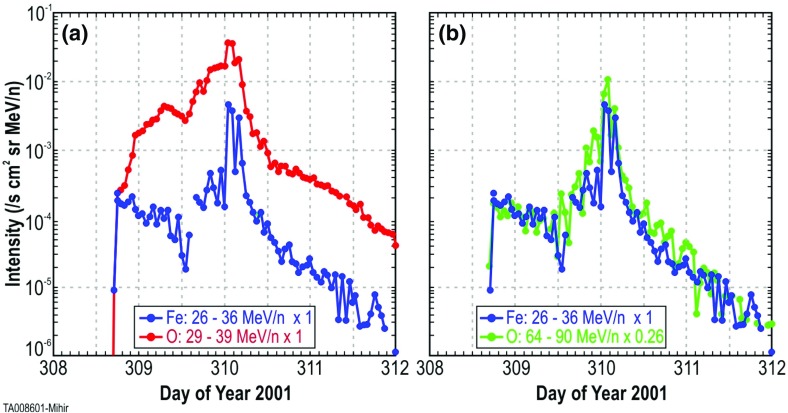
**a** Fe and O intensity-time profiles during the November 4, 2001, (DOY 308) large SEP event at ${\sim }35\,\mathrm{MeV/nucleon}$. **b** Time-intensity profiles for the event in (**a**), with O at $\sim$twice the kinetic energy of Fe (adapted from Mason et al. 2006, 2012)

Mason et al. (2006) pointed out that the dramatic variations in the Fe/O ratio at all energies between ${\sim }0.1$ and $60\,\mathrm{MeV/nucleon}$ vanish in ${>}70\,\%$ of the prompt western hemisphere SEP events if the Fe intensities are compared to O intensities at $\sim$ twice the Fe kinetic energy-per-nucleon. An example of such a comparison for the November 4, 2001, central meridian SEP event is shown in Fig. 22. Note that the O intensity compared at twice the Fe energy results in nearly indistinguishable time histories. Mason et al. (2006) attributed this behavior to rigidity-dependent scattering of particles as they propagate through the corona and the interplanetary medium.

**Fig. 23:**
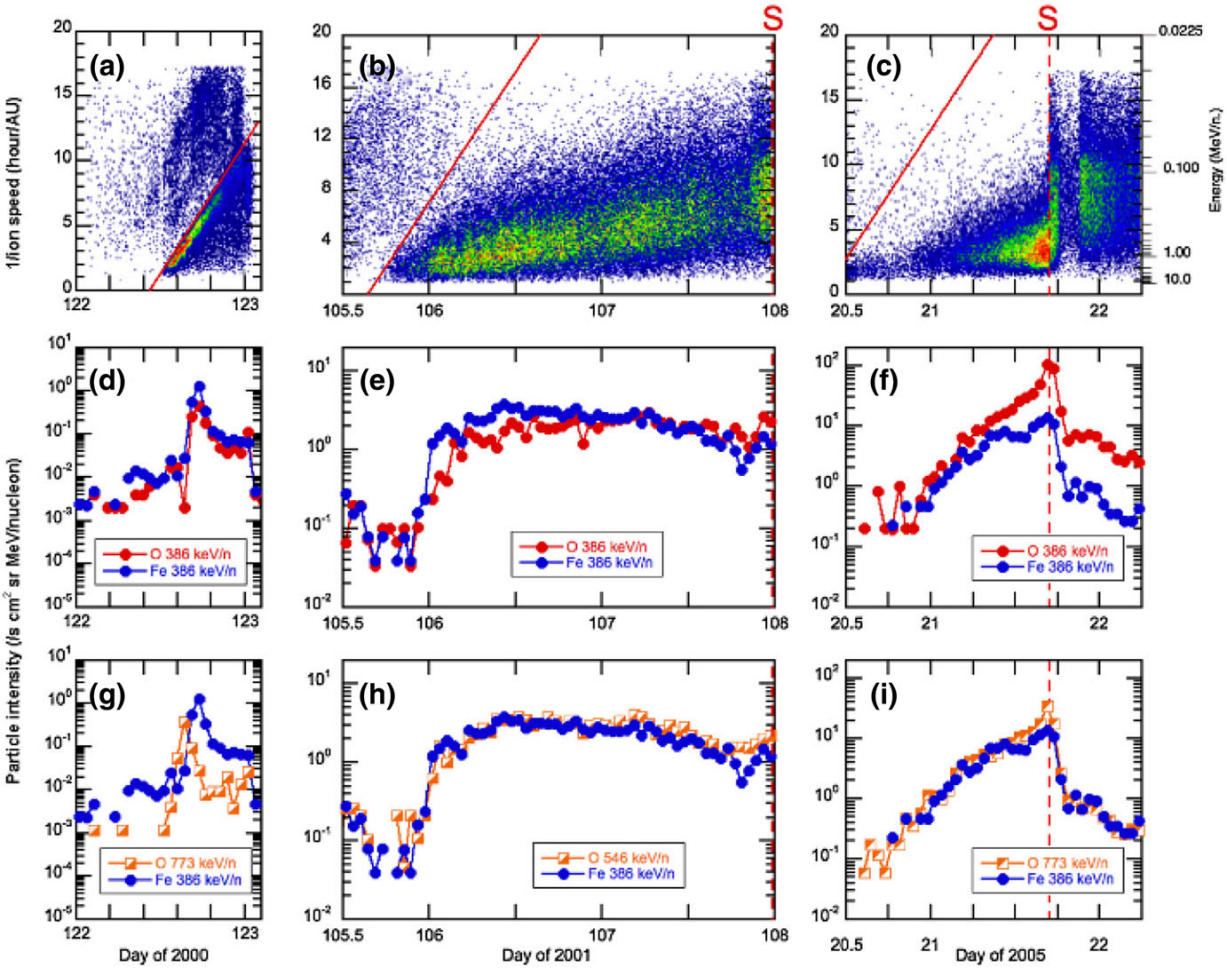
Columns show low energy ion data for three SEP events observed on: **a** May 1 (DOY 122), 2000; **b** April 21 (DOY 105), 2001; and **c** January 20 (DOY 20), 2005. **a**–**c** Spectrograms for 6–80 AMU ion arrivals plotted as 1/v versus time; *red diagonal lines* show arrival pattern for pure velocity dispersion along a 1.2 AU IMF line for particles injected at the time of the associated X-ray flare; *red dashed vertical lines* marked S show times of shock passage. **d**–**f** 386 keV/nucleon O and Fe intensity profiles during the events. **g**–**i** Fe intensities at 386 keV/nucleon from the (**d**), (**e**), and (**f**) compared with O at 773 keV/ nucleon in (**g**) and (**i**) and at 546 keV/nucleon in (**h**). Image reproduced with permission from Mason et al. (2012), copyright by AAS

Figure 23 illustrates the temporal behavior of Fe and O ions in different types of SEP events. Figure 23a–c shows particle arrival spectrograms of 6–80 AMU ions plotting 1/ion speed versus time, which, for pure velocity dispersion propagation along a typical 1.2 AU interplanetary field line from the Sun, produces arrival times along the red diagonal lines in the panels. The spectrogram color scales peak at red for the most intense periods, with separate scales for each plot. Figure 23d–f shows hourly averaged O and Fe intensities at ${\sim }386\,\mathrm{keV/nucleon}$. Figure 23a, d shows a narrow pulse of heavy ions with arrival times consistent with pure velocity dispersion from the Sun along a 1.2 AU nominal field line with release at the time of the associated X-ray flare (Kahler 2001). Events of this type are the so-called impulsive SEPs, and have enrichments of $^{3}$He and heavy ions (Mason et al. 2002). In such events, Fe and O ions with the same kinetic energy-per-nucleon or speed arrived simultaneously, as can be seen from Fig. 23d where the two profiles overlap at the same energy/nucleon (Mason et al. 2004).

Figure 23b, c, e and f shows examples of two CME-shock associated events. Figure 23b, c shows that low-energy heavy ions arrive much later than that expected from the diagonal line. Both velocity dispersion events in Fig. 23a, b are remotely accelerated near the Sun. Figure 23e shows that, during the rise phase, the 386 keV/nucleon Fe ions arrived several hours earlier than O, therefore the Fe/O ratio decreased later on day 107. Figure 23h shows that Fe and O intensities nearly match (yielding constant Fe/O ratios) when the Fe intensity is compared with that of O at ${\sim }1.4$ times the Fe energy (O intensity is renormalized by ${\sim }2.2$).

Finally, Fig. 23c, f, i shows a third type of behavior. The spectrogram for this event shows that at low-energies, there is no evidence of an SEP event onset and that instead, there is a dispersionless arrival of locally accelerated ESPs coincident with the CME-driven IP shock (dotted red line, marked S) on day 21, 2005. Note that ACE/SIS did observe a rapid increase in the high energy (${>}10\,\mathrm{MeV/nucleon}$) particle intensities (Mewaldt et al. 2005c; Reames 2009a) during this period. In contrast, the ${<}1\,\mathrm{MeV/nucleon}$ ions show no initial increase; the intensities increased gradually and peaked when the CME shock passed 1 AU at $\sim$16:45 on day 21. Figure 23f also shows that the Fe/O ratio decreased during the shock-associated period. Figure 23i shows that if the Fe intensity is compared to the O intensity at twice the kinetic energy-per-nucleon, the differences in the O and Fe profiles are markedly reduced, although there is still a decrease in Fe/O close to the shock passage (the O intensity has been renormalized by a factor of 0.5).

Thus, the energy scaling technique used by Cohen et al. (2005b) can flatten the energy-dependent behavior of the event-integrated Fe/O and also diminish the dramatic time variations in the Fe/O ratio during some SEP events. To explore the physical process involved, Mason et al. (2012) modeled the rise phases in 17 large SEP events and showed that the temporal evolution of Fe/O can be reasonably fitted by a state-of-the-art model where the differences in the transport of Fe versus O are due to the slope of the turbulence spectrum of the IMF. In summary, comparisons between SEP observations and modeling results are consistent with the notion that, at 1 AU, SEP composition, spectra, and temporal variations are heavily influenced by scattering and diffusion during acceleration and transport from the Sun through the corona and the interplanetary medium (e.g., Tylka et al. 1999; Ng et al. 1999; Mason et al. 2006; Tylka et al. 2013).

### Streaming limits

**Fig. 24:**
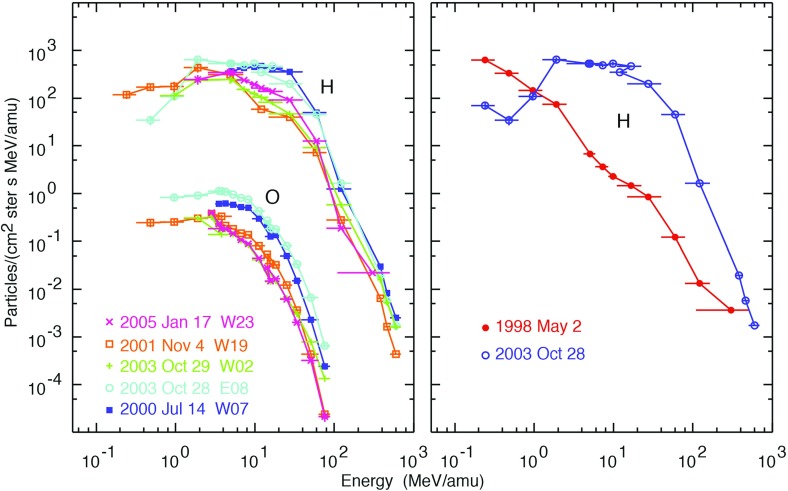
*Left* Turn-overs in the energy spectra of H and O in 5 large GLEs. *Right* Proton spectra in 2 GLEs with large differences in proton intensities at ${\sim }10\,\mathrm{MeV}$. Image reproduced with permission from Reames and Ng (2010), copyright by AAS

Proton intensities near $\sim$few MeV/nucleon exhibit energy-dependent upper bounds or plateaus regardless of the solar longitudes of the progenitor CMEs (Reames 1990). This effect has been predicted and modeled by theoretical studies and self-consistent numerical calculations of wave generation or amplification by shock-accelerated protons escaping or streaming away from the near-Sun CME shock (e.g., Lee 1983, 2005; Ng et al. 2003, 2012). The idea here is that particles accelerated later are scattered and trapped near the shock by the Alfvén waves generated by the high-energy protons accelerated earlier. This trapping and scattering causes the lower-energy particle intensities at 1 AU, or at other locations well away from the acceleration site, to increase more slowly until they are throttled and reach the so-called streaming limit. Examples of this near-equilibrium effect on the particle energy spectra are shown in Fig. 24. Here, high intensities of streaming ${\sim }10\,\mathrm{MeV}$ protons produce waves that scatter both the ${\sim }1\,\mathrm{MeV}$ protons and the lower energy O ions and suppress their intensities, causing the spectra to turn over during the October 2003 SEP event. In contrast, the ${>}2$ orders of magnitude lower ${\sim }10\,\mathrm{MeV}$ proton intensities during the May 1998 SEP event do not generate wave growth (Reames and Ng 2010), and the spectra continue as power laws down to lower energies.

While establishing streaming limits could serve as a practical means to model and predict the worst-case proton fluxes and hence the associated radiation hazard during a given SEP event, the situation is far more complex because of the non-linearity of wave-particle interactions, interplay between the intensities at different energies, trapping of particles near the shock, and magnetic connection between the near-Sun CME shock and the observer. Indeed, the trapping of ${\sim }10\,\mathrm{MeV}$ protons near the CME shock, as well as mirroring by plasma structures beyond 1 AU, are invoked to account for cases where the proton intensities exceed the equilibrium-case streaming limits in some SEP events; but this scenario does not appear to account for all cases where the proton intensities are greater than the theoretical streaming limits (e.g., Lario et al. 2008, 2009).

### Electron observations in large gradual SEP events

Though discovered in the 1960s, the origin of and link between energetic solar electron events and large gradual SEP ion events have been somewhat elusive. Lin (1970, (1974) classified solar flare events in terms of the associated emission of non-relativistic (energy ${<}100\,\mathrm{keV}$) electrons observed at 1 AU into three groups: (1) small flares with no particle or electromagnetic emission; (2) small flares with low energy electrons accompanied by type III and microwave radio bursts and hard X-ray bursts; and (3) large flares associated with relativistic proton and electron generation, type II and IV radio bursts, and intense microwave and X-ray emission. Simnett (1974) pointed out that, compared with the scatter-free transit time of ${\sim }10$ min along the Archimedean spiral IMF of length 1.2 AU, the maximum of the H$\alpha$ flare and the arrival of the first relativistic electrons (energy ${>}1\,\mathrm{MeV}$) at 1 AU was typically delayed by around 30 min.

These earlier results were confirmed by studies of Krucker and Lin (2000a, (2000b) and Simnett et al. (2002). In addition, these studies clarified the relationship between SEP ion and electron events. Specifically, it is now widely accepted that the Lin (1970) class (2) events are associated with impulsive or $^{3}$He-rich SEP events and are therefore most likely produced during the solar flare. In contrast, the larger, delayed electron events are associated with the escape or release of electrons due to the presence, propagation, and acceleration at CME-driven shocks, which are also responsible for accelerating the large gradual SEP ion events (Reames 1988b, 2013; Reames and Stone 1986; Reames et al. 1985, 1990; Simnett et al. 2002). Studies by Klein and Posner (2005) and Posner (2007) emphasize the use of early onsets and intensities of relativistic electrons to forecast the intensity of the associated SEP proton events.

Further support for the existence of two distinct types of electron-associated SEP events is provided by the study of Cliver and Ling (2007) who found that the peak intensities of ${\sim }1\,\mathrm{MeV}$ electrons and ${\sim }10\,\mathrm{MeV}$ protons in SEP events could be grouped into two distinct populations—one associated with the $^{3}$He-rich and heavy nuclei-rich, smaller impulsive SEP events, and the other with the larger, proton-rich gradual SEP events. Applying this two-class distinction for the SEP electron events may also shed some light on the puzzling multi-spacecraft and broad longitudinal distribution (see Fig. 68) reported by Wibberenz and Cane (2006). These observations are discussed in more detail in Sect. 8.

**Fig. 25:**
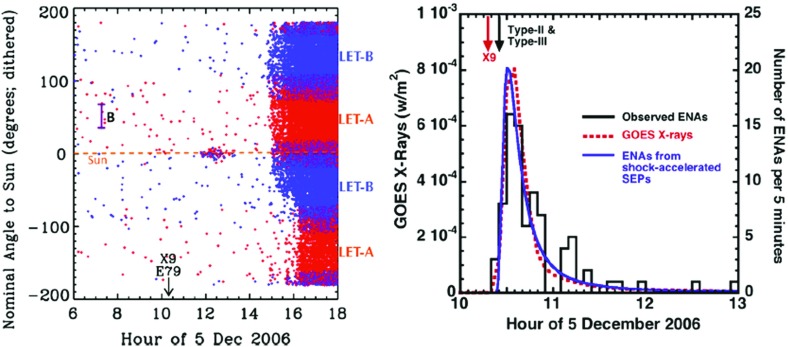
*Left* Measured angle with respect to the Sun-s/c line for individual 1.6–12 MeV protons observed on December 5, 2006 by the low energy telescopes (LET) on STEREO. *Red* $=$ STEREO A and *blue* $=$ STEREO B. A small group of events arrived from within ${\pm }10^{\circ }$ of the Sun-s/c line between $\sim$1130 UT to $\sim$1350 UT, i.e., well before the SEP onset at $\sim$1445 UT. The range of magnetic field orientations connecting to the Sun between 1130 and 1350 UT is shown as a *bar*. *Right* Comparison between the timing of associated solar events and ENA arrival at STEREO (adapted from Mewaldt et al. 2009)

### New insights using energetic neutral atoms

In association with an X9 flare on December 5, 2006, at E79, i.e., when Earth and the recently launched twin STEREO s/c were magnetically poorly connected to the Sun, the earliest ${>}30\,\mathrm{MeV}$ protons arrived at the two s/c at $\sim$1445 UT (see Fig. 25). However, both of the low-energy-telescopes (LET) on board STEREO A and B detected a lower energy signal of ${\sim }2$–$12\,\mathrm{MeV}$ “protons” from $\sim$1130–1350 UT that arrived within ${\pm }10^{\circ }$ of the Sun-s/c line. Since it is impossible for $\sim$2–12 protons to travel from the Sun and arrive at Earth-orbit (i.e., distance of at least ${\sim }1\,\mathrm{AU}$) within the first hour of the corresponding solar event (flare or CME shock), Mewaldt et al. (2009) concluded that this precursor signal must have consisted of energetic neutral atoms (ENAs) of hydrogen that were most likely produced by CME-shock-accelerated protons as the shock moved from ${\sim }2$ to $20\,R_{S}$. This model (simulations in blue in Fig. 25b) can explain both the ENA fluence and emission time profile from the Sun. Note that the ENA emission profile is also consistent with the GOES 1–8 Å X-ray time profile, raising the possibility that the ENAs could also have been created by charge exchange of flare-accelerated particles. However, based on estimates of the number of flare-accelerated protons from RHESSI $\gamma$-ray observations, the flare-origin scenario requires that a significant fraction of these protons escape into the high corona, because otherwise the ENAs would have been stripped before leaving the Sun and would therefore have arrived much later with the SEP protons.

Thus, ENA imaging may provide a new tool to map SEP intensity distributions versus time and radius, and to compare with CME and radio data to enable more accurate “nowcasts” of near-Sun SEP intensities. Note that, for a typical SEP event, the flux of the initial higher energy SEPs would overwhelm the relatively smaller, lower energy ENA signal. Fortuitously, in the December 2006 event, the SEP event generated a detectable ENA signal event because the solar progenitor occurred near the east limb, which resulted in longer delays for the SEP onset. Future ENA detectors will need to develop techniques that can discriminate ENAs from SEPs to pursue this exciting research area.

## Energetic storm particle events

Transient IP shocks at ${\sim }1\,\mathrm{AU}$ are occasionally accompanied by enhancements in the intensities of energetic ions above ${\sim }0.05\,\mathrm{MeV/nucleon}$ (e.g., Scholer et al. 1983; Armstrong et al. 1985; Kennel et al. 1986; Reames 1999). It is well established that the majority of such IP shocks are driven by fast CMEs as they propagate through interplanetary space (see Gosling 1993), and the accompanying particle enhancements, historically known as ESP events because of their association with “Sudden Storm Commencements,” are energized via the DSA processes (e.g., Lee 1983; Reames 1999). Simultaneous in-situ measurements of the magnetic field and solar wind properties that can characterize IP shock properties, and their direct comparison with properties of the associated ESP events, yield crucial information about the source populations and the underlying acceleration mechanisms. This is primarily because the effects of transport through the interplanetary medium from remote acceleration sites near the Sun and in the inner heliosphere (e.g., flares and CME shocks) during ESP events are essentially negligible. This section presents some of the recent ESP observations that have significantly advanced our understanding of the physics of the source, injection, and DSA acceleration mechanisms at IP shocks.

### Temporal profiles

**Fig. 26:**
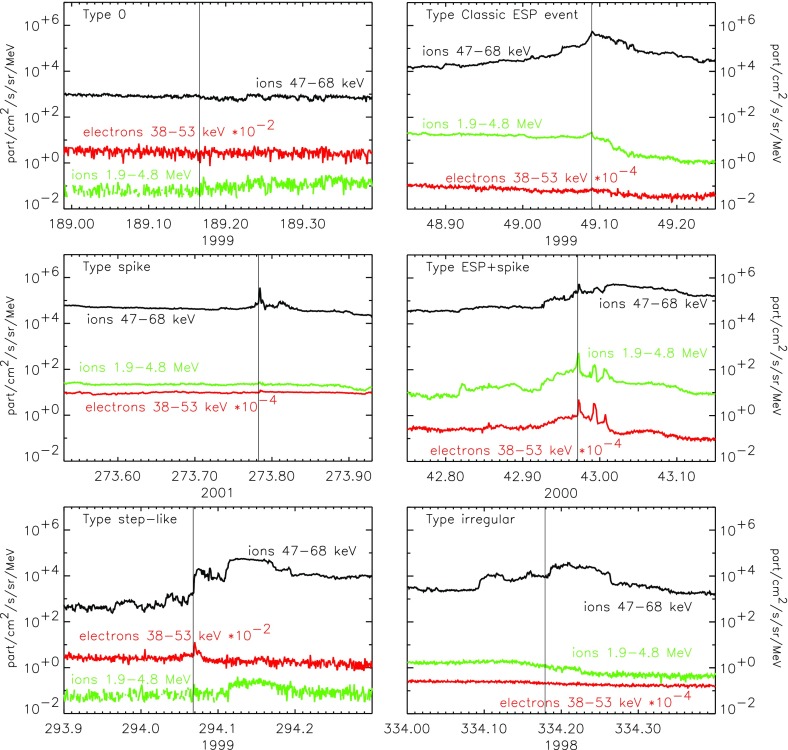
Examples of the variety of time-intensity profiles of ${\sim }47$–68 keV and ${\sim }1.9$–4.8 MeV ions and ${\sim }38$–53 keV electrons observed in IP shock-associated ESP events at ACE. Image reproduced with permission from Lario et al. (2003), copyright by AIP

Figure 26 (taken from Lario et al. 2003) shows six different categories of time histories of 47–68 keV and 1.9–4.8 MeV ions, and 38–53 keV electrons associated with IP shocks observed by the ACE/Electron, Proton, and Alpha Monitor (EPAM) at the L1 Langrangian point. These are: (1) Type 0: No obvious intensity variation above the pre-existing intensity level; (2) Type 1: Slow rise of the particle intensity beginning several hours before the shock (Classic ESP event); (3) Type 2: Intensity spike of a few (${\sim }10$) minutes duration at or near the shock (Spike); (4) Type 3: A classic ESP event with a spike at or near the shock superimposed on it (ESP $+$ spike); (5) Type 4: Step-like post-shock increase (Step-like); and (6) Type 5: Irregular time-intensity profile with flux variations not coincident with the shock passage and not fitting into the types described above (Irregular).

**Fig. 27:**
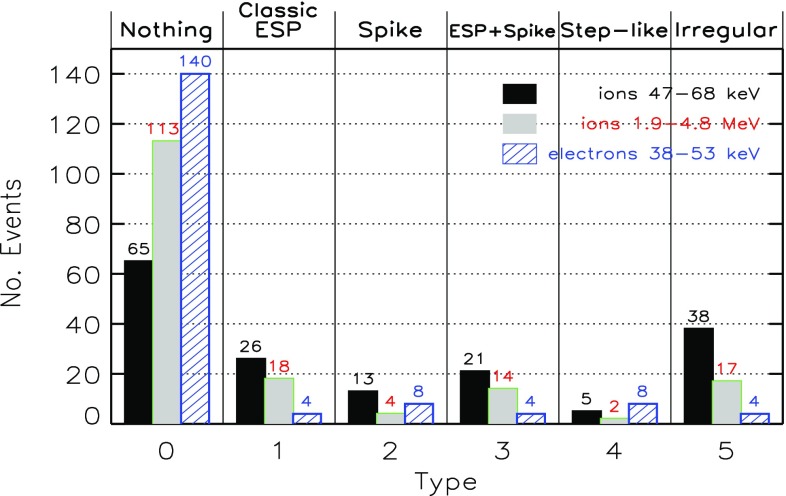
A histogram of the classification of time-intensity profiles of energetic protons and electrons associated with 168 interplanetary shocks observed by ACE from 1997–2001. Image reproduced with permission from Lario et al. (2003), copyright by AIP

**Fig. 28:**
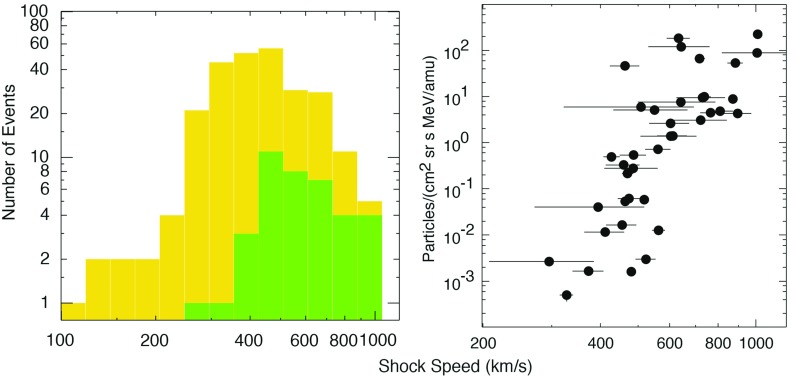
*Left* Distribution of IP shocks with measurable intensities of ${>}1\,\mathrm{MeV/nucleon} ^{4}$He ions versus shock speed (*green*) within the total distribution of 258 shock waves versus shock speed (*yellow* and *green*) observed by Wind. *Right* Scatter-plot of the background-corrected peak intensity of ${\sim }1.6$–$2.0\,\mathrm{MeV/nucleon}$ $^{4}$He nuclei versus shock speed. Image reproduced with permission from Reames (2013), copyright by Springer

Lario et al. (2003) classified the time-intensity profiles associated with over 150 IP shock events into one of the six categories described above, as shown in Fig. 27. The category “classic ESP” refers to the prediction of DSA of an exponential rise to the shock, followed by a nearly constant downstream intensity. The most striking feature of this figure is that the most common of the categories is that there was *no* change in the energetic particle intensity. The Lario et al. (2003) study showed that approximately 40 % of the IP shocks observed near Earth orbit were not accompanied by ion intensity enhancements above ${\sim }50\,\mathrm{keV}$ energy, and that the observed complexity must be taken into account while comparing or testing particle observations with locally measured shock parameters. Schwadron et al. (1996) performed a similar study of interstellar pickup ions associated with corotating interaction regions (CIRs), and arrived at a similar conclusion. Similarly, Reames (2012) found that only 39 of the 258 IP shocks observed at Wind were associated with ${\sim }10\,\mathrm{MeV/nucleon}$ He intensity increases (see Fig. 28) and, similarly, Cohen et al. (2005a) found that only 57 of 354 shocks at ACE were associated with ${\sim }10\,\mathrm{MeV}$ proton intensity increases. Thus, it appears that the association between ESP events and IP shocks decreases substantially with increasing energy.

Giacalone (2012) analyzed ${\sim }50\,\mathrm{keV}$ proton intensities and energy spectra at 19 IP shocks with Alfvén Mach numbers >3 and plasma density jump >2.5; they found that 18 of these strong IP shocks were also associated with intensity enhancements of ions with energies between ${\sim }50$ and $300\,\mathrm{keV}$. The time-intensity profiles in these 18 events were quite similar to the predictions of DSA theory (i.e., would fall into the “Classic ESP” category of Fig. 27). One way to understand these results is that the much larger IP shock-event list studied by Lario et al. (2003), contains many weak shocks that are perhaps not capable of accelerating particles locally. Thus, the fraction of shocks that have concomitant energetic particle enhancements is likely related to the strength of the shock, to the general issue of the seed particles on which the shock acceleration mechanisms operate (Desai et al. 2006a), and to the effects of turbulence (e.g., Neugebauer et al. 2006; Giacalone and Neugebauer 2008).

**Fig. 29:**
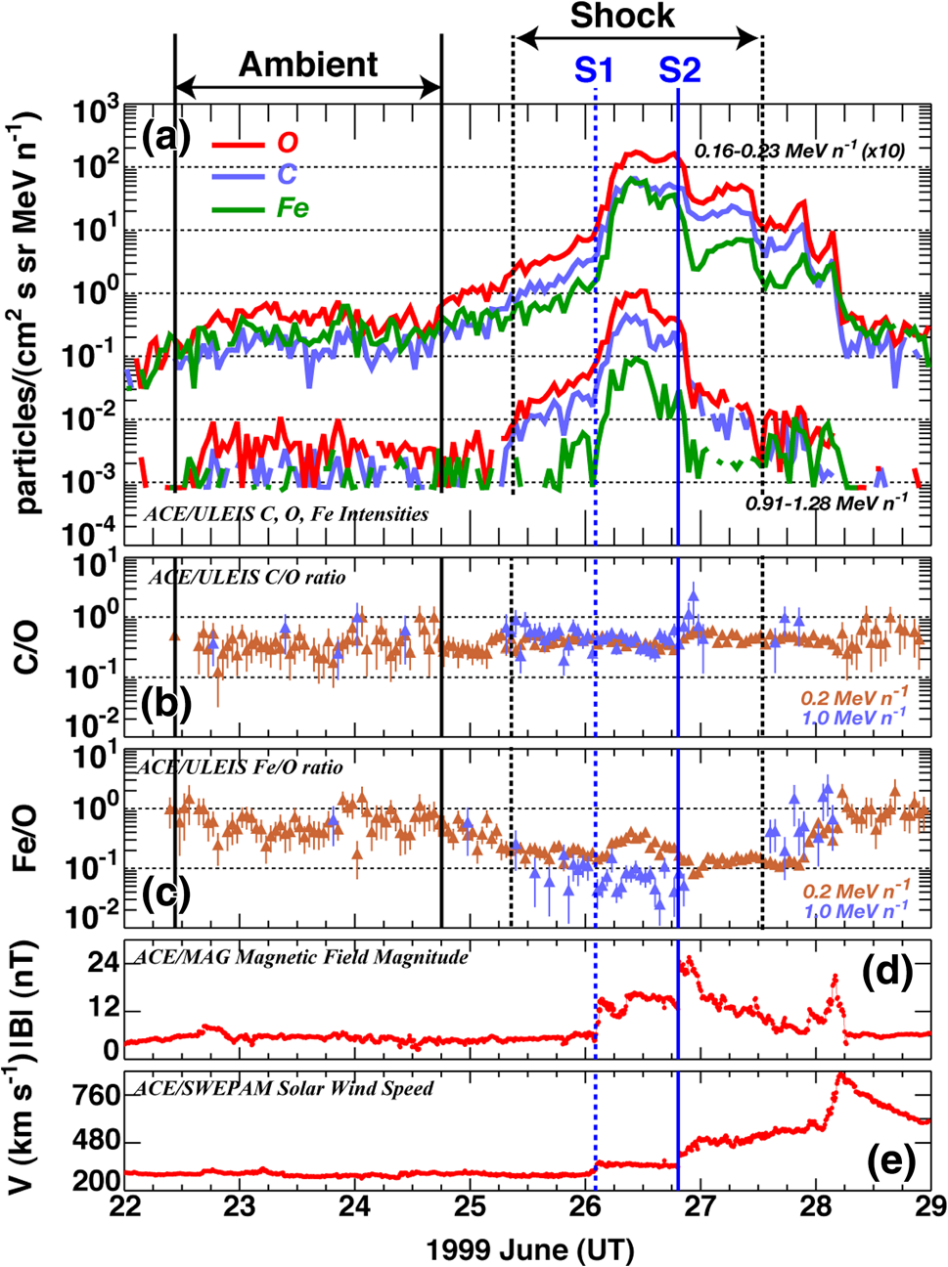
Hourly averages of *a* ${\sim }0.16$–0.23 and ${\sim }0.91$–$1.28\,\mathrm{MeV/nucleon}$ C, O, and Fe intensities; *b* C/O ratios; and *c* Fe/O ratios from June 22–29, 1999, measured by ACE/ULEIS. The IP shock is identified using 5 min averages of *d* the magnetic field magnitude *B*, and *e* the SW speed *V*. *Blue vertical lines* marked S1 and S2 denote the IP shock arrival times at ACE. *Dashed black vertical lines* represent the time interval for measuring shock-associated energetic ions. *Solid black vertical lines* represent the time interval for measuring ambient energetic ions in the interplanetary medium. Image reproduced with permission from Desai et al. (2004), copyright by AAS

Figure 29 shows the temporal behavior of heavy ions C, O, and Fe at two different energies during an IP shock event observed at ACE. Contrasting the behavior of the C/O ratio with that of the Fe/O ratio at the two energies near shock passage: the C/O ratio at both energies remains essentially constant, while the Fe/O ratio at both energies shows dramatic decreases that coincide with the increases in intensities at corresponding energies. Further, the Fe/O ratio at ${\sim }1\,\mathrm{MeV/nucleon}$ decreases even more when compared with the corresponding decrease at ${\sim }0.2\,\mathrm{MeV/nucleon}$. The decrease in the Fe/O ratio is typical for strong IP shocks and is observed at all energies up to ${\sim }100\,\mathrm{MeV/nucleon}$ (e.g., Desai et al. 2004).

### ESP properties, CME and IP shock properties

Reames (2012) investigated the influence of various shock parameters on the properties of accelerated particle populations. Figure 28 (left panel) shows a histogram of the shock speed distribution for all IP shocks (yellow) observed by Wind and for the subset (green) that showed measurable signatures of particle acceleration, i.e., intensity increases in the $\sim$1–10 MeV/nucleon He ions. This survey found that the strongest particle acceleration effects occurred for IP shocks with high shock speed, high shock compression ratio, and highly oblique shock-normal angles ($\theta _{Bn}$), in decreasing order of importance. Specifically, Reames (2012) also found that quasi-perpendicular IP shocks with $\theta _{Bn}{>}60^{\circ }$ were more likely than quasi-parallel shocks with $\theta _{Bn}{<}30^{\circ }$ to be associated with larger effects of particle acceleration. This is consistent with predictions from one application of DSA theory—that highly oblique shocks have a higher acceleration rate compared to shocks that move nearly along the field (e.g., Jokipii 1987; Giacalone 2005a). However, this result is at odds with other DSA theoretical models and numerical calculations by Lee (2005) and Zank et al. (2006), which predict that protons accelerated by quasi-parallel shocks stream away from the shock and generate self-excited Alfvén waves, which makes such shocks highly efficient at trapping and accelerating particles. The dependence of the particle intensity on the shock speed also follows naturally from DSA theory and is discussed further in Sect. 7.2.3.

Figure 28 (right panel) shows that the pre-event, background-subtracted ${\sim }1.6$–$2.0\,\mathrm{MeV/nucleon}$ peak $^{4}$He intensity correlates with the IP shock speed, with a correlation coefficient of ${\sim }0.8$. Although this correlation is statistically significant, the nearly two orders of magnitude in peak intensity variations over a limited range (${\sim }500$–$1000\,\mathrm{km/s}$) of shock speeds pose significant constraints on theories and models used to explain and predict ESP properties at Earth-orbit.

### Spectral properties of ESP events

**Fig. 30:**
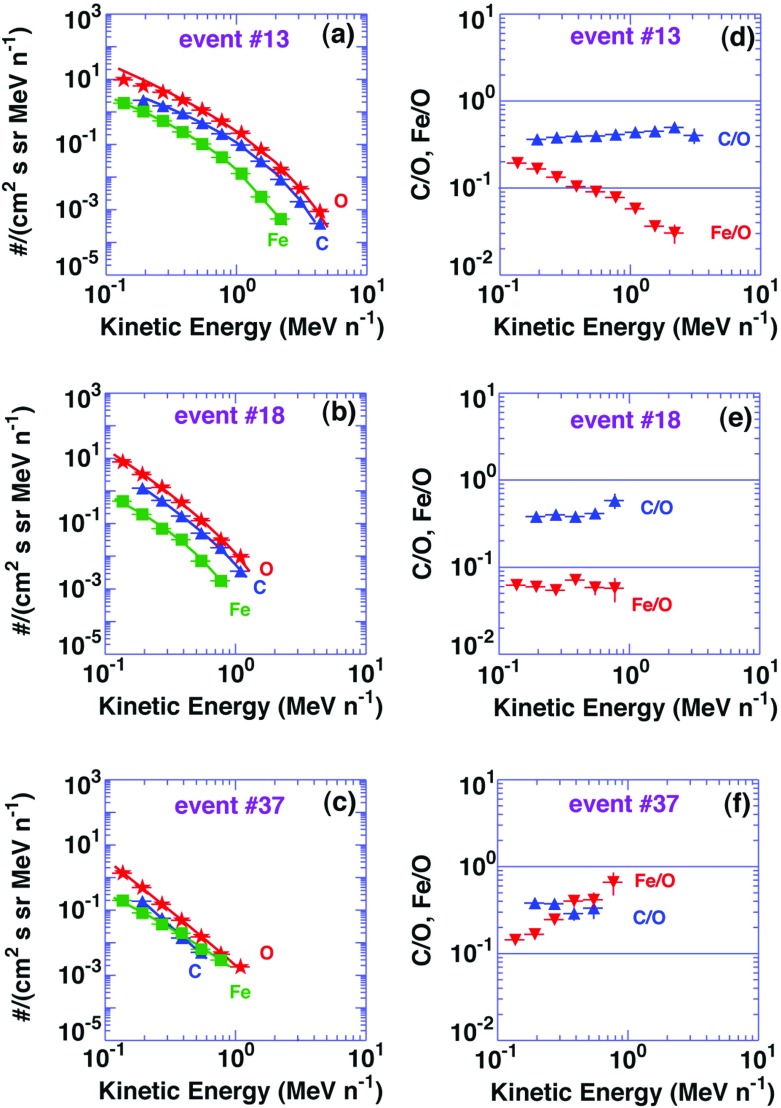
*Left* Energy spectra of C, O, and Fe during three ESP events. The *solid curves* show fits with the Jones and Ellison expression, where the differential intensity is given by $J={ CE}^{-\gamma } \exp (-E/E_{0}$). *Right* C/O and Fe/O ratios versus energy for the three IP shock events. Image reproduced with permission from Desai et al. (2004), copyright by AAS

Figure 30 shows the C, O, and Fe spectra (left panels) and the energy-dependence of C/O and Fe/O ratio during three ESP and IP shock events from solar cycle 23; top panels shows the spectra and ratios for the June 26, 1999 event shown in Fig. 29 (taken from Desai et al. 2004). Out of 33 ESP events that extended to ${\sim }2\,\mathrm{MeV/nucleon}$, the Fe/O ratio decreased with increasing energy in 19 events, remained constant in 12 events, and increased with energy in two events.

The spectra in the left-hand panels are fitted by the expression $J={ CE}^{-\gamma } \exp (-E/E_{0})$, where *J* is the differential intensity, *E* is the particle energy in MeV/nucleon, $\gamma$ is the spectral index, and $E_{0}$ is the e-folding energy (Jones and Ellison 1991). The values of $E_{0}$ for C, O, and Fe during the June 26, 1999 event (top panels) are ${\sim }0.79\,\pm \,0.06, {\sim }1.0\,\pm \,0.12$, and ${\sim }0.48\,\pm \,0.03$; i.e., the value of $E_{0}$ for Fe is lower by about a factor of 2 than those for C and O, leading to the dramatic decrease in the Fe/O ratio with increasing energy seen in Fig. 30d. The values of the spectral indices and $E_{0}$ for the event shown in Fig. 30b, e are similar for all three species, while the heavy ion energy spectra for the event shown in Fig. 30c, f can be fitted by pure power laws.

Desai et al. (2004) interpreted the behavior seen in Fig. 30a, d as evidence of an M/Q-dependent acceleration mechanism where ions with larger M/Q ratios like Fe are accelerated less efficiently than those with lower M/Q ratios like C and O. IP shock events that extended above ${\sim }10\,\mathrm{MeV/nucleon}$ also exhibited Fe/O ratios that decreased with increasing energy (Desai et al. 2004). The behavior seen in the other two events was interpreted in terms of re-acceleration of pre-existing seed populations that exhibit similar spectral properties, e.g., the increase in Fe/O with increasing energy seen in Fig. 30c, f is attributed to a suprathermal-through-energetic ion seed population that also has an increasing Fe/O ratio with increasing energy.

**Fig. 31:**
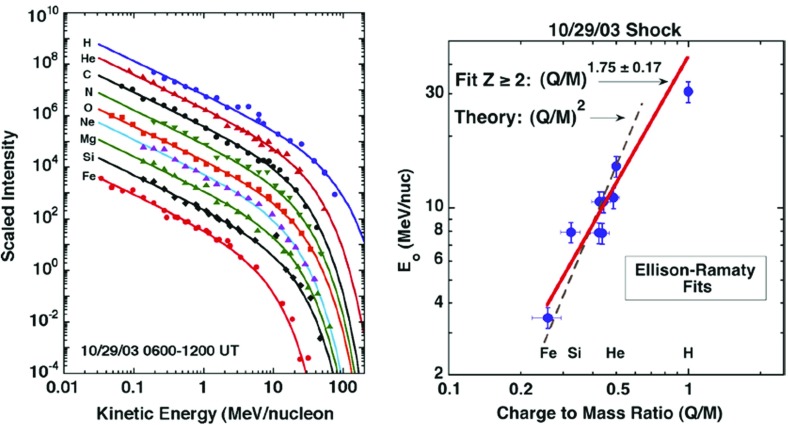
*Left* Heavy ion energy spectra from ACE and GOES following an IP shock on October 29, 2003, are fitted with the Ellison and Ramaty (1985) spectral form for differential intensity, $J={ CE}^{-\gamma } \exp (-E/E_{0}$). All elements are fitted with power-laws of the same spectral index: $\gamma = 1.3$ (Mewaldt et al. 2005c). Spectra of different elements are scaled for clarity. *Right* Values of the e-folding energies $E_{0}$ versus the ion’s Q/M ratio. Fits to the values of $E_{0}$ for $\mathrm{Z}\ge 2$ give a (Q/M)$^{1.75}$ dependence, which is somewhat weaker than that predicted by Li and Zank (2005)

Mewaldt et al. (2005a, (2005c) fitted the energy spectra of many heavy ion species during the October 29, 2003 IP shock event with a constant spectral index $\gamma =1.3$ and explored the M/Q-dependence of the roll-over or break energy $E_{0}$, as shown in Fig. 31. Mewaldt et al. (2005c) suggested that this behavior can be explained if the heavy-ion spectra roll over at the same value of the diffusion coefficient (see also Tylka et al. 2000; Cohen et al. 2005b). In this case, if the diffusion coefficients of different ion species depend on particle rigidity or M/Q, then Fe, with its higher M/Q ratio or rigidity, will roll-over at lower energy/nucleon. The observed Q/M-dependence of the e-folding energies scales as $(Q/M)^s$, with $s\approx 1.75$, which is close to the value of $s\approx 2$, as predicted by Li and Zank (2005) for quasi-parallel shocks. Li et al. (2009) later developed a more general model for the Q/M-dependence for shocks with different $\theta _{Bn}$ and showed that *s* could range from $\approx 0.2$ for quasi-perpendicular shocks to $\approx 2$ for quasi-parallel shocks.

Early theoretical studies based on DSA theory (e.g., Lee 1983) successfully predicted many features of ESP events associated with some IP shocks near 1 AU (e.g., Kennel et al. 1986). However, such detailed agreements between theory and observations are extremely rare (see also Lario et al. 2005). In contrast, studies involving a large number of IP shocks have shown that the predicted spectral indices for energetic protons and heavy ions near ${\sim }100\,\mathrm{keV/nucleon}$ are significantly different from the observations (van Nes et al. 1984; Desai et al. 2004). Giacalone (2012) showed that a better agreement between theory and observations is obtained when considering only strong shocks, but the uncertainties are still significant.

**Fig. 32:**
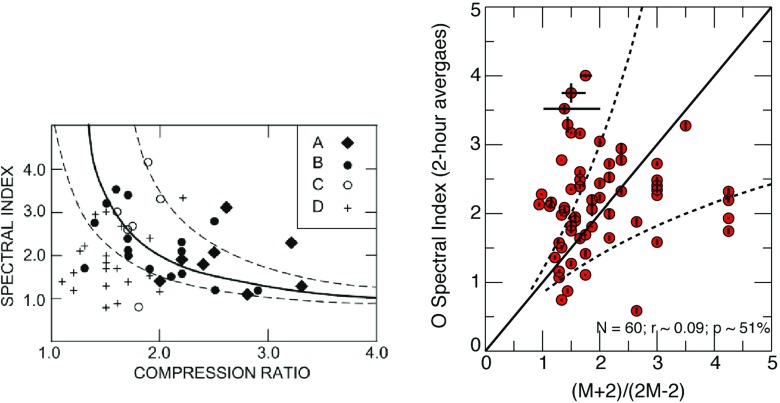
*Left* Shock compression ratio versus spectral index $\gamma$ of $\sim$30–50 keV energy ions in 50 ESP events (adapted from van Nes et al. 1984). The *symbols* denote ESP events with four different types of time-intensity profiles (see text for details). *Right* Scatter plot of the 0.1–0.5 MeV/nucleon O spectral indices versus $(M+2)/(2M-2)$ for 60 ESP events; here *M* is the magnetic compression ratio (adapted from Desai et al. 2004). The *solid curve* represents $\gamma =(M+2)/(2M-2)$, as expected from diffusive shock acceleration theory; the *dashed curves* arise from ${\pm }25\,\%$ uncertainty in the compression ratio (*left*) and *M* (*right*). *N* is the number of events, *r* is the correlation coefficient, and *p* is the probability that the value of the correlation coefficient can be exceeded by a pair of uncorrelated parameters

Desai et al. (2004) studied the energy spectra of ${\sim }0.1$–$100\,\mathrm{MeV/nucleon}$ heavy ions during 72 CME-driven IP shock events surveyed by Desai et al. (2003). They found that the power-law spectral indices for 0.1–0.5 MeV/nucleon O ions were poorly correlated with the values predicted by simple one-dimensional steady-state models, as shown in Fig. 32. Likewise, poor agreement between the theoretical predictions and the low-energy (${\le }0.5\,\mathrm{MeV}$) proton spectral indices were also found earlier by van Nes et al. (1984), and more recently by Ho et al. (2009). Desai et al. (2004) also found that the characteristic e-folding or roll-over energy of the O spectra at IP shocks was uncorrelated with shock parameters such as shock normal angle or shock speed.

**Fig. 33:**
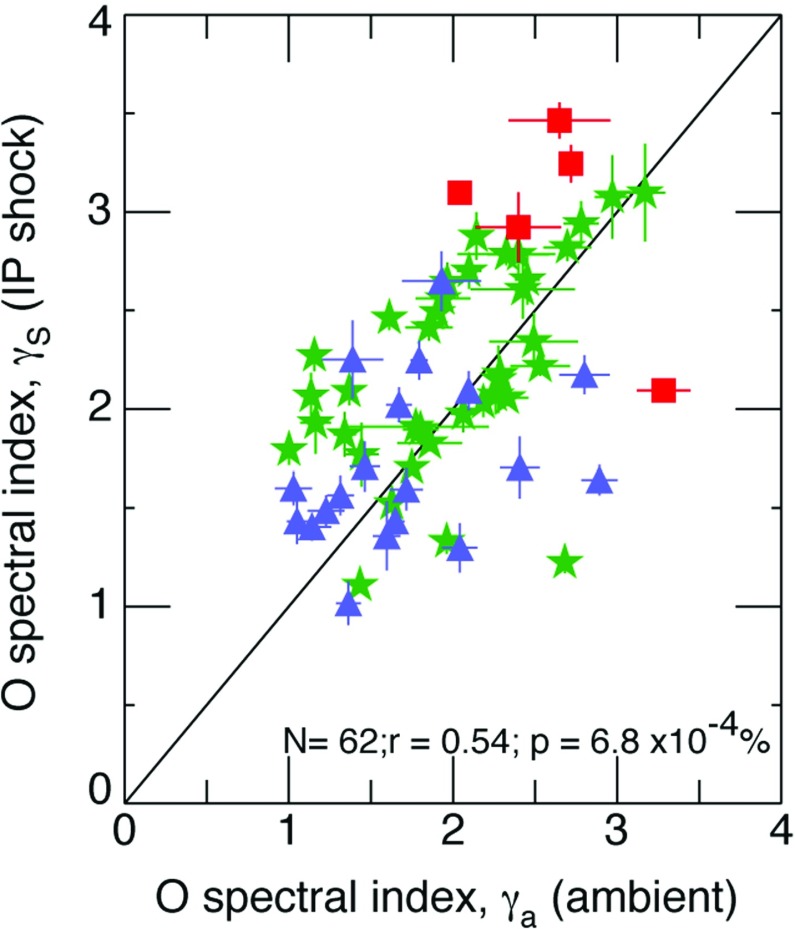
O spectral index at IP shocks versus that for the ambient suprathermals. Image reproduced with permission from Desai et al. (2004), copyright by AAS

In contrast, Fig. 33 shows that the O spectral index in IP shocks is well correlated with the corresponding quantities measured in the ambient population. Such strong correspondence between the accelerated ions and the ambient suprathermal ions is not predicted by simple DSA theory. We remark that Fig. 2 of Reames (2012) shows similar results for ${\sim }1$–$10\,\mathrm{MeV/nucleon}$ He intensities observed at 39 IP shocks. Specifically, Reames (2012) showed that the observed spectral indices are poorly correlated with those expected from DSA theory, in which particles are injected out of a fixed low-energy thermal seed population. In contrast, these indices are well correlated with the upstream or background spectral index. Based on theoretical considerations of re-accelerating a power-law seed spectrum at shocks (e.g., Axford 1982; Melrose and Pope 1993; Lee 2008), Reames (2013) discussed and emphasized the importance of how a harder background or seed population spectrum is observed or preserved at weaker shocks for which the shock compression ratios should yield steeper spectra. These models also predict that the power-law indices at stronger shocks reflect the shock compression ratios (also see Channok et al. 2005).

### $^{3}$He and $\hbox {He}^{+}$ ions in IP shocks

Like SEPs, the origin of the source populations of ESPs also remains an unsolved problem. Some researchers have proposed that ESP protons result from the acceleration of thermal solar wind (e.g., Lee 1983; Baring et al. 1997), while others point out that the suprathermal tail of the solar wind may be the source (e.g., Gosling et al. 1981; Tsurutani and Lin 1985; Tan et al. 1989). Tsurutani and Lin (1985) and Tan et al. (1989) suggest that the concomitant solar flares might provide the suprathermal seed particles accelerated at the IP shocks.

**Fig. 34:**
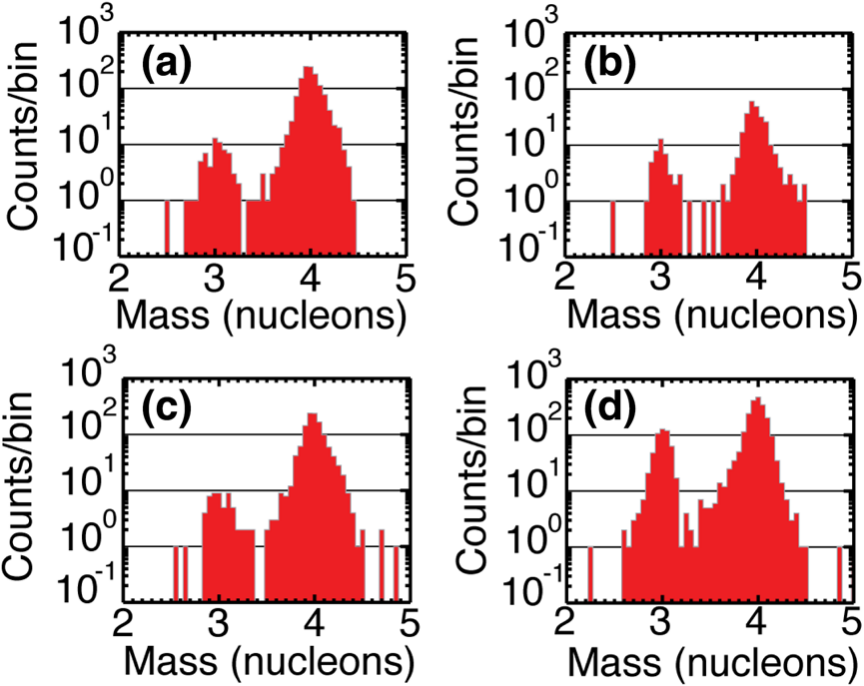
The $\sim$0.5–2.0 MeV/nucleon mass histograms during four $^{3}$He-enriched IP shock events at ACE. Image reproduced with permission from Desai et al. (2001), copyright by AAS

Since these earlier studies, instruments with greater sensitivity and resolution on board ACE (Stone et al. 1998) have provided major observational advances in terms of measuring the solar wind ion composition and its variations (e.g., von Steiger et al. 2000) and comparing them with the energy-dependence and event-to-event variability of the ionic charge state, and elemental and isotopic composition in ESP and SEP events over a broad energy range (e.g., Oetliker et al. 1997; Mazur et al. 1999; Möbius et al. 1999; Cohen et al. 2005b; Desai et al. 2006a; Klecker et al. 2007). These new observations have made it possible to re-examine questions about the origin of the seed populations and probe details of the acceleration mechanisms in individual ESP events. In the remainder of this section, we discuss key observations that have spawned a re-evaluation of the origin of the seed populations, and of the role of self-excited waves during ESP events.

Using measurements from ACE/ULEIS (Mason et al. 1998), Desai et al. (2001) surveyed 48 IP shocks between October 1997 and November 2000. The results showed upper limits of $^{3}$He in 23 events, while the remaining 25 events had substantial enhancements in $^{3}$He abundance that ranged between factors of $\sim$2–600 times the corresponding solar wind value. Figure 34 shows the 0.5–2.0 MeV/nucleon He mass histograms in four events where the $^{3}$He/$^{4}$He ratio was a hundred times greater than that measured in the solar wind. Such $^{3}$He enrichments are routinely present during the smaller flare-related events that are more frequent during periods of high solar activity. These solar flares populate and replenish the interplanetary medium with suprathermal $^{3}$He-rich material, which subsequently gets re-accelerated by the CME-driven IP shocks whenever they encounter it en route to Earth (Mason et al. 1999). ACE results also showed a good correspondence between the occurrence frequency of $^{3}$He-rich IP shocks and the fraction of time that suprathermal $^{3}$He is present at 1 AU (Wiedenbeck et al. 2003), which provides further support for a suprathermal origin for the seed population.

**Fig. 35:**
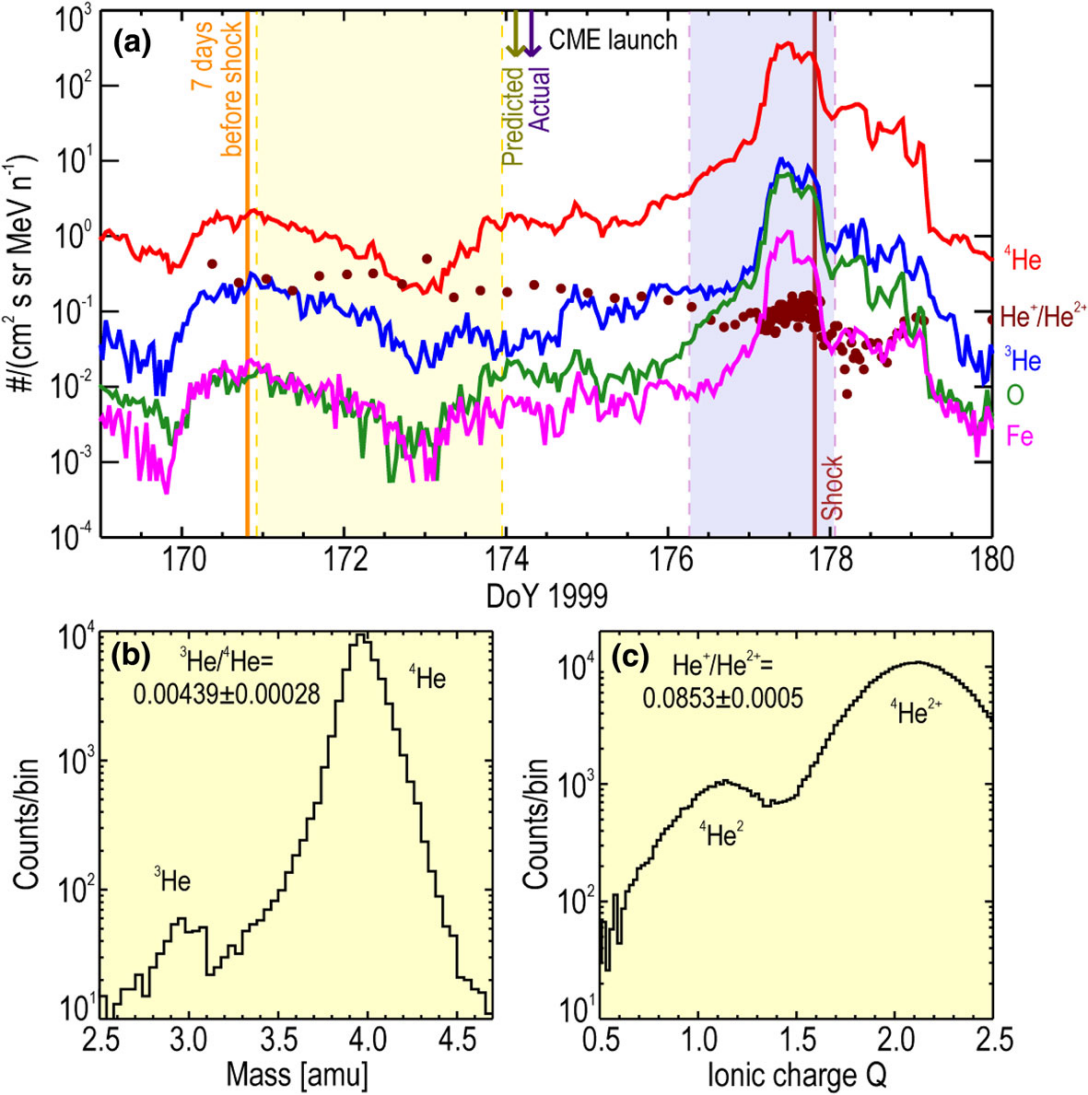
**a** Hourly averaged intensity profiles of $\sim$0.25–0.8 MeV/nucleon $^{3}$He, $^{4}$He, O, and Fe (*solid lines*) measured by ACE/ULEIS and the $\hbox {He}^{+}$/He$^{2+}$ ratio (*filled circles*) measured by ACE/SEPICA during an IP shock event. The *yellow-shaded region* identifies the ambient interval, the *purple-shaded region* shows the time interval for sampling the shock-associated energetic particles, the *brown line* denotes the shock arrival time at ACE, and *arrows at the top* indicate the estimated and actual CME lift-off times near the Sun. **b** He mass histogram from ULEIS, and **c** He charge state histogram from SEPICA showing well-resolved He$^{2+}$ and $\hbox {He}^{+}$ peaks during the ESP event (adapted from Allegrini et al. 2008)

Similarly, $\hbox {He}^{+}$ ions act as tracers of the interstellar neutral gas that flows unimpeded into the inner solar system (Möbius et al. 1985). Neutral He gets ionized inside 1 AU and is subsequently picked up by the out-flowing solar wind (Gloeckler et al. 1993). Near 1 AU, these ions are present in the suprathermal tail with relative abundances of more than $10^{3}$ times the corresponding solar wind value. Figure 35 shows the $\hbox {He}^{+}$/He$^{2+}$ ratio at $\sim$0.5 MeV/nucleon during a CME-driven IP shock event (from Allegrini et al. 2008). The He ionic charge state histogram is obtained by the Solar Energetic Particle Ionic Composition Analyzer (SEPICA: Möbius et al. 1998), and shows a clear enhancement in the $\hbox {He}^{+}$ abundance, well above that measured in the solar wind. Kucharek et al. (2003) also compared the time histories of ${\sim }0.2$–$0.8\,\mathrm{MeV/nucleon} \hbox {He}^{+}$/He$^{2+}$ ratios measured in several IP-shock events with those measured in the thermal SW surrounding the event. The SW $\hbox {He}^{+}$/He$^{2+}$ is obtained by the Solar Wind Electron Proton and Alpha Monitor (SWEPAM: McComas et al. 1998) on board ACE. Kucharek et al. found that large enhancements in the energetic $\hbox {He}^{+}$ abundance at two IP shocks did not coincide with the ones observed earlier in the thermal plasma. Conversely, Kucharek et al. studied the case of one “cloud” in which thermal $\hbox {He}^{+}$ ions were relatively more abundant, but the IP shock that followed it did not appear to accelerate as many $\hbox {He}^{+}$ ions as the two IP shocks discussed above.

The presence of energetic $^{3}$He and $\hbox {He}^{+}$, which are very rare in the solar wind, suggests that these species were accelerated from a population of pre-existing suprathermal ions, rather than from the bulk solar wind. Near $\sim$ twice the bulk SW speed region, pickup ion distributions are more prominent compared with SW alpha particles (Gloeckler et al. 1994). This taken together with substantial $\hbox {He}^{+}$ enrichments in the accelerated populations suggest that the DSA injection process may also be substantially more efficient near and above this energy range (also see Chotoo et al. 2000; Mason 2000).

### Heavy ion composition in ESP events

**Fig. 36:**
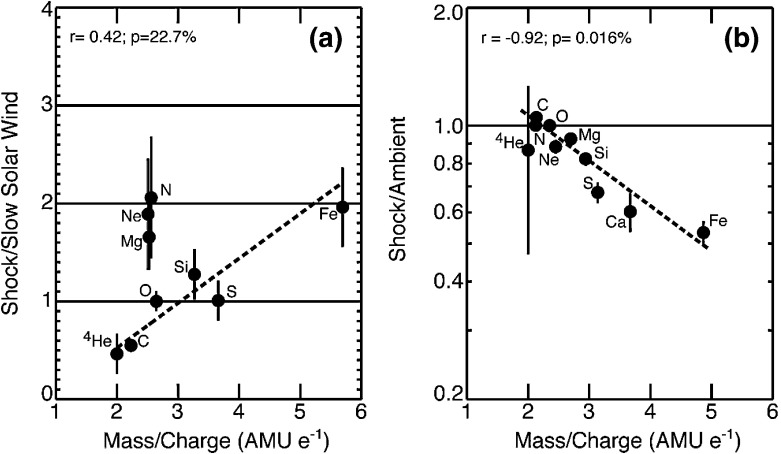
Mean abundances in 72 IP shocks normalized to: **a** slow SW values, and **b** mean abundances measured upstream of the shocks, plotted versus the ion’s M/Q ratio (adapted from Desai et al. 2003)

ACE measurements have also allowed us to investigate whether the more common heavier ions such as C–Fe originate from the bulk solar wind or from the suprathermal tail. Desai et al. (2003) compared the average ${\sim }1\,\mathrm{MeV/nucleon}$ ion abundances in 72 IP shocks at ACE with those measured in the solar wind (from von Steiger et al. 2000) and other candidate seed populations. Figure 36a shows the average IP shock abundances normalized to the slow solar wind values and plotted versus M/Q. The ionization states used here are typical of those measured in the slow solar wind (von Steiger et al. 1997). Note that the C/O ratio in IP shocks is about a factor of 2 lower than that measured in the solar wind. Since most known shock acceleration mechanisms (e.g., Lee 1983) fractionate ion species according to their M/Q ratios, such large differences in the abundances of accelerated and solar wind material cannot be primarily attributed to injection and acceleration of a seed population dominated by solar wind material. The unsystematic behavior of other heavier nuclei such as N, O, Ne, and Mg with similar M/Q ratios results in a poor correlation with the corresponding solar wind abundances. These results provide additional evidence that the ESP heavy ion population probably does not originate from the solar wind but rather from a pre-accelerated suprathermal pool.

In contrast, Fig. 36b shows that the IP shock abundances were well correlated with the average abundances measured at the same energy (${\sim }1\,\mathrm{MeV/nucleon}$) in the interplanetary medium prior to the arrival of the IP shocks at ACE (Desai et al. 2003). In particular, elements with higher M/Q ratios are systematically depleted, which is consistent with shock acceleration models wherein ions with higher M/Q ratios are accelerated less efficiently than those with lower M/Q values (e.g., Lee 2005). In addition to the correlations between the average quantities, Desai et al. (2003) found significant correlations between the IP shock abundances (e.g., ${\sim }1\,\mathrm{MeV}$/nuc Fe/O ratio) and those measured in the ambient suprathermal ions for individual events. Since the ambient heavy ion population contained $\sim$30 % of material from flare-related SEP events and the remainder from large CME-related SEP events, these results indicate that the heavy ions from C–Fe also originate from a suprathermal tail that is essentially dominated by ions accelerated by prior IP shocks (Desai et al. 2003).

### Role of self-excited waves

Particles can gain energy at shocks either via the first-order Fermi mechanism by being scattered between magnetic inhomogeneities or waves that are convected by converging flows on either side of the shock, or via the shock-drift mechanism by drifting along the shock front parallel to the $\mathbf{V} \times \mathbf{B}$ electric field (e.g., Jokipii 1982; Lee 1983; Decker 1988). Although these mechanisms have been studied extensively and incorporated within the framework of DSA theory (see Sect. 7.2.4), the identity of the seed particles, the manner in which they are injected into the acceleration processes, and the mechanisms that limit these processes have remained controversial (e.g., Eichler 1981; Lee and Fisk 1982; Forman and Webb 1985; Jokipii 1987; Jones and Ellison 1991).

**Fig. 37:**
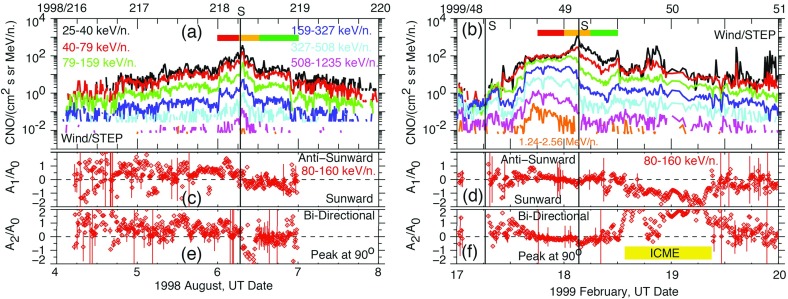
Wind/STEP C $+$ N $+$ O observations during the August 6, 1998 (*left*) and the February 18, 1999 (*right*) IP shock events. Each figure shows 10-min averages of *a*, *b* ${\sim }0.025$–$2.56\,\mathrm{MeV/nucleon}$ CNO intensities; *c*, *d* first-order $A_{1}/A_{0}$; and *e*, *f* second-order $A_{2}/A_{0}$, anisotropy components for ${\sim }80$–$160\,\mathrm{keV/nucleon}$ CNO ions in the solar wind frame. *Vertical lines* marked S indicate the IP shock arrival times at 1 AU. The *yellow bar* in (*f*) is the ICME interval. Image reproduced with permission from Desai et al. (2012), copyright by AIP

In one such DSA mechanism, the first accelerated protons stream along the upstream magnetic field away from quasi-parallel shocks and excite Alfvén waves, which then trap, scatter, and accelerate the subsequently accelerated ions with increased efficiency (e.g., Lee 2005). This coupling between the self-excited waves and the accelerated ions is often invoked to account for many ESP and SEP phenomena. However, during the last three decades of observations, clear signatures of wave excitation (Lee 1983), or first-order Fermi acceleration (Jokipii 1982), or shock-drift (Decker 1988) acceleration processes have been identified in only a handful of ESP events (Sanderson et al. 1985; Kennel et al. 1986; Gordon et al. 1999; Bamert et al. 2004; Lario et al. 2005; Smith et al. 2008). More recently, Desai et al. (2012) reported two case studies with clear signatures of wave excitation and shock-drift acceleration; here the intensity, energy spectra, and anisotropies were interpreted as being consistent with predictions of DSA theory. Figure 37 shows the energetic ion properties measured by the SupraThermal through Energetic Particle Telescope (STEP) on board Wind during two IP shocks that were observed at: (1) ${\sim }0716$ UT on August 6, 1998: $\theta _{Bn}\sim 83^{\circ } \,\pm \, 3^{\circ }$; and (2) ${\sim }0248$ UT on February 18, 1999: $\theta _{Bn}{\sim }55^{\circ }\,\pm \,2^{\circ }$.

**Fig. 38:**
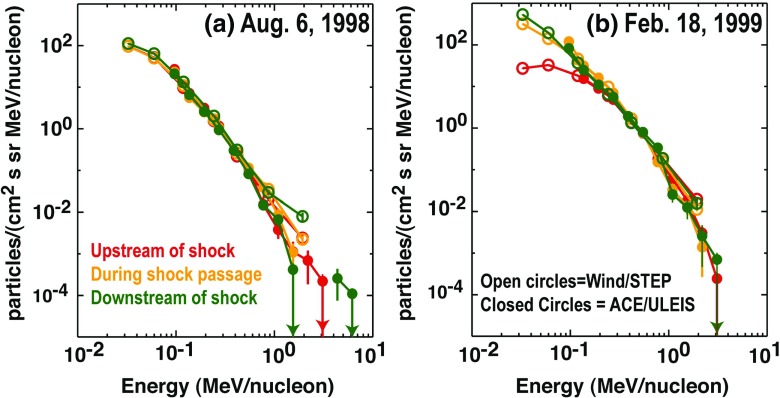
Temporal evolution of the energy spectra of CNO from Wind/STEP and O from ACE/ULEIS measured during three separate intervals identified by the *color-coded horizontal bars* shown in the two panels of Fig. 37, normalized to the intensity at ${\sim }300\,\mathrm{keV/nucleon}$

Figure 37 shows that the CNO intensities between $\sim$0.25 and 1.2 MeV/nucleon increase by about a factor of 10 during ${\sim }6$-h intervals on either side of the August 6, 1998 shock. In the case of the February 18, 1999 event, the intensities at all energies increase ${\sim }14$ h prior to the arrival of the shock, but only the $\sim$25–80 keV/nucleon CNO intensities peak near the shock. For the August 6, 1998 event, $A_{1}/A_{0}$ shows a large anti-sunward flow upstream of the shock, a flow reversal at the shock, and a sunward flow downstream. Note that, $A_{2}/A_{0}$ has a large negative component during a $\sim$4 h period downstream of the shock, indicating that the pitch-angle distributions peak at $90^{\circ }$ to the local interplanetary magnetic field (IMF) direction, consistent with previous observations and theoretical predictions of the shock-drift mechanism (e.g., Decker 1988). For the February 18, 1999 event: $A_{1}/A_{0}$ is close to zero for ${\sim }6$ h on either side of the shock; $A_{2}/A_{0}$ is close to zero from $\sim$1200 UT on February 17, 1999 to $\sim$1200 UT on February 18, 1999, then exhibits large positive values until $\sim$1200 UT on February 19, 1999, indicating the presence of bi-directional ion flows inside an ICME event (see Lario et al. 2004).

**Fig. 39:**
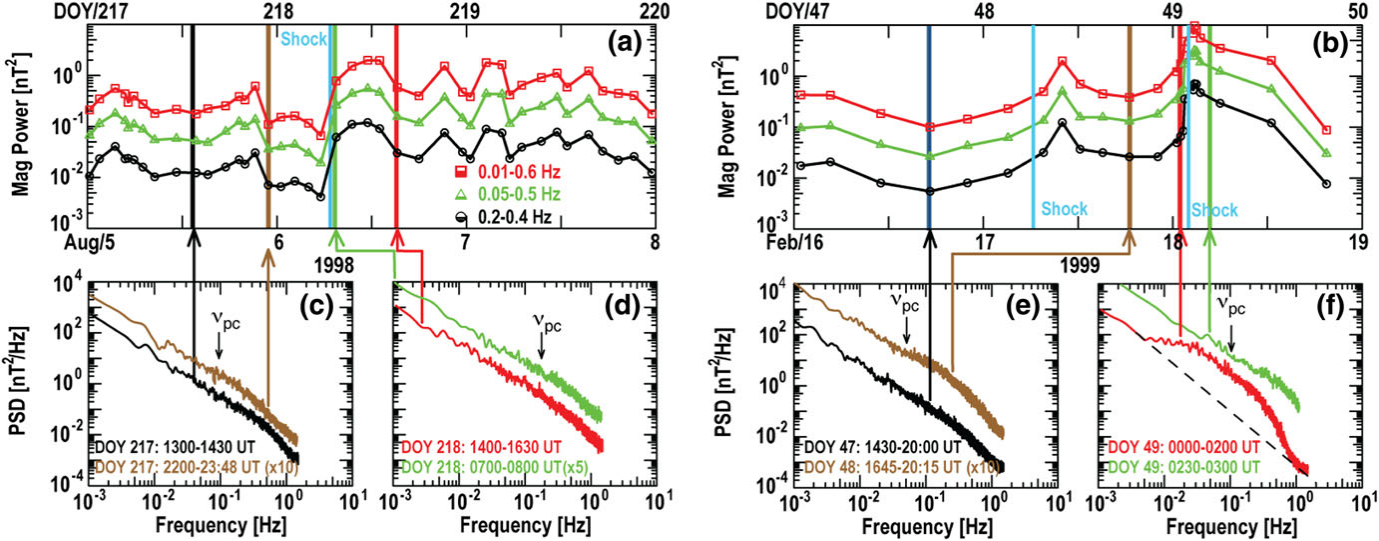
*Upper panels* ACE measurements of the magnetic power versus time in three different frequency ranges. *Lower panels* PSDs at four time intervals identified by the *vertical bars in the upper panels*. The local proton cyclotron frequency $\nu _{pc}$ is shown in each of the four *lower panels*. The two shocks arrived at ACE at $\sim$0644 UT on August 6, 1998, and $\sim$0211 UT on February 18, 1999, i.e., ${<}40$ min earlier than at Wind. ACE was located at the L1 point and separated from Wind by ${\sim }135\,R_{E}$ and ${\sim }164\,R_{E}$, respectively. Image reproduced with permission from Desai et al. (2012), copyright by AIP

Figure 38 shows the temporal evolution of the CNO energy spectra from Wind/STEP and O spectra from ACE/ULEIS for the two events in Fig. 37. Note that the ACE/ULEIS data are obtained by considering the time shift equal to the time it takes solar wind plasma to advect from one s/c to the other. These snapshots are taken at three separate intervals near the two IP shocks, as indicated by the color-coded bars in Fig 37a, b. The CNO spectra for the August 6, 1998 event for all three intervals have similar shapes, with no evidence of a distinctive roll over at lower energies during the upstream interval (red) compared with those measured at the shock (orange) and downstream (green). In contrast, the CNO spectrum upstream of the February 18, 1999 event shows a dramatic roll-over or flattening below ${\sim }200\,\mathrm{keV/nucleon}$ followed by unfolding or steepening as the shock and its associated lower-energy ion population approach the s/c, as predicted by DSA theory (Lee 1983, 2008).

Figure 39 shows the magnetic power versus time in three different frequency ranges (upper panels) and the power spectral densities or PSD (lower panels) at four different time intervals for the two events shown in Fig. 37. The PSD upstream (brown curve in Fig. 39c) of the August 1998 shock shows no increase in the energy of the fluctuations across the entire spectral range (${\sim }10^{-3}$–$1\,\mathrm{Hz}$) and has no significant wave activity around the proton cylcotron frequency, $\nu _{pc}$, i.e., at ${\sim }0.1$–$0.2\,\mathrm{Hz}$. In the case of the February 1999 IP shock, the energy in the fluctuations across the spectral range increases by about a factor of 10 ahead (brown curve in Fig. 39e) of the shock, while immediately upstream (red curve in Fig. 39f), the PSD shows a dramatic departure from a power-law (dashed line) due to significant wave growth between ${\sim }0.05$ and $1\,\mathrm{Hz}$, i.e., centered on $\nu _{pc}$ at ${\sim }0.1\,\mathrm{Hz}$. Two types of ion populations streaming away from the shock can generate such upstream waves: (1) beams of low-energy protons (e.g., Kennel et al. 1986), or (2) pre-accelerated SEP protons (e.g., Bamert et al. 2004). In either case, Figs. 37, 38 and 39 demonstrate that such wave excitation upstream of a shock can have a dramatic influence on the properties of the associated ESP event.

Desai et al. (2012) interpreted the observations of the August 6, 1998 quasi-perpendicular shock as evidence for shock-drift acceleration of a pre-existing suprathermal spectrum (see Desai et al. 2003, 2004). In contrast, the ESP event associated with the oblique IP shock on February 18, 1999, is an example of wave excitation and the first-order Fermi acceleration process; enhancements in the PSD around $\nu _{pc}$ indicate the presence of Alfvén waves excited presumably by the accelerated protons streaming away from the shock. The softening or unfolding of the CNO spectrum below ${\sim }200\,\mathrm{keV/nucleon}$ in Fig. 38b is likely due to M/Q-dependent trapping and scattering of these ions by the proton-excited waves. Finally, the near-zero values and the reversal of $A_{1}/A_{0}$ around the shock (see Fig. 37f) point to the shock as the source of the isotropic ion population.

## Ground level enhancements

Some large SEP events are so intense that the intensity of the accompanying high-energy (${>}1\,\mathrm{GeV}$) protons exceeds the lower-energy portion of the galactic cosmic ray (GCR) background in at least one of the many ground-based neutron monitors (e.g., Lopate 2006), muon detectors (Falcone et al. 2003; Abbasi et al. 2008), or ionization chambers (Forbush 1946). Such increases in the radiation levels on Earth’s surface that are detected by neutron monitors are commonly known as GLEs. Thus, GLEs are SEP events in which the particle acceleration process is more efficient (Meyer et al. 1956). These events pose severe radiation hazards to astronauts and technological assets in space and disrupt airline communications (Shea and Smart 2012). The origin of GLEs has generated much interest since they were first detected (Forbush 1946). Only recently has sufficient progress been made such that it is now widely accepted that they are a result of CME shock acceleration in the low corona below ${\sim }2\,R_{S}$. In this section, we summarize the body of evidence that points to this conclusion.

### Velocity dispersion and timing studies

**Fig. 40:**
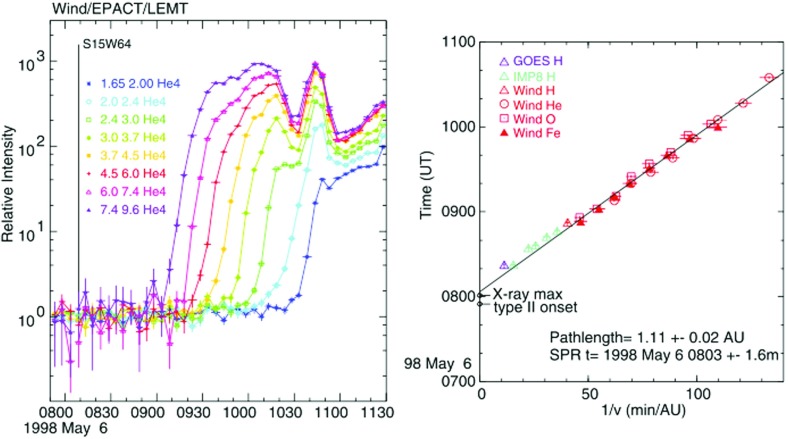
*Left* Arrival of $^{4}$He ions of different energies at the Wind s/c during the May 6, 1998 GLE. *Right* Ion intensity onset time in each energy interval versus 1/v. Parameters of the linear fit (*solid line*) are: slope gives the path-length, and the intercept yields the SPR time at the Sun. Image reproduced with permission from Reames (2009b), copyright by AAS

During the onset phase of SEP events, the arrival times of high-energy ions and electrons are governed largely by velocity dispersion effects; ions and electrons with the highest speeds arrive first and the slower particles arrive later (see Fig. 23). Thus, by measuring the arrival times of the earliest arriving particles and assuming that they travel essentially scatter-free from the Sun to the Earth, one can estimate the solar particle release (SPR) times at the Sun to within ${\sim }3$-min uncertainty (see Rouillard et al. 2012). Figure 40 shows an example of the timing analyses based on ${>}2\,\mathrm{MeV/nucleon}$ H, He, O and Fe ions observed during the onset phase of the May 6, 1998, large SEP event. By using multiple species at different energies, the SPR time at the Sun for this event was estimated to be at $\sim$0803 UT on May 6, 1998, well after the onset of the type II radio burst and the maximum of the associated X-ray flare.

**Fig. 41:**
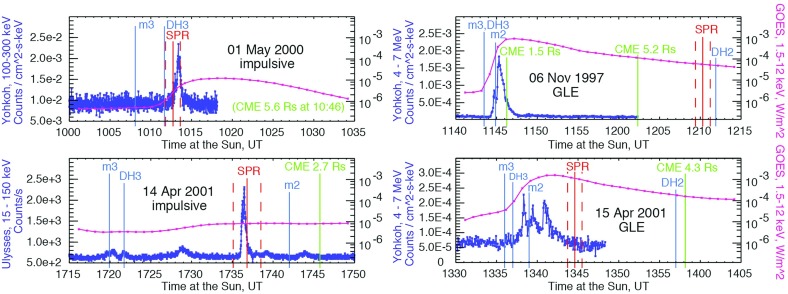
Onset times for two impulsive SEP events (*left*) and two GLEs (*right*). *Blue curves* show the intensities of hard X-rays (*left panels*) and of 4–7 MeV $\gamma$-rays (*right panels*). The *red curves* are the GOES 1.5–12 keV soft X-ray intensities. The *red vertical lines* represent the SPR times inferred from measuring the arrival times of the GLE particles. Also shown are onset times of type II and type III radio bursts and CME lift-off times (after Tylka et al. 2003)

Figure 41 compares the SPR times at the Sun, estimated from onset time versus 1/v scatterplots like the one shown in Fig. 40, with the solar X-ray and $\gamma$-ray emission profiles. The two left panels show that the SPR times for the two impulsive SEP events agree well with the timing of the hard X-ray peaks, but in the case of both GLEs, the SPR times occur several minutes after the $\gamma$-ray peaks. These results are consistent with the earlier results of Cliver et al. (1982), who found that the release times of $\sim$ GeV protons and the SEP onset times were poorly correlated with photon emission from the associated flares, while for impulsive events, the flare and SEP onset times were well correlated (see Reames et al. 1985; Reames and Stone 1986).

**Fig. 42:**
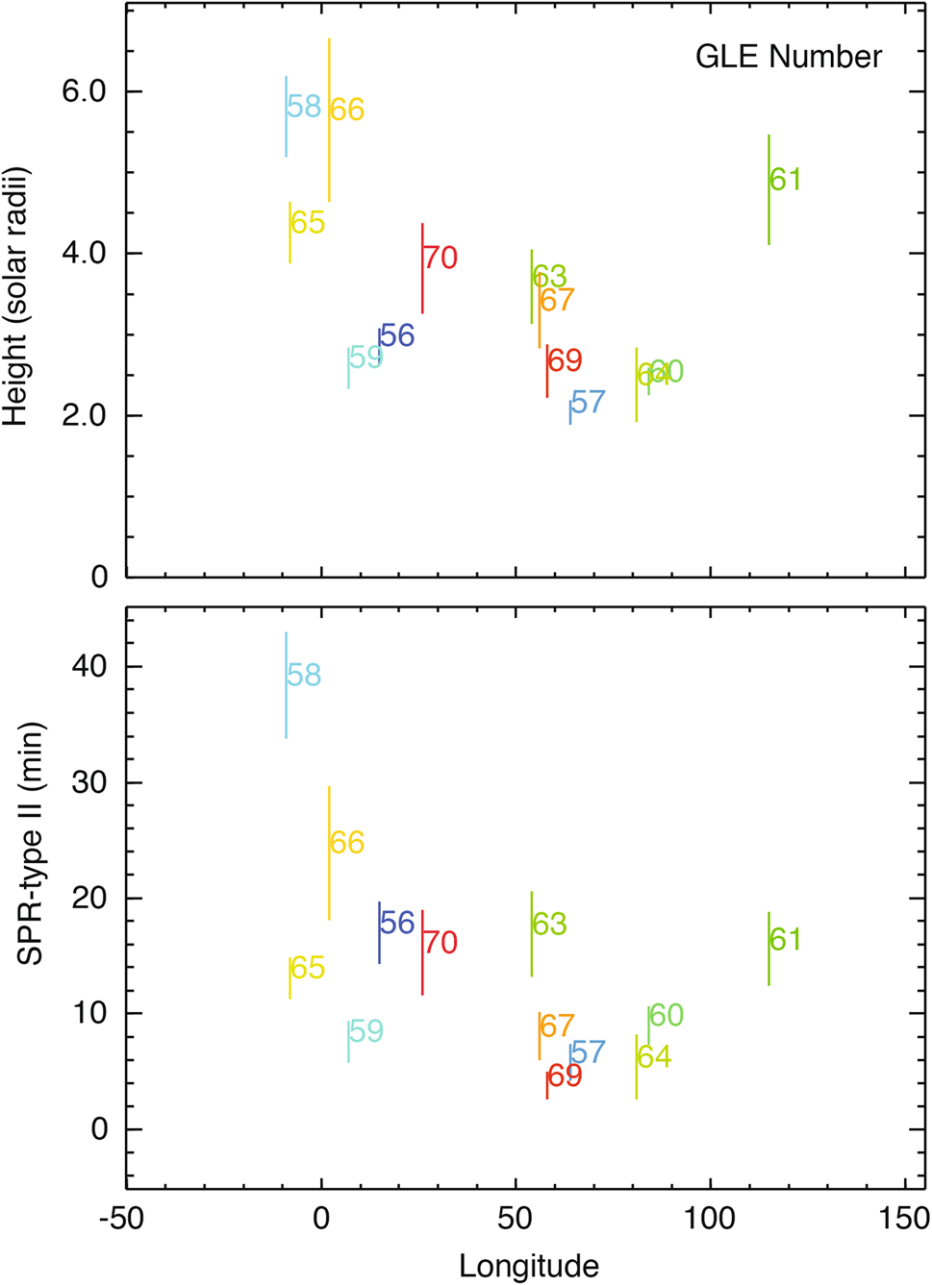
*Top* Radial height of CME shocks at the time of the first SPR versus flare longitude. *Bottom* SPR time—the type II onset time at the Sun versus source longitude during several GLEs. Image reproduced with permission from Reames (2009b), copyright by AAS

Using the onset times of energetic particles of various species and velocities, *v*, in 13 large SEP events that were also associated with GLEs, Reames (2009a, (2009b) reported that the SPR times occurred after the onset of the CME shock wave-induced type II radio emission and that SEP events with well-defined SPR times occurred over a wide span in solar longitude (see Fig. 42b). Reames (2009b) suggested that, regardless of source longitude, all ion species and energies are released together in all these GLEs, with no evidence of energy- or rigidity-dependent coronal transport, which essentially rules out a flare origin for GLEs. This is because particle populations accelerated and released from a point source, such as an active region or a flare, have to be transported across the coronal magnetic field lines through substantially denser material, and would therefore exhibit properties (e.g., abundance ratios) that depend on distance between the observer’s connection point and the source longitude (see, e.g., Mason et al. 1984). By converting the SPR time to a radial distance of the CME shock wave from the Sun at the release time (see Fig. 42a), Reames (2009b) noted that acceleration for well-connected events begins at ${\sim }2$–$4\,R_{S}$ within ${\sim }100^{\circ }$ of the source longitude and rises to greater heights at longitudes more distant from the source, as would be expected from CME shock-acceleration models.

### Energy spectra, composition and charge states

**Fig. 43:**
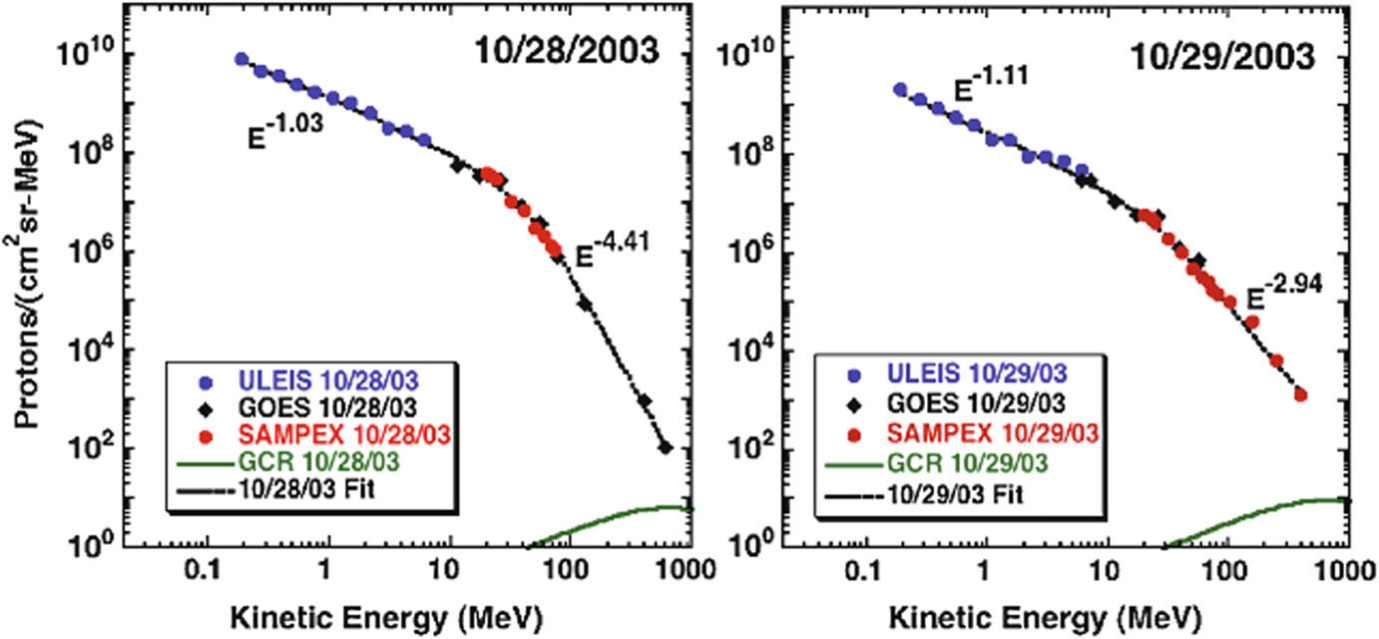
Proton fluence spectra during two GLEs observed during the October–November (Halloween) 2003 SEP events. Both spectra are fitted with the double power-law function of Band et al. (1993), with different spectral slopes above and below the break energy. *Green curves* show the GCR fluence levels. Image reproduced with permission from Mewaldt et al. (2012a), copyright by Springer

Surveying the ${\sim }0.1$ to ${\sim }500$–$700\,\mathrm{MeV}$ proton energy spectra in 16 GLEs that occurred during solar cycle 23, Mewaldt et al. (2012a) found that the spectra exhibited breaks between ${\sim }2$ and $50\,\mathrm{MeV}$ and were better represented by a double power-law function, as shown in Fig. 43. This study also showed that, in comparison with other SEP events, the proton spectra associated with GLEs are harder, with spectral indices $\gamma {\sim }3$ above 40 MeV/nucleon.

Mewaldt et al. (2012a) reported that ${\sim }50\,\%$ of GLEs exhibit properties generally associated with “flare material” as observed in impulsive or $^{3}$He-rich SEP events (e.g., Mason et al. 1994, 2004; Reames et al. 1994)—including enrichments in Fe/O, Ne/O, $^{22}$Ne/$^{20}$Ne, and highly-ionized charge states of Fe—and that the fraction of GLE events that was Fe-rich during solar cycle 23 was significantly lower than that seen during solar cycles 21 and 22 (see also Dietrich 1999; Dietrich and Tylka 2003; Tylka et al. 2005). Like the ESP events and large gradual SEP events discussed above, possibilities that could account for flare signatures during GLE events are: (1) shock acceleration of suprathermal ions left over from previous impulsive SEPs (Mason et al. 1999; Tylka et al. 2005; Mewaldt et al. 2006; Tylka and Lee 2006; 2) mixing of flare- and CME shock-accelerated particles (Cane et al. 2003, 2006; 3) CME shock acceleration of a mixture of solar wind and flare particles (Li and Zank 2005; 4) CME shock acceleration of a mixture of suprathermals and material from the CME ejecta (Mewaldt et al. 2007a); and (5) acceleration of an admixture of flare material and material from a preceding CME shock (Li et al. 2012).

Mewaldt et al. (2012a) also modeled electron stripping during CME shock acceleration in the low corona and were able to account for the higher mean Fe charge states of ${\approx }20$ during 5 GLEs by assuming that the acceleration process started at ${\sim }1.24$–$1.6\,R_{S}$, which is consistent with recent comparisons of CME propagation and type II radio bursts. Kahler et al. (2012) noted that some GLEs are also associated with shorter duration (${<}1$ h) flares—timescales that are comparable to those associated with the impulsive $^{3}$He-rich SEP events. In surveying the relationships between the SEP electron/proton (e/p) and Fe/O ratios and characteristics of the associated flares, active regions (AR), and CMEs for ${\sim }40$ GLEs observed since 1976, Kahler et al. (2012) noted that the e/p and Fe/O abundance ratios trended toward typical values seen in large gradual SEP events with increasing flare timescales, thermal and non-thermal peak fluxes, and active region sizes. Kahler et al. (2012) concluded that these results and the wide range of solar longitude connections for GLEs (see Fig. 42) with high abundance ratios argue against a significant role for flare contributions in GLEs, and instead point to acceleration at CME-driven shocks as the dominant mechanism for GLEs.

### Radiation hazards

As discussed in Sect. 2, the largest SEP events occur as a result of acceleration by coronal and interplanetary shocks with speeds ${>}2000\,\mathrm{km/s}$ that are driven by CMEs with kinetic energies ${>}1\times 10^{32}\,\mathrm{ergs}$ and widths ${>}100^{\circ }$ in longitude (e.g., Gopalswamy 2006; Mewaldt et al. 2008). Although shock acceleration occurs in a variety of space plasma environments and the basic physical mechanisms are reasonably well understood, the relative roles of various factors that control a given shock’s efficiency are not fully identified. These factors combine to cause large variations from one SEP event to the next, making it difficult to forecast key properties such as peak intensities and fluences, maximum energies, and temporal, spatial, spectral, and compositional evolution.

**Fig. 44:**
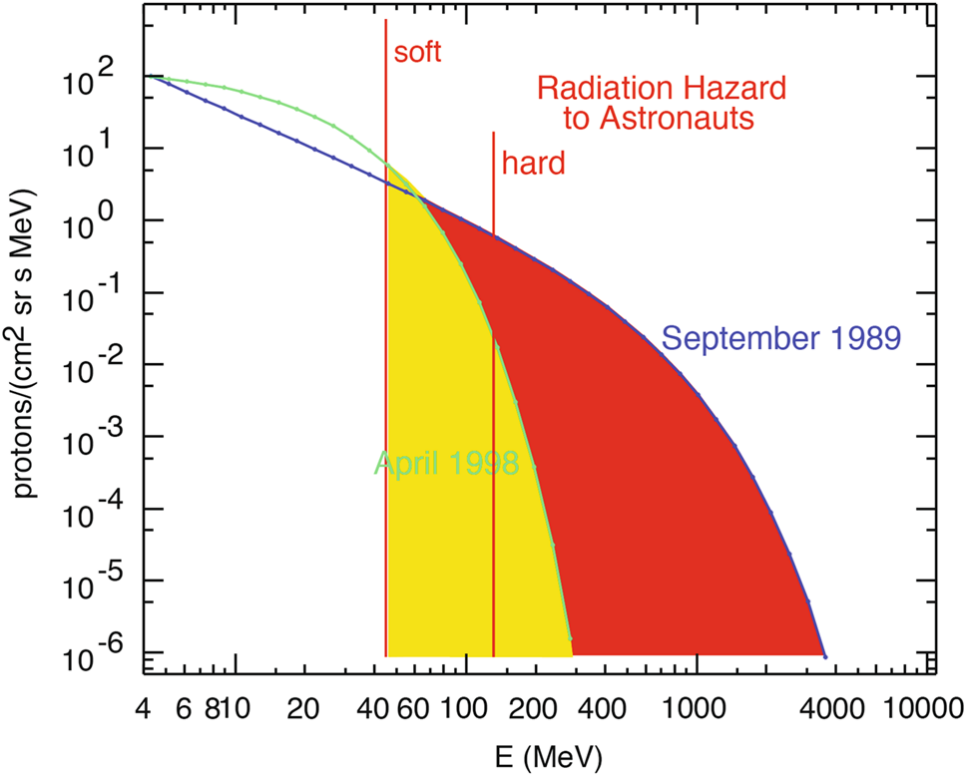
Proton energy spectra during the SEP events of April 1998 (*green* Tylka et al. 2000) and September 1989 (*blue* Lovell et al. 1998). *Yellow* hazardous radiation portion of the spectrum during the April 1998 event; *red* additional hazardous radiation from the September 1989 event. Image reproduced with permission from Reames (2013), copyright by Springer

Understanding the origin of event-to-event variations in CME-related SEP events has therefore become a top priority for heliophysics, since high-energy protons in the largest events pose severe radiation hazards for humans and technological systems in space, particularly as humankind continues its quest to venture outside the protective cocoon of the Earth’s magnetic field (e.g., Cucinotta et al. 2010; Schwadron et al. 2010b; Xapsos et al. 2012). Most of the radiation risk from SEPs is due to intense fluxes of ${\sim }50$–$200\,\mathrm{MeV}$ protons—the energy at which protons can penetrate s/c housing and spacesuits. The radiation hazards associated with SEP events can be assessed in terms of the roll-over or knee energy—the energy at which the particles escape from the shock and the spectrum steepens (Ellison and Ramaty 1985). Typical knee energies of soft and hard radiation SEP events are shown in Fig. 44; events with higher roll-over or knee energies have significantly higher proton intensities above ${\sim }100\,\mathrm{MeV}$ and can pose a severe radiation hazard to astronauts (Reames et al. 2001; Reames 2013).

**Fig. 45:**
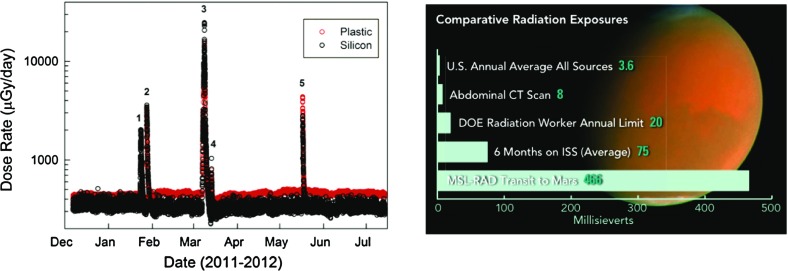
*Left* Dose rates ($\sim$16-min averages) recorded by MSL-RAD in a silicon detector (*black circles*) and in a plastic scintillator (*red circles*) during MSL’s journey to Mars. Five SEP events were observed during the cruise phase. For a given incident flux, the dose rate in silicon is generally less than the dose rate in plastic because of the comparatively large ionization potential of silicon. *Right* Radiation exposure compared with that measured by MSL-Rad on its way to Mars. Image reproduced with permission from Zeitlin et al. (2013) and Kerr (2013), copyright by AAAS

Figure 44 shows that an important factor in determining the ionizing radiation dose associated with SEP protons is the location of the spectral break (Reames et al. 2001). The figure shows that, although the April 1998 SEP event had higher fluxes below ${\sim }50\,\mathrm{MeV}$, the spectrum rolls over much more rapidly at higher energies. In contrast, the September 1989 event had somewhat lower intensities below ${\sim }50\,\mathrm{MeV}$, but because the spectral break occurred between ${\sim }200$ and $300\,\mathrm{MeV}$, it was associated with a significantly higher radiation dose. In fact, during the September 1989 event, even an astronaut shielded by $10\,\mathrm{g/cm}^{2}$ of material would have received an ionizing dose of ${\sim }40$ millisieverts (mSv). The annual dose limit for a radiation worker in the United States is 20 mSv (see Fig. 45). The unit Sv takes account of the relative biological damage from different types of ionizing radiation (e.g., X-rays, $\gamma$-rays, cosmic rays) and is defined as the amount of ionizing radiation equivalent to 1 gray or 100 rads of $\gamma$-radiation; 1 mSv corresponds to 10 ergs of energy of $\gamma$ radiation transferred to 1 g of living tissue.

Recently the radiation assessment detector (RAD) on board the Curiosity rover, which was carried by the Mars Science Laboratory (MSL) on its way to Mars during 2012, provided direct measurements of the radiation that astronauts could encounter as they voyage beyond Earth’s protective magnetic field (Kerr 2013; Zeitlin et al. 2013) during this particular phase of the solar cycle. After converting the MSL-RAD measurements into dose rate and total dose, Zeitlin et al. (2013) concluded that, during a 360-day round trip, an astronaut would receive a dose of about 662 mSv (see Fig. 45). The amount of radiation is cumulative and increases the overall lifetime cancer risk for an astronaut. The dosage measured by this experiment is considerable when compared with an exposure limit of ${\sim }1000\,\mathrm{mSv}$ or less during an astronaut’s entire career. Note, however, that in terms of sunspot numbers, CMEs, and flare occurrences, the current solar activity cycle is the weakest of the Space Age (e.g., Wang and Colaninno 2014), and a manned mission to Mars is not likely to occur until well after the next solar maximum in 2021–2023, when the Sun could unleash more powerful CMEs even more frequently and cause astronauts to reach their exposure limits perhaps during part of their journey.

Using flare and CME observations to predict the size and impact of SEP events is highly unreliable. Nonetheless, flares can be useful for predicting the occurrence of SEP events (e.g., Balch 2008), while relativistic electrons have been used as a precursor for predicting the arrival of ${<}50\,\mathrm{MeV}$ protons (Posner 2007). Additionally, Kahler (2001) showed that the peak intensities of ${\sim }110$–$500\,\mathrm{MeV}$ protons are correlated with the CME speed. Thus, an early measure of the CME or shock speed as inferred from coronagraph observations or from the drift rate of the accompanying type II burst could be used to forecast SEP intensity. Unfortunately, ${>}50\,\mathrm{MeV}$ protons, which cause much of the radiation damage, tend to arrive within ${\sim }10$–30 min of the occurrence of the X-ray flare, detection of the type II radio burst, or arrival of relativistic electrons at Earth. This leaves very little time for astronauts performing extra vehicular activity (EVA) on the lunar surface or on the International Space Station (ISS) to take appropriate evasive action.

### How big was the SEP event associated with the Carrington event?

**Fig. 46:**
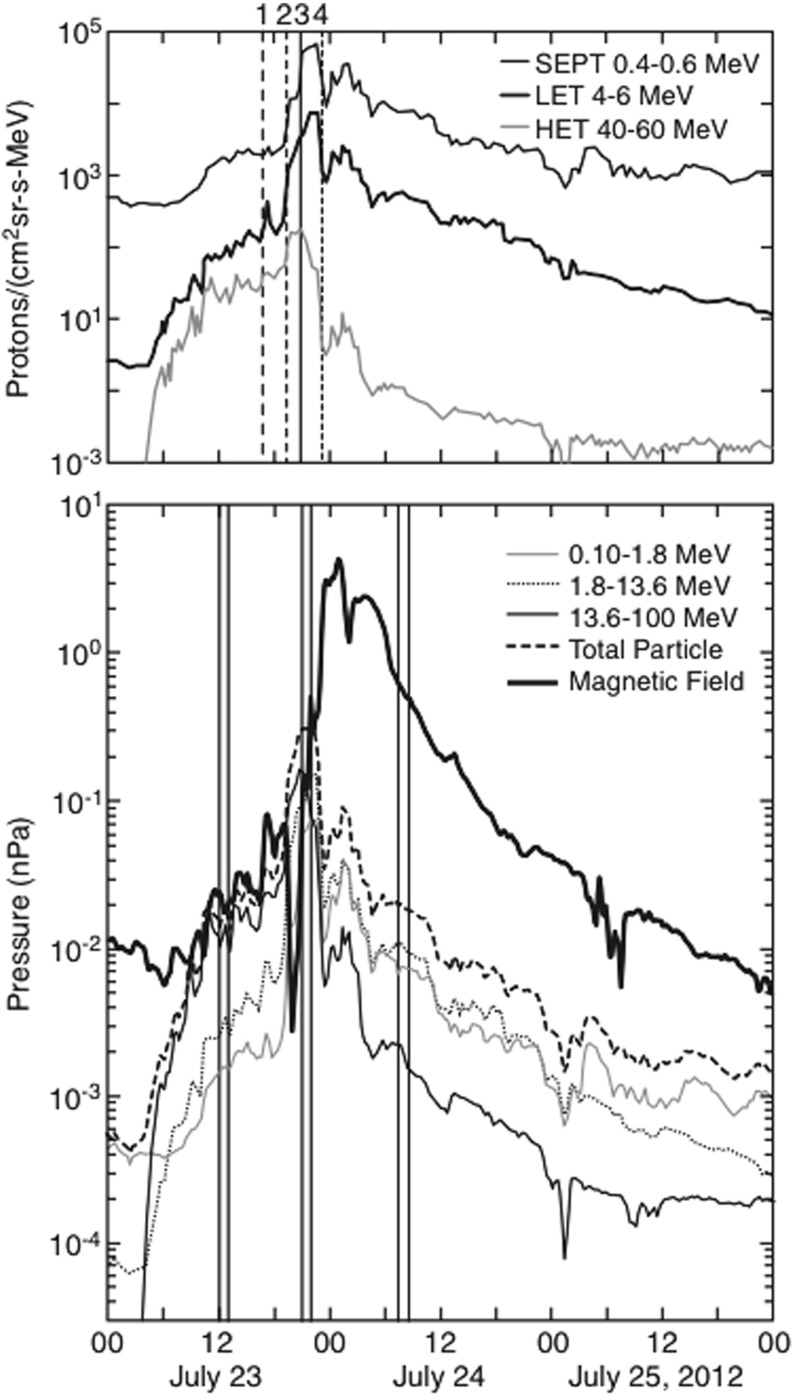
*Top* 10-min averaged energetic proton intensities measured on STEREO A in three different energy ranges: SEPT from 0.4–0.6 MeV, LET from 4 to 6 MeV, and HET from 40 to 60 MeV. *Bottom* The energetic proton pressure in three energy ranges and their total during the event. Magnetic pressure is included for comparison. Image reproduced with permission from Russell et al. (2013), copyright by AAS

The Carrington event, discussed in Sect. 1.1, was associated with probably one of the strongest and fastest CMEs that occurred over the last ${\sim }200$ years. More recently, by July 23, 2012, a solar active region being tracked by inner heliosphere s/c had already produced four fairly fast Earthward-directed CMEs. This region then released one of the fastest CMEs on record in the direction of STEREO A, which was well away from Earth. The CME had a speed of ${>}2000\,\mathrm{km/s}$ and was accompanied by an unusually intense SEP event at STEREO A (see Fig. 46). At the same time, Earth was near the edge of the SEP cloud, therefore ACE and Wind at L1 observed a modest SEP event. At STEREO A, the SEP energy density (pressure) was comparable to that of the magnetic field, which weakened the CME-driven shock wave. Such effects are thought to occur in supernovae shocks but have not been previously observed inside the heliosphere. Even though the initial shock was weak, the solar ejecta and magnetic cloud that followed had speeds above ${\sim }2000\,\mathrm{km/s}$ and contained strong magnetic fields. This event shows that the Sun can produce extreme events even during relatively modest sunspot activity cycles and that Carrington-sized events may be frequent.

While the Carrington event has been extensively studied and discussed in terms of the associated flares, CME, and intense geomagnetic storm, it remains unclear exactly how big the associated SEP event was. In fact, this topic has been highly controversial ever since McCracken et al. (2001) postulated that spikes in the concentration of nitrates ($\mathrm{NO}^{-}_{3}$) in polar ice cores might also provide a quantitative measure of large SEP events over the past ${\sim }500$ years. On the basis of these data, McCracken et al. (2001) and Shea et al. (2006) interpreted a large $\mathrm{NO}^{-}_{3}$ spike observed around the late 1800s as a signature of a large SEP event associated with the Carrington event. In contrast, studies using different polar ice core records did not show similar evidence of an SEP event associated with the Carrington event, and instead indicated that all the $\mathrm{NO}^{-}_{3}$ spikes were accompanied by chemical tracers that pointed to anthropogenic biomass burning, and therefore, the ice core $\mathrm{NO}^{-}_{3}$ spikes constitute an unreliable proxy for large SEP events that occurred in the past (Wolff et al. 2012). More recently, Duderstadt et al. (2014) investigated whether the nitrate spikes seen in snow samples from August 2000 to August 2002 at Summit, Greenland are associated with large solar proton events (SPEs). Specifically, they identified tropospheric sources of nitrates and used the three-dimensional global Whole Atmosphere Community Climate Model (WACCM) to conclude that while the November 9, 2000, SPE significantly enhanced the mesospheric and stratospheric nitrate levels, the associated atmospheric nitrate-column density is still too low for deposition in the surface snow. This requires alternative proxies for studying historical SPEs and their terrestrial impacts.

Many researchers have also surveyed naturally existing archives of cosmogenic radionuclides such as $^{14}$C and $^{10}$Be that are produced by high-energy galactic cosmic rays interacting with the Earth’s atmosphere and deposited in, for example, tree rings or ice cores on the surface (e.g., Beer et al. 2003; Usoskin and Kovaltsov 2012; Usoskin 2013). Since particles with GeV energies are required to produce these signatures, only those SEPs that produce significant proton fluences above GeV energies, i.e., GLEs, are likely to initiate atmospheric cascades and generate such signatures on the Earth’s surface. Surveying over ${\sim }12{,}000$ years of these natural records, Usoskin and Kovaltsov (2012) identified 23 candidate episodes where the accompanying SEP events had an average flux above 30 MeV of ${>}10^{10}\,\mathrm{cm}^{2}$, but the Carrington event did not even feature in this list. In other words, it appears that the Carrington event did not leave any tell-tale signatures in the cosmogenic isotope records, and therefore, somewhat surprisingly, may not have been associated with a very large SEP event at Earth.

## Properties of suprathermal ion populations

### Spectral behavior

Until a decade or so ago, large gradual SEP events and IP shock-associated ESP events were believed to occur when fast CME-driven shock waves accelerated material out of the ambient corona or the thermal solar wind (e.g., Reames 1999). However, measurements from ACE (Stone et al. 1998) have shown that CME-driven shocks routinely accelerate tracer ion species like $^{3}$He and $\hbox {He}^{+}$ near 1 AU (Desai et al. 2001, 2003; Kucharek et al. 2003) and near the Sun (Cohen et al. 1999; Mason et al. 1999; Desai et al. 2006a). Although both the $^{3}$He and $\hbox {He}^{+}$ ions are extremely rare in the solar wind (relative abundance ratios are of the order of ${\sim }10^{-4}$), they are more abundant in the suprathermal energy region between ${\sim }1.5$ and 10 times the solar wind speed (Gloeckler 2003; Desai et al. 2006b). The $^{3}$He ions are probably accelerated in impulsive solar-flare related SEP events (e.g., Mason et al. 2004), while the $\hbox {He}^{+}$ ions originate as interstellar neutral atoms that get ionized when they enter the inner solar system within ${\sim }0.2\,\mathrm{AU}$ (e.g., Ruciński et al. 1996). These observations provide compelling evidence that CME-driven shocks accelerate material preferentially out of the suprathermal pool, which also comprises heated solar wind or coronal material (e.g., Mason et al. 2005), thereby forcing a re-examination of where the seed population for large SEP and ESP events originated.

**Fig. 47:**
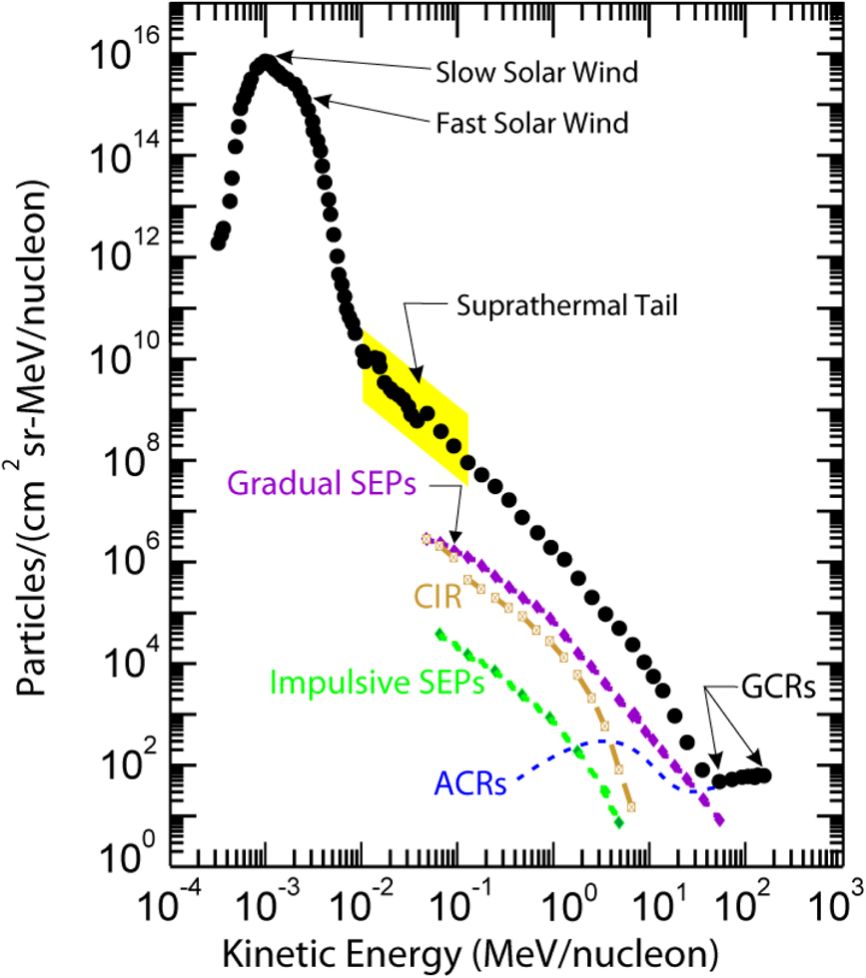
Oxygen fluences measured by several instruments on board ACE during a 3-year period. The *black data points* show the fast and slow solar wind components along with a suprathermal tail that extends well above ${\sim }100\,\mathrm{MeV/nucleon}$. Also shown are representative particle spectra obtained for gradual and impulsive SEPs, corotating interaction regions (CIRs), anomalous cosmic rays (ACRs), and GCRs. Image adapted from Mewaldt et al. (2001)

Figure 47 shows the oxygen fluences from solar wind to cosmic ray energies obtained by several ACE instruments from October 1997–June 2000 (from Mewaldt et al. 2001). The suprathermal (ST) energy region is defined here as that between ${\sim }2$ and $100\,\mathrm{keV/nucleon}$ and is shaded in the figure. Note the presence of a continuous distribution extending from the solar wind peaks through the suprathermal region out to cosmic ray energies. Figure 47 also shows the energy spectra measured during various sources and populations that can contribute to this energy region. In addition to these sources, the suprathermal pool includes interstellar and inner source pickup ions and the heated solar wind (e.g., Mason et al. 2005).

**Fig. 48:**
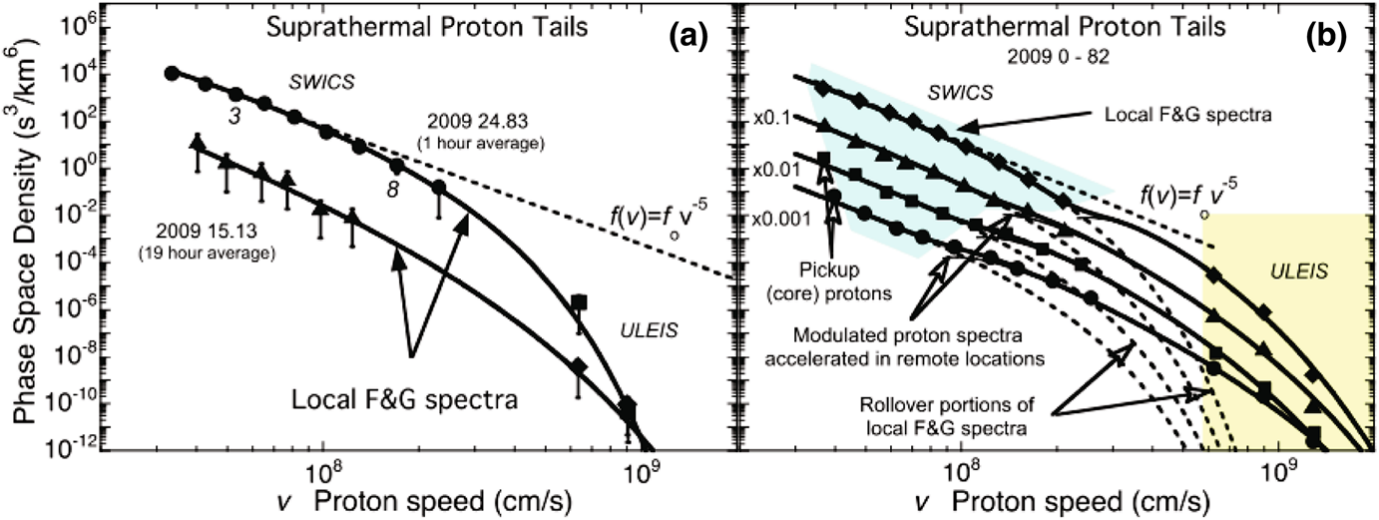
Phase space density during the extreme quiet SW conditions in 2009. **a** Highest and lowest observed tail densities during the first 82 days of 2009. **b** Four spectra selected according to their tail densities. Image reproduced with permission from Fisk and Gloeckler (2012), copyright by Springer

Figure 48 shows that ST proton tails typically extend up to ${\sim }20$ times the solar wind speed (${\sim }200$–$700\,\mathrm{keV/nucleon}$) under a variety of solar wind conditions as measured at Ulysses and ACE. One key feature of these tails is the apparent near constancy of the slope of the power-law distribution; $v^{-5}$ in particle velocity, *v*, or $E^{-1.5}$ in particle energy, *E*, during “quiet” solar wind conditions (Gloeckler et al. 2008).

**Fig. 49:**
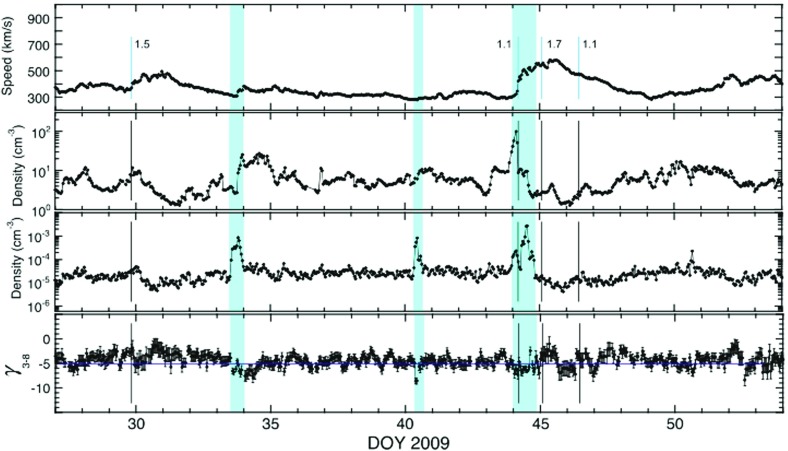
Solar wind parameters, the density of the suprathermal proton tail, and the spectral index of the ST tail for the extreme quiet conditions in the solar wind during 2009. Possible shocks are marked with thin *vertical lines*; the compression ratio across the shock is also shown. The *shaded regions* indicate time periods during which spectra shown in Fig. 48 were taken. Image reproduced with permission from Fisk and Gloeckler (2012), copyright by Springer

Figure 49 shows the temporal profiles of the SW proton speed and density, and the density and spectral index of the ST proton tail during 2009. The figure shows large increases in the proton tail density in SW compression regions, which are associated with increases in the solar wind speed, temperature or thermal speed, and the solar wind proton density. Analyzing ACE/SWICS 1 h-averaged ST tail densities in 61 possible IP shocks during the extremely disturbed conditions of 2001, Fisk and Gloeckler (2012) found that unless the shock was associated with an extended compression region, it produced only a small or no tail density increase. In contrast, enhancements in the ST tail density, which indicate particle acceleration, were almost always associated with an extended compression region and had a common $v^{-5}$ spectral slope. In the four events that could be due to DSA, the spectral index did not match the steady-state DSA theoretical predictions. In Sects. 3.2 and 3.3, we discussed possible reasons why this may occur.

In contrast to the Fisk and Gloeckler (2012) study, Giacalone (2012) selected 18 strong shocks over the 1998–2003 period and used ${\sim }5$-min resolution ACE/EPAM energetic proton data to show that the largest intensities of ${\sim }47$-keV ions, which are part of the ST tail, nearly always occurred within 5 min of the passage of a strong IP shock. Giacalone (2012) also noted that there was no additional increase in the particle intensity in the region behind the shock in some of the cases, which suggests that, at least for these cases, there is no additional acceleration there. Giacalone (2012) concluded that the acceleration occurs directly at the shock, where the plasma compression is the largest, and essentially ruled out any acceleration in the turbulent plasma behind it. In summary, the acceleration of the ST tail densities remains a subject of much debate, as different studies appear to highlight the relative importance of different plasma and interplanetary structures in the solar wind.

**Fig. 50:**
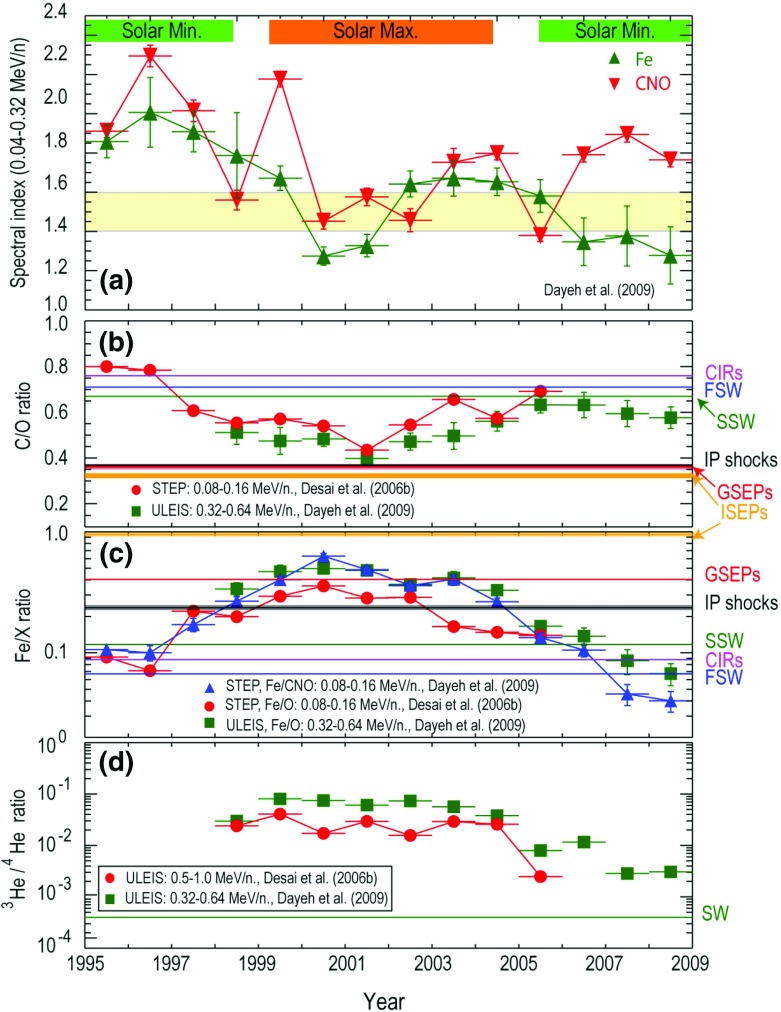
Yearly averages of the quiet-time suprathermal *a* Fe and CNO spectral indices, $\gamma$, given by fitting a power-law of the form $j=j_{0}E^{-\gamma }$ to the measured differential intensities *j* at energy *E*; *E* is in MeV/nucleon, *b* C/O ratio, *c* Fe/CNO ratio, and *d* $^{3}$He/$^{4}$He ratio measured by the Wind/STEP and ACE/ULEIS from 1995–2009 (adapted from Desai et al. 2006b; Dayeh et al. 2009)

### Abundances

**Fig. 51:**
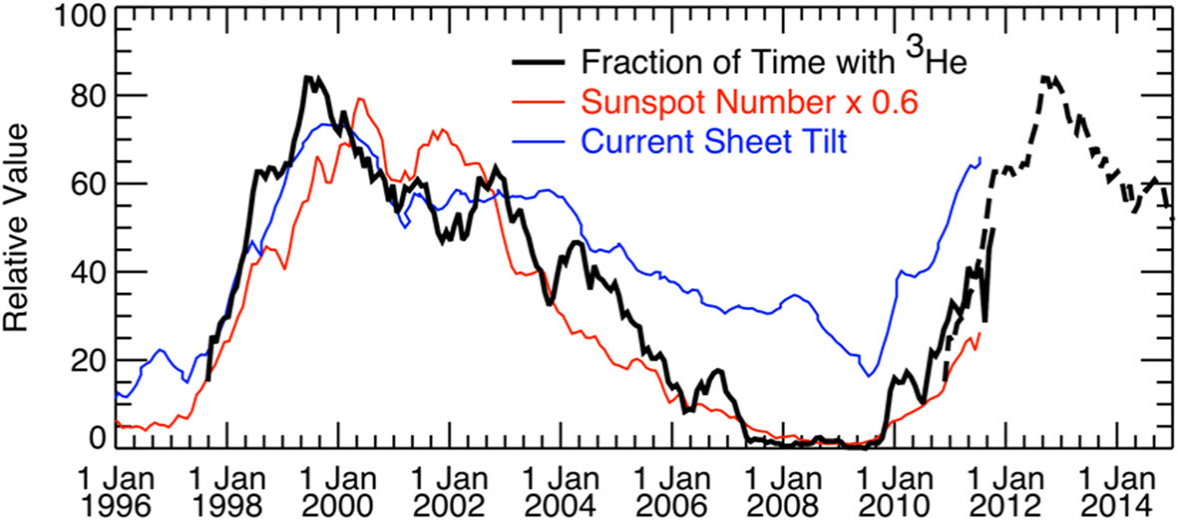
Fraction of time that energetic $^{3}$He above ${>}$MeV/nucleon is present in the interplanetary medium, compared with the sunspot number, and current sheet tilt (adapted from Wiedenbeck and Mason 2013)

The above situation is exacerbated by the fact that current observational studies of ST ion populations are conflicting (see Mason and Gloeckler 2012). In particular, observations of ST tails between ${\sim }6$ and 20 times the solar wind speed (Dayeh et al. 2009) and above a few MeV/nucleon (Mewaldt et al. 2007b) indicate that that the spectra do not conform to a single power-law but are somewhat variable, with indices ranging from $-4.5$ to $-6.5$ (see Fig. 50a). Moreover, Giacalone (2012) noted that the power-law part of the distributions observed just behind strong interplanetary shocks also have a range of spectral indices, but are generally flat (near $-4$). As shown in Fig. 50c, d, Desai et al. (2006b) and Dayeh et al. (2009) reported that the suprathermal heavy ion composition near 1 AU varies with solar activity; it is dominated by CIR- or solar wind-like material during solar minimum and by impulsive SEP-like ions during periods of increased solar activity. Thus, the suprathermal population appears to be highly dynamic and varies on long (solar cycle) and probably also on shorter ($\sim$ hours) timescales. Indeed, Fig. 51 (from Wiedenbeck and Mason 2013) shows that: (1) the fraction of time that $^{3}$He ions from impulsive flares are present at 1 AU varies from one solar rotation period to the next; and (2) the presence of $^{3}$He ions is significantly diminished as solar activity decreases, essentially reducing to zero during the extended solar minimum of 2008–2010.

### Theoretical ideas regarding ST origins

The considerable disparity in observational aspects of ST populations has led to two basic categories of competing theories: (1) ST tails result from continuous acceleration in interplanetary space (e.g., Fisk and Gloeckler 2008, 2012, 2014; Zhang 2010; Fahr et al. 2012; Drake et al. 2012; Zank et al. 2014), or (2) ST tails are lower energy portions of material accelerated in energetic particle events such as CIRs, CME shocks, flares, etc. (e.g., Livadiotis and McComas 2009; Jokipii and Lee 2010; Schwadron et al. 2010a). Table 2 summarizes the mechanisms and sources of ST ions and provides citations for their primary proponents. Existing ST ion observations pose different challenges for both types of models. For instance, the latter models are favored by observations above ${\sim }6$ times the SW speed where the spectral slopes vary considerably and the heavy ion composition (e.g., $^{3}$He) varies with solar activity. In contrast, the near constant spectral shape for ions with speeds ${\lesssim }5$ times the SW speed seems to favor the former types of models and poses challenges for discrete event origin scenarios.

**Table 2:** Known sources and mechanisms of $\sim$2–100 keV/nucleon suprathermal ions near 1 AU

Acceleration location	Continuous acceleration in IP space		Discrete high-energy particle events	
	Mechanism/source	References	Mechanism/source	References
Local	Bulk velocity fluctuations	Fahr and Siewert (2012) and Fahr et al. (2012)	CIR shocks and compressions	Fisk and Lee (1980), Giacalone et al. (2002) and Richardson (2004)
	Compressional turbulence	Fisk and Gloeckler (2006, (2008, (2012, (2014)	CME shocks (ESP events)	Jones and Ellison (1991), Lee (2005), Zank et al. (2006) and Li et al. (2009)
	Waves and turbulence in the IMF	Schwadron et al. (1996), Zhang (2010) and Zhang and Lee (2013)		
	Reconnection between magnetic islands	Drake et al. (2012) and Zank et al. (2014)		
			SEPs from flares	Mason et al. (2002)
Remote	N/A		SEPs from CME shocks near Sun	Reames (1999)
			CIR shocks ${>}2$ AU	Fisk and Lee (1980)
			Upstream ion events	Lee (1982) and Desai et al. (2008)

In other words, models advocating continuous acceleration processes could account for the constant spectral shapes, but it is not obvious why or how such processes could produce ST composition resembling the more energetic CIRs or SEPs and cause variations with solar activity. In addition, many ST ion studies have used long-term averages (e.g., Gloeckler et al. 2008; Dayeh et al. 2009), thereby making it difficult to distinguish between ST contributions from local and remote sources. Despite the difficulties in measuring the ST ions in the heliosphere, an improved understanding of the ST ion properties, origins, and acceleration mechanisms *is urgently needed* to achieve closure with SEP and CIR particle acceleration models because such pre-accelerated populations are clearly injected into shock acceleration processes more efficiently when compared with the more abundant solar wind ions (e.g., Mason et al. 1999).

## Multi-spacecraft observations of SEP events

### Particle reservoirs and spectral invariance

**Fig. 52:**
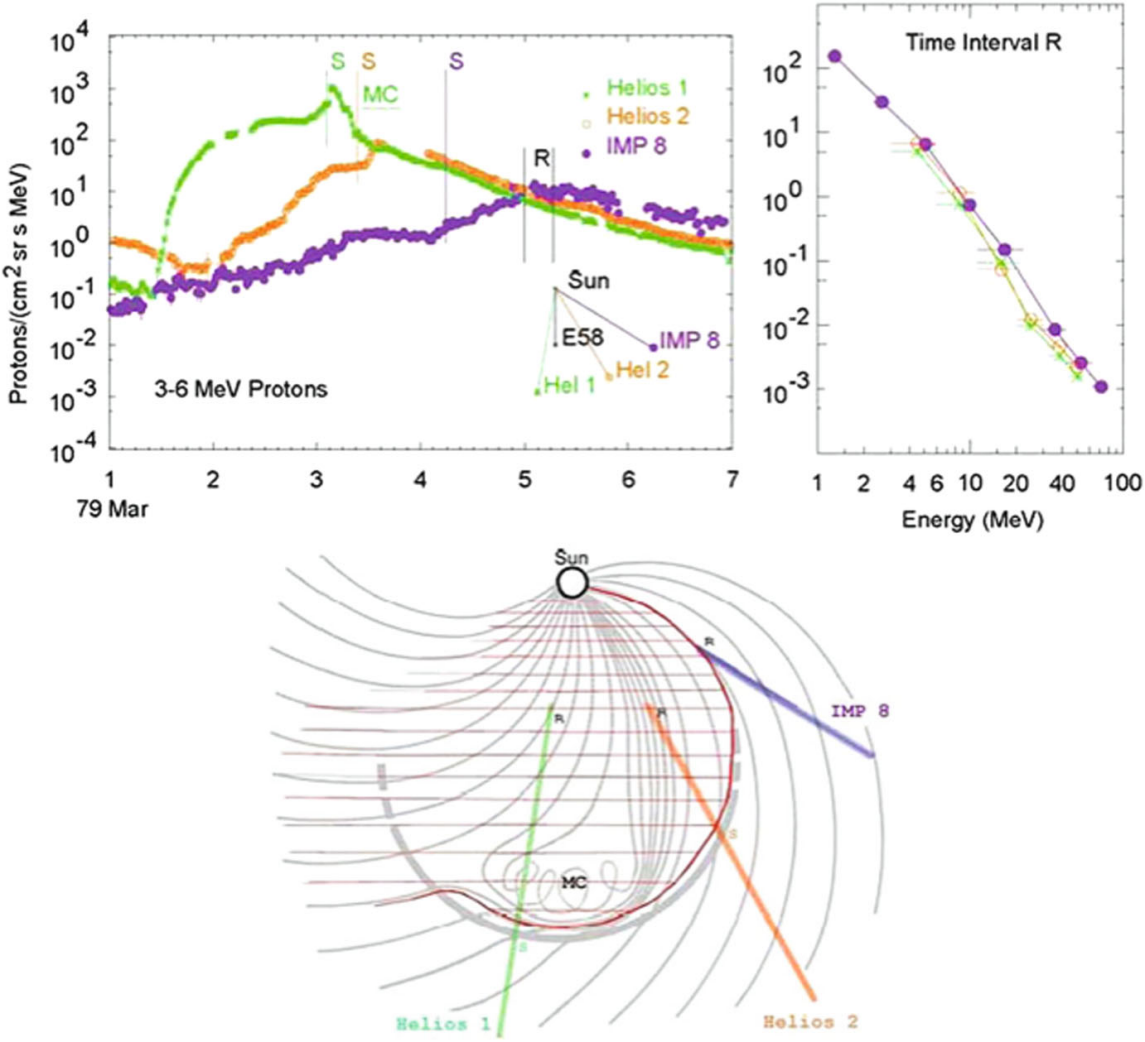
*Top left* Proton intensity-time profiles during the March 1, 1979 event at 3 s/c; ‘S’ represents the the time of shock passage at each s/c. *Top right* Energy spectra in the reservoir or the spectral invariant region behind the shock at time ‘R’. *Lower panel* Spacecraft trajectories through the the CME. Image reproduced with permission from Reames (2013), copyright by Springer

In Sect. 2.1, we discussed how the longitudinal spread of large SEP events allowed researchers in the 1990s to classify SEP events into two distinct types. Here we discuss in detail the differences in the SEP properties as observed at different vantage points. Figure 52 shows that Helios 1 encounters an SEP event near the central meridian and observes a peak in the 3–$6\,\mathrm{MeV}$ proton intensity near the time of shock passage. At the other two s/c, the proton intensities reach a peak after shock passage and then track those seen at Helios 1 for several days thereafter. This time interval has been termed a particle reservoir (also see McKibben 1972; Roelof et al. 1992; Roelof 2012a, b) and reflects a region where the intensities and energy spectra throughout much of the inner heliosphere (see Fig. 52: top right panel) at different azimuthal, radial, and latitudinal locations are nearly identical (e.g., McKibben 1972; Reames 2010; McKibben et al. 2003). These results indicate that only a small number of particles escape from and leak out of the reservoir, because if the ions were able to do so, then the SEP energy spectra in the reservoir would soften or steepen with time while the corresponding intensities would decay far more rapidly than is typically observed.

Key physical processes believed to play important roles in creating the reservoir effect during the decay phases of large SEP events include: (1) fine-scale mixing of open and closed coronal magnetic fields (Reames 2010; 2) energy-dependent transport and the inhibition of particle escape due to the presence of one or more preceding magnetic disturbances, such as magnetic clouds or ICMEs (Roelof et al. 1992; Roelof 2012a, b; Reames 2013); and (3) non-diffusive transport that includes effects such as convection and adiabatic deceleration (Lario 2010). Additionally, for SEP events where the low-energy portion of the spectrum slowly unfolds or steepens, reservoir theoretical concepts must include: (1) replenishment of lower-energy particles by continuous acceleration at the increasingly weakening CME shock, (2) preferential escape of high-rigidity or higher-energy particles, and (3) energy-dependence of perpendicular transport (Reames 2013).

### SEP observations at Helios and Ulysses

The Helios and Ulysses missions explored the inner heliosphere inside Earth orbit and at high latitudes inside ${\sim }5\,\mathrm{AU}$, providing new insights into the spatial distribution of SEP events. Kallenrode (1996) found that the ${\sim }5\,\mathrm{MeV}$ proton intensity increases associated with ${>}350$ transient IP shocks at Helios correlated with local and transit shock speeds and the angular distance between the s/c and the flare location; the highest intensities were observed close to the nose of the shock and decreased near the flanks, confirming the role of CME shock acceleration in large SEP events. Kallenrode (1997) studied the radial evolution of shock acceleration efficiency and found that CME shocks are more efficient at accelerating particles closer to the Sun, and that since this efficiency decreases with radial distance, the particles that are accelerated in IP space do not require significant energization and could originate from a pool of material that was accelerated earlier and closer to the Sun (also see Kallenrode et al. 1993).

Comparing SEP observations near the ecliptic plane with those observed by Ulysses at higher latitudes, McKibben et al. (2003) noted significant intensity increases at both locations regardless of their positions with respect to each other or to the location of the flare. The onsets at both locations during such multi-point SEP events were prompt; however, at the highest latitudes, the maximum intensities were somewhat lower and their rise was also slower. Onset anisotropies were field-aligned and directed outward at all latitudes, which indicated acceleration over a broad front or efficient perpendicular transport across the magnetic field lines. The SEP intensities at both locations then reached similar levels during the decay phases, which indicated that the spectral invariance region and reservoir effects were essentially three-dimensional in nature. Dalla et al. (2003a, (2003b) studied the differences between SEP onset times and times-to-maximum as a function of latitude and found that the delays at Ulysses were best organized by the difference in latitude between the associated flare and the s/c. Dalla et al. (2003b) concluded that the presence of a shock is not necessary for creating the near-equal intensities observed at Ulysses and near Earth during decay phases; these observations are better explained by diffusion across the main IMF.

### Intensities of CME-associated ESP events

**Fig. 53:**
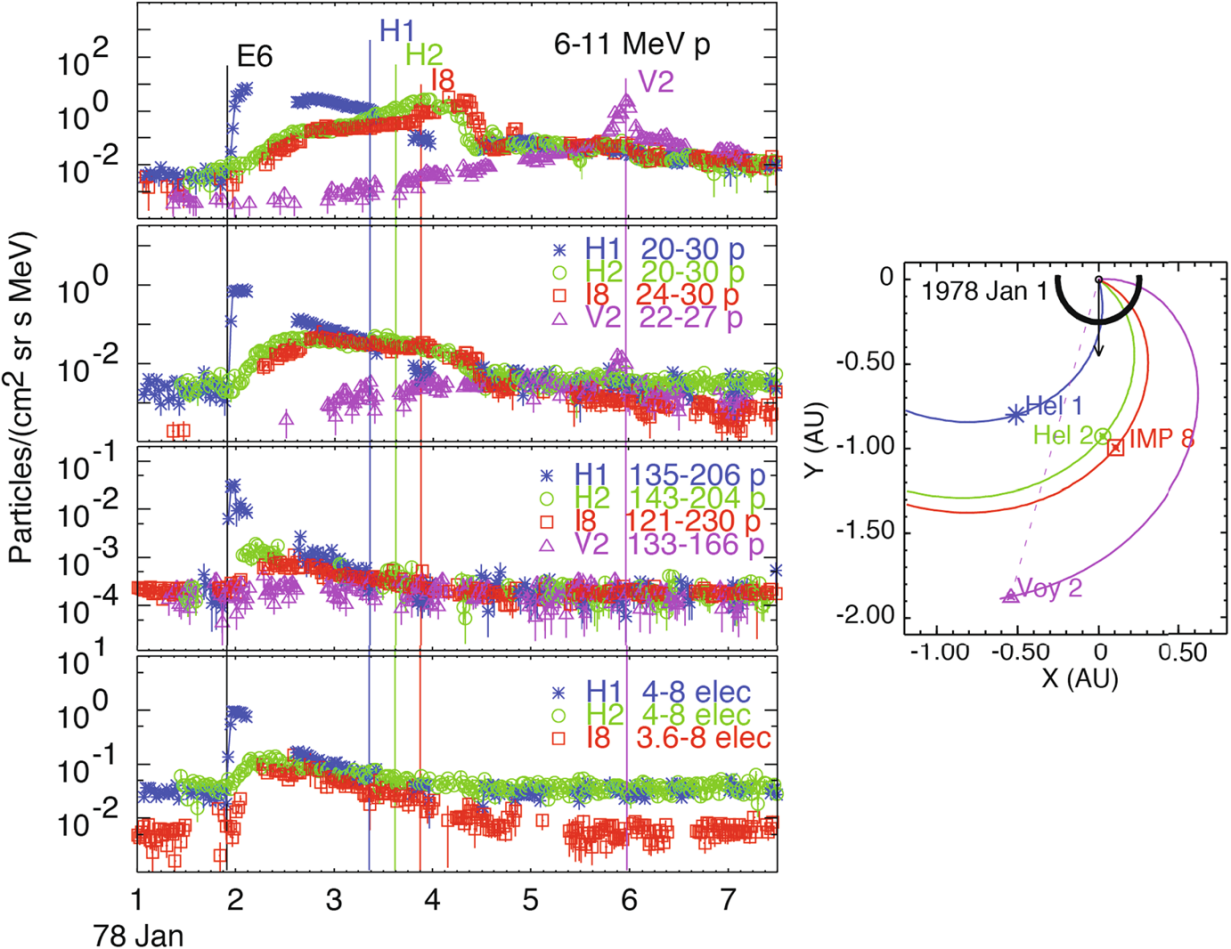
*Left* Proton and electron intensities in several energy ranges as measured by 4 s/c whose relative locations are shown at right during the January 1978 SEP event. *Vertical lines* show the time and longitude of the flare (E6) and the times of shock passage at each s/c. *Blue asterisks* represent data from Helios 1, *green circles* represent Helios 2 data, *red squares* show data from IMP 8, and *violet triangles* are Voyager 2 data. Image reproduced with permission from Reames et al. (2013), copyright by Springer

Figure 53 shows intensity-time profiles of $\sim$1–200 MeV protons and $\sim$3–8 MeV electrons during the January 1, 1978 SEP event as observed at four different s/c. Helios 2 and IMP 8 observe an SEP event near central meridian; the proton intensity peaks just after shock passage. For different energies, the particle reservoir is observed at different times; for electrons and high-energy protons, equal intensities at Helios 1, Helios 2, and IMP 8 are seen ${\sim }2$ days earlier than that for the lower-energy protons. Interestingly, the intensity peak at $\sim$6–11 MeV at Voyager 2 (located at 1.95 AU) is comparable to those seen at Helios 2 and IMP 8, which were located near or within ${\sim }1\,\mathrm{AU}$. This one case study is somewhat at odds with the large statistical survey of Kallenrode (1997), and indicates that this particular CME shock was still just as efficient at accelerating protons up to at least ${\sim }10\,\mathrm{MeV}$, all the way out to ${\sim }2\,\mathrm{AU}$.

### STEREO observations of SEP events

**Fig. 54:**
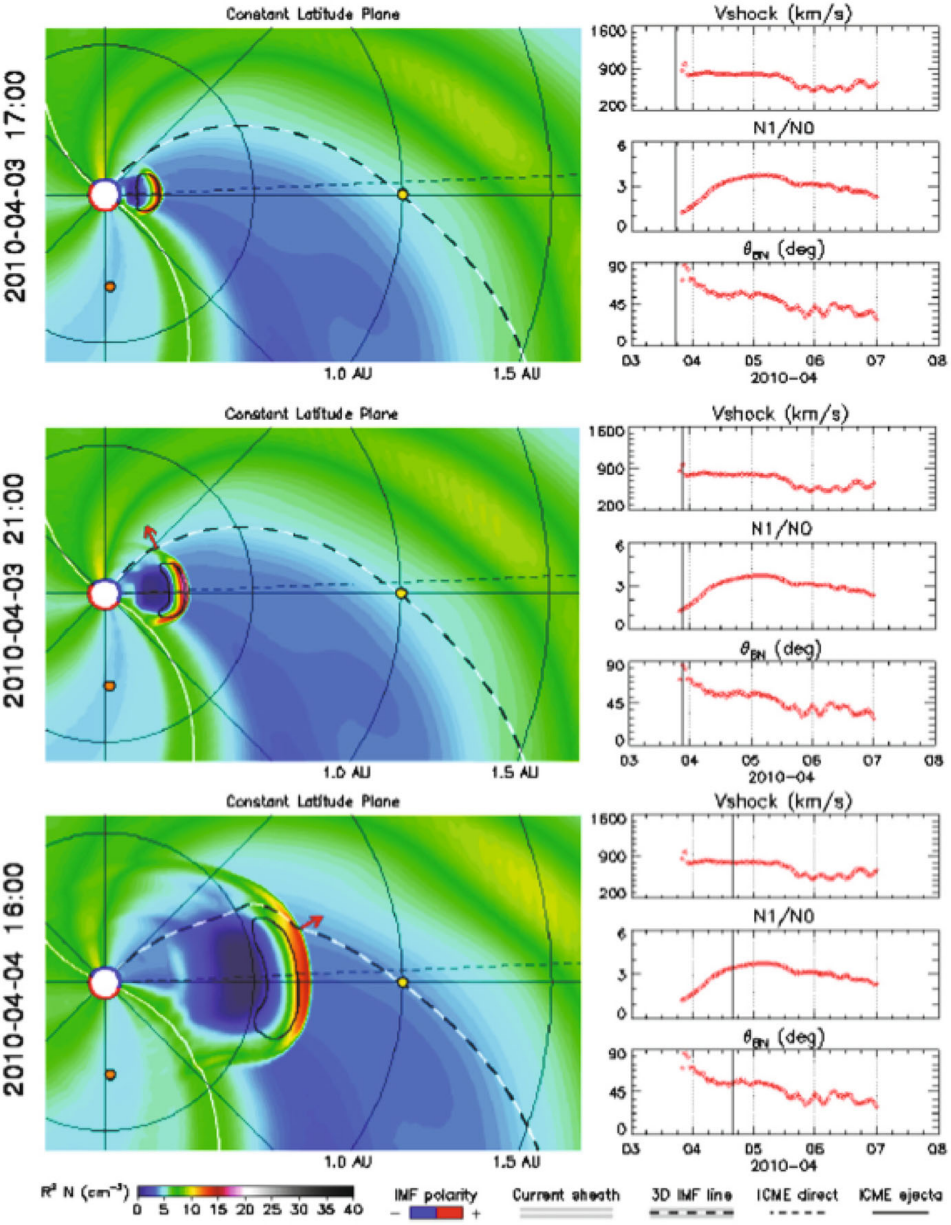
*Left* Contour plots of the simulated plasma radial speed showing the solar ecliptic plane from the solar north pole at 1700 UT on April 3, 2010 (*top*), at 2100 UT on April 03, 2010 (*middle*), and at 1600 UT on April 04, 2010 (*bottom*). *Black/white lines* indicate the magnetic field lines passing through the L1 s/c. *Right* Shock speed ($\mathrm{V}_{\mathrm{shock}}$), compression ratio (N1/N0), and the angle between the magnetic field lines threading the shock and the shock normal ($\theta _{BN}$) simulated along the magnetic field connected to L1. Image reproduced with permission from Rouillard et al. (2011), copyright by AAS

More recently, remote sensing and in-situ measurements from SoHO, STEREO, ACE, and Wind combined with state-of-the art modeling have provided new insights into the spatial distributions of SEP events. In particular, white light coronagraphs on STEREO and SoHO can image the temporal evolution of the spatial structure, speed, position, and acceleration of the CME and its shock (see Rouillard et al. 2011, 2012). These observations can then be compared with model results to infer CME shock properties in the corona and inner heliosphere (see Fig. 54). The Rouillard et al. studies combined ultraviolet and white-light images of the CME and the solar corona with a comprehensive study of velocity dispersion of energetic particles observed at STEREO and L1 and found that the delayed solar particle release times at STEREO and L1 are consistent either with: (a) the time required for the CME shock to reach field lines connected to the s/c, or (b) the time required ($\sim$30–40 min) for the CME to perturb the corona over a wide range of longitudes. These results established a direct association between the longitudinal extent of the SEP event in the heliosphere and that of either the CME shock or the disturbed corona due to the lateral extension of the CME.

### Large longitudinal spread of $^{3}$He-rich SEP events

While the specific topic of $^{3}$He-rich or impulsive SEP events will be covered in a subsequent review article, in this section we discuss some of the key recent multi-spacecraft observations of these smaller events that have essentially re-opened the broader debate about particle transport in the corona and interplanetary medium, and the role of meandering field lines in distributing particles over a larger range of longitudes than previously thought. It is generally understood that impulsive SEP events observed in-situ near Earth—characterized as being rich in $^{3}$He—are associated with solar flares occurring over a relatively narrow range of western-hemisphere longitudes on the Sun. Figure 5a shows a histogram of the source longitude of impulsive events observed at Earth (from Reames 1999). Physically, this is straightforward to understand. High-energy particles associated with a small, localized source on the Sun will move in the IMF, which is consistent with the well-known Parker spiral. Since particles tend to move more easily along the field than across it, the particles will essentially follow the Parker-spiral magnetic field. When following this field backwards from an observer near Earth to see where its footpoint is at the Sun, one finds that under typical solar wind conditions, the solar longitude of this footpoint is about $60^{\circ }$ west. This is roughly consistent with the histogram shown in Fig. 5a.

However, more recent observations have challenged this idea, noting that the longitudinal distribution of impulsive SEPs can be larger than originally thought. For example, Wibberenz and Cane (2006) analyzed high-energy electrons in individual SEP events observed at multiple s/c and found that their longitudinal distribution was larger than expected. This suggests particles may not adhere to the *mean* magnetic field as much as previously thought.

**Fig. 55:**
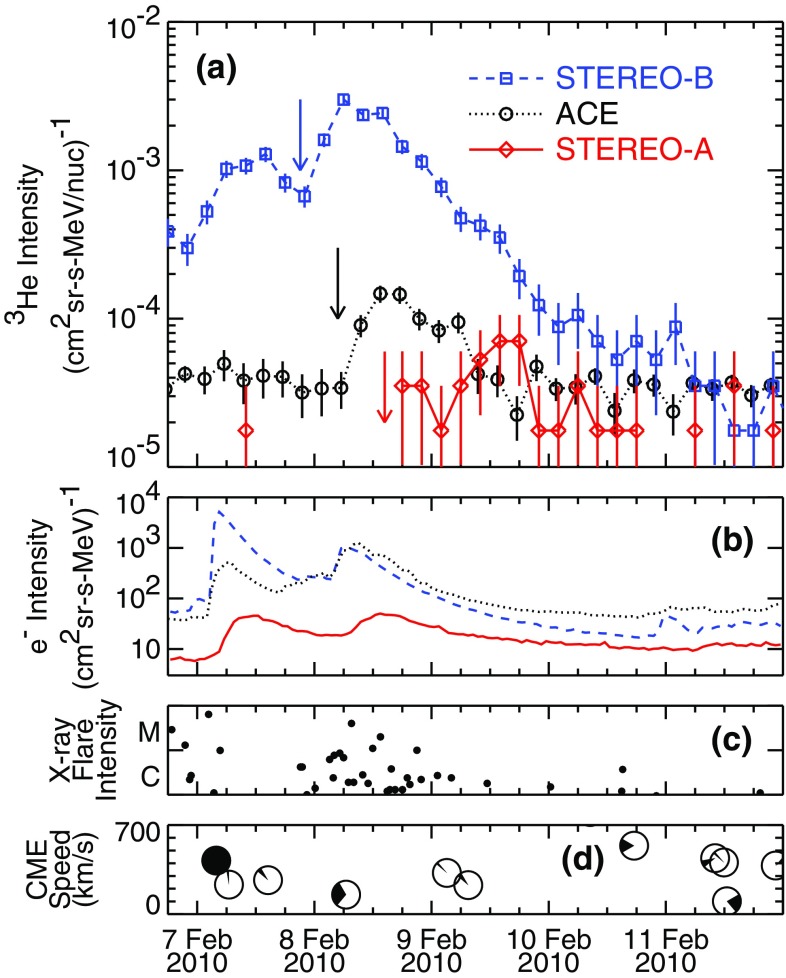
Observations of a single $^{3}$He-rich SEP event seen by three s/c widely separated in heliolongitude. A large solar active region, AR11045, was seen on the Sun at about the center of the solar disk when the $^{3}$He-rich SEP event was observed by all three s/c; *arrows* depict the delayed onset times of the event. Image reproduced with permission from Wiedenbeck et al. (2013), copyright by AAS

Wiedenbeck et al. (2013) analyzed several impulsive-flare-related SEP events observed by three different s/c which were separated widely in heliolongitude. This suggests an even greater longitudinal extent of such SEP events than that found by Wibberenz and Cane (2006). Figure 55a shows the 2.3–3.3 MeV/nucleon ${^3}$He intensity as a function of time, observed by three s/c. Figure 55b shows the 70–100 keV electron intensities measured by the three s/c during the same period. Figure 55c is a scatter plot of all X-ray flares seen during this time period, with each dot indicating the time and intensity (vertical axis) of the flare. Figure 55d shows the occurrence of CMEs during this period, with the vertical axis corresponding to the CME speed and each symbol representing the direction of propagation (for details, see Wiedenbeck et al. 2013).

Wiedenbeck et al. (2013) noted that, during the time period in which the SEP event was observed, there was a large active region on the Sun (AR1045) approximately at the center of the solar disk. This active region was presumably the source of the energetic particles. Using the observed solar wind speed, the authors also noted that a magnetic line of force whose point of origin was near zero solar longitude would cross 1 AU—the orbit shared by the three s/c that observed this event—at approximately the same location as STEREO B. In fact, as can be seen in the figure, the highest intensity is indeed seen by this s/c. ACE was about $70^{\circ }$ to the west of STEREO B during this time and also clearly observed the event. It is surprising, however, that even STEREO A, which was separated from STEREO B by more than $130^{\circ }$ in heliolongitude, observed an increase in the SEP ion intensity, although the intensity was clearly much smaller than that seen by the other two s/c. Note that two prominent impulsive electron events, both consistent with the AR11045 source region, were also seen by all three s/c, but only the second $^{3}$He-rich event was observed—probably due to a combination of lower intensities and instrument sensitivities at ACE and STEREO A.

Wiedenbeck et al. (2013) fit the resulting longitudinal distribution of the event-integrated $^{3}$He intensity seen by all three s/c to a Gaussian function whose maximum occurs at the longitude connecting 1 AU and the source (near STEREO B in this case). They found that the width of standard deviation of the functional fit was about $48^{\circ }$. This is considerably larger than the width inferred from the histogram shown in Fig. 5a, suggesting very efficient longitudinal transport of the ions associated with the SEP event. In addition, if all SEP ions and electrons are simultaneously and impulsively injected from the flaring source onto open field lines, then the longitudinal spread of these particles could pose a serious challenge for the generally accepted notion of scatter-free transport in impulsive SEPs. Giacalone and Jokipii (2012) used a diffusive-transport model for the propagation of energetic ions in interplanetary space, which took into account the transport both along and across the local Parker-spiral magnetic field and the longitudinal motion of the magnetic lines of forces rooted at the Sun as it rotates. In this model, such effects lead to substantial longitudinal transport of the particles, such that even spacecraft separated by as much as $180^\circ$ or more may observe impulsive SEP events associated with compact solar flares.

### Longitudinal distributions of large gradual SEP events

Dresing et al. (2012) analyzed a CME shock-related SEP event that revealed an even larger longitudinal extent of the energetic particle intensities than the ones discussed above. The solar eruption that may have caused the particle event at 1 AU occurred on the far side of the Sun, as observed from Earth. Energetic electrons were seen essentially all the way around the Sun. This event was likely larger than the ones discussed above as it also involved a CME shock. These authors also presented results from a model of SEP transport that included cross-field diffusion. They considered two possible explanations: (1) the event involved an unusually long “injection” time; and (2) there was an unusually large amount of cross-field diffusion of particles in interplanetary space. They argued that (1) was not likely because there was no evidence of an extended injection, and concluded that (2) was the most likely scenario, but this required an unrealistically large value for the ratio of the perpendicular-to-parallel diffusion coefficient of ${\approx }0.3$.

**Fig. 56:**
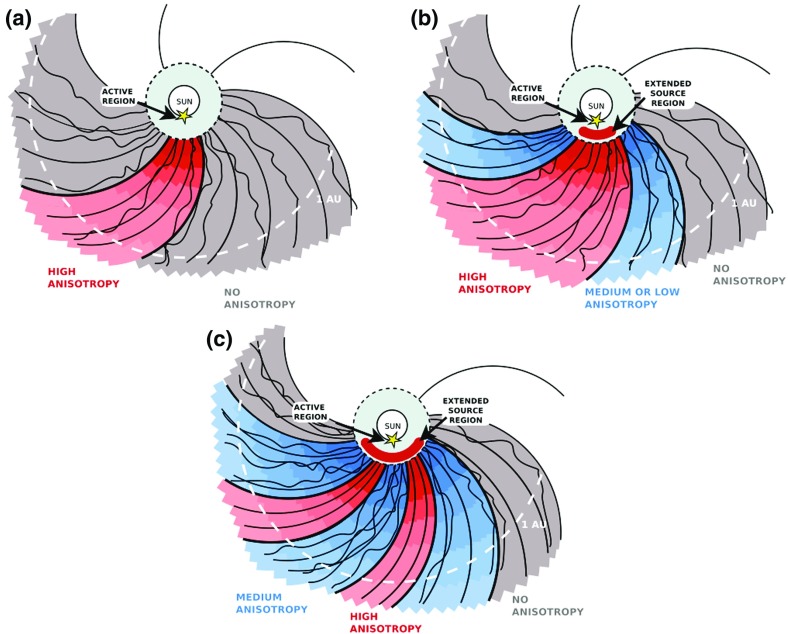
Three possible causes of the large longitudinal spread of SEP events as observed by the STEREO s/c. Image reproduced with permission from Dresing et al. (2014), copyright by ESO

Surveying the 55–105 keV electron anisotropies during 21 large SEP events using the STEREO s/c as they reached a separation of up to ${\sim }180^{\circ }$ in azimuth, Dresing et al. (2014) found that the events could be divided into three distinct groups (see Fig. 56): (1) a small source region (flare) injects particles in a narrow region, resulting in large particle anisotropies at well-connected s/c and near-isotropy at widely separated locations; (2) an extended source region injects SEPs into the IP medium over a broader region near the Sun, resulting in large anisotropies at locations connected to the extent of the source region and in decreasing anisotropies at widely separated s/c; and (3) particles injected from an extended source region near the Sun undergo substantial cross-field transport in the IP medium or encounter earlier CMEs or other IP structures. The third scenario results in complex anisotropy features: large anisotropies at less well-connected locations indicate that the SEPs experienced little or no scattering or did not encounter structures that inhibit transport, while smaller anisotropies at well-connected s/c indicate the opposite. The precise mechanisms are not discernible from these observations.

Rouillard et al. (2012) also addressed the large longitudinal spread of SEP events for an event that occurred on March 21, 2011. They combined several data sets, including remote-sensing observations, to relate the event at the Sun to the SEP event seen at 1 AU. Among their key findings was that the solar corona was perturbed by the CME over a range in longitudes roughly corresponding to the longitudinal separation of s/c at 1 AU. Rouillard et al. (2012) argued that their results are consistent with a scenario where the acceleration process itself occurs over a wide range of longitudes rather than the alternative scenario in which particles that are accelerated in a confined location undergo substantial lateral transport in the corona or interplanetary medium before being injected at a distant longitude.

**Fig. 57:**
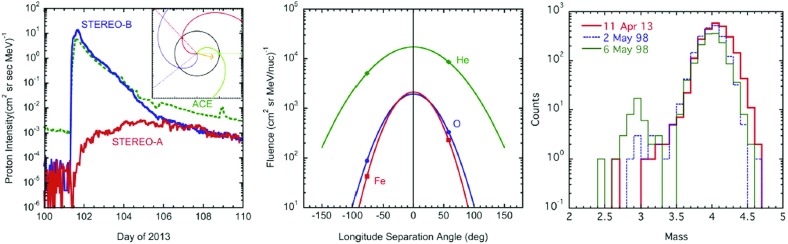
*Left* 10–30 MeV proton intensities for the April 11, 2013 event from STEREO A, ACE, and STEREO B. *Inset* shows s/c locations relative to the flare. *Center* 12–33 MeV/nucleon event-integrated fluences of He, O, and Fe versus s/c longitude; STEREO B is at $58^{\circ }$ and ACE is at $-77^{\circ }$. *Right* ACE/SIS He mass histograms compared with those measured during two Fe-rich SEP events in solar cycle 23. Image from *ACE News #170*, adapted from Cohen et al. (2014)

Lario et al. (2013) fitted the longitudinal distributions of peak proton intensities in 35 multi-s/c large SEP events with a Gaussian distribution that is offset by $\phi _{0}$ from the flare longitude and the magnetic footpoint of the observing s/c: $j=j_{0}\exp [-(\phi -\phi _{0})^{2}/2\sigma ^{2}], \phi$ is the separation between the flare and the magnetic footpoint of the s/c, and $\sigma$ is the width of the distribution. They found an average offset of $\phi _{0}=-12^{\circ }\,\pm \,3^{\circ }$ (also see Lario et al. 2006). Cohen et al. (2014) studied the April 11, 2013, large Fe-rich SEP event—the first of solar cycle 24—and noted that the high, but longitudinally variable, Fe/O ratios and simultaneously low $^{3}$He/$^{4}$He ratios were not expected from either direct flare contributions or re-acceleration of flare suprathermals (Fig. 57, Sect. 2.6).

Following Rouillard et al. (2012), Lario et al. (2014) compared the estimated release time of SEPs observed by multiple s/c during the 2013 April 11 event with the arrival time of the CME-associated structures at the field line footpoints connecting each s/c to the Sun. They concluded that the arrival of the EUV wave and CME-driven shock at the STEREO B footpoint is consistent with the corresponding SPR time, but the EUV wave never reached the field-line footpoints that connect near-Earth observers with the Sun, and that the intense SEPs observed near Earth are most likely produced by the higher altitude, western portion of the CME-driven shock that did not create EUV signatures on the solar surface. Based on their results for the April 2013 SEP event, and in contrast with the Rouillard et al. (2012) study, Lario et al. (2014) concluded that the angular extents of EUV waves cannot serve as proxies for solar surface phenomena that accelerate and inject SEPs over broad ranges of longitudes.

We note, however, that the Lario et al. (2014) conclusion is in stark contrast with several other studies that explored links between the lateral or coronal expansions of EUV waves and SEP proton events (e.g., Nitta et al. 2012; Park et al. 2013, 2015; Miteva et al. 2014). In particular, Park et al. (2015) reported that the SEP peak fluxes increased and the SEP spectral indices became harder with the EUV wave speeds, while Gómez-Herrero et al. (2015) found a good correlation between the EUV wave arrival times at the connecting magnetic footpoints and the proton onset times during the November 3, 2011 multi-spacecraft SEP event. This indicates that higher SEP fluxes, harder spectra, and direct injection of SEPs onto well-connected IMF lines are associated with lateral expansions of CME-driven shocks in the low corona, and may therefore be responsible for the rapid longitudinal spread as observed at vastly distributed s/c in many SEP events.

The above conclusions are further supported by Gopalswamy et al. (2014), who reported that only those fast CMEs that are magnetically connected to the strongest part of the CME shock, i.e., the nose, are associated with GLEs near Earth, and that conversely, GeV-proton-producing fast CMEs may not result in a GLE event because of poor latitudinal connectivity between the nose and the Earth. Other studies that also support the importance of magnetic connectivity have shown that key SEP properties, such as enhancements in the Fe/O ratio and the Q/M-ordering of the heavy ion abundances, depend on the proximity of active regions (AR) to the magnetic footpoints that link the source regions to s/c near L1, which indicates that the nearby ARs may occassionally supply suprathermal seed ions to the CME-driven shocks (Ko et al. 2013). In contrast, Shen et al. (2006) and Kahler et al. (2014), found that SEP proton intensities are not well correlated with any property (e.g., proximity, relative location) of coronal holes, which implies that SEP events can be routinely produced in fast solar wind source regions and that the associated CMEs need not be significantly faster.

It is also worthwhile noting that many of the above studies also found that the onsets and properties of relativistic electrons during SEP events were essentially uncorrelated or independent of the lateral expansion times or properties of EUV waves, suggesting that transport processes in interplanetary space, including cross-field diffusion, may also play an important role in providing SEPs access to a broad range of helio-longitudes (e.g., Miteva et al. 2014; Park et al. 2015). Other factors that may also play a role in distributing SEP events longitudinally include the large-scale IMF configuration inside interplanetary CMEs or magnetic clouds (e.g., Kahler and Vourlidas 2013; Kahler et al. 2014; Miteva et al. 2013), and the relative strength of the CME shock, which depends on the local Alfvén speed, rather than the actual speed of the CME (e.g., Shen et al. 2007; Gopalswamy et al. 2014).

Thus it appears that a combination of physical processes can disperse high-energy charged particles in interplanetary space and cause their large longitudinal spread, both from compact solar sources such as flares and large-scale phenomena such as CME-driven shocks. These include lateral expansion of EUV waves, proximity of magnetic footpoints to nearby ARs, solar rotation and transport—including pitch-angle scattering, cross-field diffusion, motion along the large-scale IMF and meandering magnetic field lines, and adiabatic energy losses—near the Sun and in interplanetary space. It is presently not known whether most of the transport-related effects take place near the Sun, and if the particles simply diffuse outward from there, or whether these effects occur during particles’ transit from the Sun to Earth. Transport across the nominal Parker spiral magnetic field is almost certainly enhanced by the meandering of magnetic field lines associated with large-scale turbulence (e.g., Jokipii and Parker 1968; Giacalone et al. 2000a), but it is not clear whether this is the dominant effect that is responsible for the observed large longitudinal spread of SEPs. However, we note that, recent numerical models that solve equations for energetic particle transport using reasonable transport parameters find qualitatively good agreement with the observations, even for a point-source impulsive release of particles at the Sun (Giacalone and Jokipii 2012). Thus it is particularly important to know the spatial and temporal extent of the source region.

### Flux dropouts

**Fig. 58:**
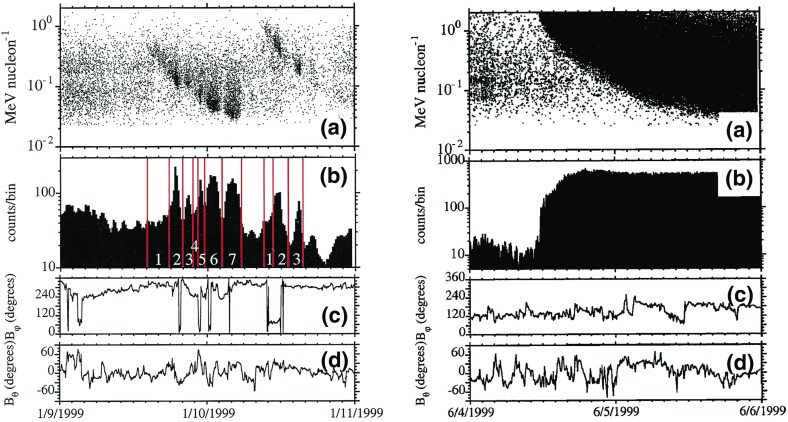
Two SEP events displayed such that each dot in the top panels represents the detection of an ion by ACE/ULEIS. SEP velocity dispersion, and the presence (*left*) or absence (*right*) of flux dropouts are easily identified. Image reproduced with permission from Mazur et al. (2000), copyright by ESO

As we have just discussed, impulsive SEP events, quite surprisingly, can be dispersed over a wide range in solar longitudes, which suggests that charged particles are transported across magnetic fields more easily than previously thought. However, many impulsive SEP events exhibit features that suggest quite the opposite. Mazur et al. (2000) first reported the phenomena now known as “dropout” events. During many impulsive SEP events, detectors at 1 AU first see the highest energy particles associated with the event simply because they are the fastest and reach the detector the soonest. Lower energy particles arrive later. By analyzing the ACE/ULEIS data, Mazur et al. (2000) were able to plot each particle detection as a function of time and revealed this velocity dispersion in a unique way. The event shows up essentially as a backwards-shaped comma feature (see Fig. 58). During a subset of these events, intermittent depletions, or “flickering” on and off of the particle intensity, was also observed. This can clearly be seen in the left panel of the figure. This flickering on and off of the SEP intensity is known as SEP flux dropouts.

**Fig. 59:**
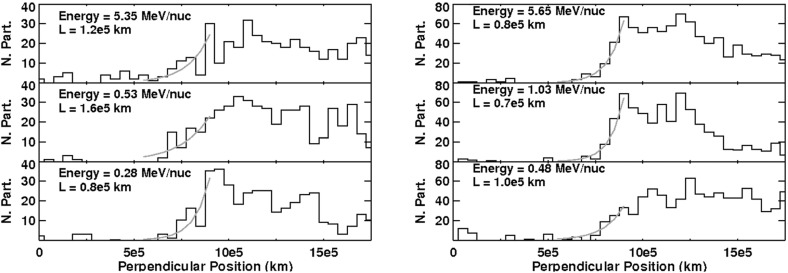
Analysis of ACE/ULEIS observations of the edges of impulsive SEP dropout events. *Left* Superposition of dropout edges plotted in units of diffusion length, L (km), which take into account the convection of flux tubes that passed the s/c with the observed solar wind speed. *Right* Superposition of six dropouts observed during the impulsive SEP events that occurred on DOY 225 of 2000. *Each panel* shows three different energies. Image reproduced with permission from Chollet and Giacalone (2011), copyright by AAS

Dropouts are likely caused by the s/c passing through alternately filled and empty “tubes” of particles (Giacalone et al. 2000a). The tubes in this scenario refer to magnetic lines of force to which charged particles adhere rather closely. The magnetic connection between the s/c and source (impulsive flare) determines which field lines are populated with particles and which are not. In fact, Mazur et al. (2000) related the timing of the dropouts to the characteristic scale of interplanetary magnetic-field turbulence, showing that the observations are generally consistent with this picture. This explanation requires the particles to be essentially trapped inside the flux tubes, because if there were significant leakage off of the field lines, then the particles would fill in the regions between them, smoothing out the particle flux to the point where dropouts would not occur. Chollet and Giacalone (2011) analyzed the edges of the intermittent drops in intensity by fitting the exponential in Eq. (2), and found that the gradients are indeed very large. Here, *L* is the fitted exponential length of the intensity drop, *x* is the distance from the edge of the dropout, and *N*(*x*) is the number of particles in a given energy bin. Results of this analysis are shown in Fig. 59. Clearly, the drop in intensity is independent of energy and occurs over a scale of the same order as the particle gyro-radii, suggesting that the particles undergo very little scattering off of the field lines during the time they are transported from the Sun to Earth.

2 $$\begin{aligned} N(x) = A\exp (-x/L) \end{aligned}$$

We note that numerical simulations by Giacalone et al. (2000a) support the picture of alternately filled and empty flux tubes described above. These simulations showed that the dropouts occur when the spatial size of the source is of the same order, or possibly smaller, than the typical scale size of solar supergranulation, which is thought to be responsible for the large-scale field line meandering (e.g., Jokipii and Parker 1968; Giacalone et al. 2000a; Giacalone and Jokipii 2004). The spatial size of the supergranulation cells is ${\sim }4 \times 10^{4}\,\mathrm{km}$ (Giacalone et al. 2000a), while the average scale size of a typical filled flux tube is ${\sim }4.7 \times 10^{6}\,\mathrm{km}$ or ${\sim }0.03\,\mathrm{AU}$ (Mazur et al. 2000). This implies that the dropout events likely originate from small localized regions on the Sun. Other interpretations of the impulsive-SEP-dropout phenomena invoke: (1) temporary trapping of particles within small-scale structures followed by rapid magnetic-field line diffusion (e.g., Ruffolo et al. 2003; Chuychai et al. 2007; 2) interplanetary turbulence that allows the magnetic-field lines to meander and spread out independently of supergranulation (Ragot 2012; Laitinen et al. 2013b; 3) stochastic nature of the time-varying magnetic connectivity to the source regions in the presence of magnetic turbulence (Ruffolo and Matthaeus 2015).

Chollet et al. (2009) used the Giacalone et al. (2000a) interpretation, along with in-situ observations of electrons and ions, to infer the source locations (longitude and latitude in the solar atmosphere) of impulsive SEP events. They found several events in which the ${<}1300\,\mathrm{eV}$ suprathermal electrons and the ${<}2\,\mathrm{MeV/nucleon}$ energetic ions, i.e., species with similar speeds, exhibit simultaneous, dispersionless intensity dropouts, which indicates that the corresponding source regions for these events must overlap, at least partially. In the same survey, they also identified several other events where the ion and electron dropouts were not coincident, which indicates that the corresponding source locations for these events are distinct, consistent with X-ray and $\gamma$-ray observations of solar flare events (e.g., Lin et al. 2003).

Indeed, the distinct electron and ion source locations may also be related to another important unresolved issue—why do some impulsive electron events show little evidence of dropouts? It is tempting to attribute this discrepancy to the fact that the flux dropouts during isolated $^{3}$He-rich SEP events are observed when the energies of individual ions are pulse-height-analyzed by instruments such as ACE/ULEIS, whereas the energetic electron measurements are typically obtained by single parameter solid state detector-based instruments such as ACE/EPAM, which measure the energy deposited by electrons in broad energy bands (Gold et al. 1998). Upon closer examination, however, the ${\sim }3$-h sub-intervals used by Mazur et al. (2000) to characterize the ion count rate flux dropouts shown in Fig. 58 should also be adequate for observing electron flux dropouts, even accounting for the broader energy intervals.

The right panel of Fig. 58 shows a large SEP event. In this case no dropouts are observed. There are two possible reasons for this: (1) there is so much scattering during transport between the Sun and Earth that any dropouts have been smoothed out; and (2) the source of particles for this event had a particularly large spatial extent so that the magnetic field lines through which the s/c moves all connect to the source. These are discussed further in Giacalone et al. (2000a, (2000b).

## Theoretical concepts

Understanding the origins of SEPs is a considerable theoretical challenge. On the one hand, there is a general consensus that acceleration at shock waves—presumably driven by CMEs and also possibly in the vicinity of solar flares—is responsible for producing the vast majority of particles seen in SEP events at Earth (cf. Reames 1999). However, there remain significant gaps in our understanding of how exactly this happens. Moreover, it is typical to treat the acceleration of SEPs and their transport away from the acceleration site as two separate problems, even though the basic physics involved in acceleration and propagation are intrinsically linked. In fact, in most acceleration mechanisms that have been proposed, charged particles must be sufficiently confined in the acceleration region to achieve high energies; thus it is important to understand the transport of particles in the vicinity of acceleration sites.

Much of the theoretical basis for our understanding of the physics of energetic-charged particle transport^1^ in the solar corona and solar wind was first discussed in a seminal paper by Parker (1965). Although Parker’s analysis made the approximation that the distributions were quasi-isotropic and averaged over the magnetic gyro-phase of the particles, this work laid the theoretical foundation for many of the numerical and analytic approaches used today. The resulting well-known, cosmic-ray transport equation—sometimes simply called the “Parker equation”—is extremely useful, even though it does not apply in some important situations that we will discuss below. In fact, this equation contains the basic physics of particle acceleration at shocks as well. The equation, despite its relative simplicity, is not easy to solve except for very simple geometries, which are not suitable for one-to-one comparisons with observations of SEPs. The modern approach is to solve the equation—or extensions of it that include pitch-angle information—using a computer.

Because of its importance to the theoretical understanding of SEPs, this review will start with a discussion of basic SEP transport processes in the context of the Parker equation. Then we will discuss possible SEP acceleration mechanisms, focusing primarily on acceleration by CME-driven shock waves (for a similar discussion, see Lee et al. 2012).

### The physics of charged-particle transport

The forces that govern SEP transport are dominated by electric and magnetic fields in space. The magnetic field, $\mathbf{B}$, we are most interested in is that which originates at the Sun and is carried outward by the nearly radially expanding solar wind moving with velocity, $\mathbf{U}$. Since SEPs move very fast compared to the typical characteristic wave speeds of the plasma, it is commonly assumed that the electric field—in the frame of reference fixed with respect to the Sun—is simply $-\mathbf{U}\times \mathbf{B}/c$, which comes from assuming there is *no* electric field in the frame moving with the plasma. Of course, other electric fields can be included, depending on the particular problem of interest, most notably near sites of magnetic reconnection. Other forces are usually neglected, since it can be shown they are extremely small in most problems. Also, it is important to note that SEPs and other high-energy charged particles suffer few particle-particle collisions, because the space through which they move is extremely tenuous. In fact, it is for this reason that SEPs exist, since such collisions would act rapidly to thermalize the distribution.

SEPs can usually be treated as test particles, since their number density is small compared to the typical number density of the solar wind. The energy density is also typically a small fraction of the solar wind energy density. However, there may be important situations where this is not true, and we must consider a self-consistent treatment of the energetic particles and plasma dynamics that includes SEP effects on the background plasma (e.g., Russell et al. 2013). Besides SEPs, there are likely other phenomena where this is important, such as charged particles accelerated at the solar wind termination shock (e.g., Decker et al. 2008; Florinski et al. 2009), and cosmic rays accelerated by blast waves associated with supernovae (e.g., Lucek and Bell 2000).

SEPs also offer insights into the physical processes involved in their acceleration and transport in space. The equations of motion, in fact, can be cast in a form that is similar to the radiative transfer equation describing the distribution of photons used in remote-sensing observations of distant sources. Thus, SEPs can be used to infer properties of remote regions of space by using in-situ observations combined with solutions to the transport equations.

#### The diffusive approximation: the Parker transport equation

The Parker equation, which governs the spatial, temporal and momentum evolution of a distribution of energetic charged particles, is given by Parker (1965):

3 $$\begin{aligned} {\partial f \over \partial t} = {\partial \over \partial x_i} \left[ \kappa _{ij} {\partial f \over \partial x_j}\right] - U_i {\partial f \over \partial x_i} + {p \over 3} {\partial U_i \over \partial x_i} \left[ {\partial f \over \partial p} \right] + \mathrm{Sources } - \mathrm{Losses}. \end{aligned}$$

where *f* is the phase-space distribution function of test particles as a function of the magnitude of the particle momentum, *p*, position, $x_i$, and time, *t*; $U_i$ is the bulk plasma velocity; and $\kappa _{ij}$ is the diffusion tensor. We note that the distribution function is related to the differential intensity, ${ dJ}/{ dE}$, which is commonly used in representations of SEP energy spectra, by ${ dJ/dE} = p^2f$.

The Parker equation averages over the pitch and phase angles of charged particle distributions—thus the equation is only strictly valid when the SEP distribution is nearly isotropic. Spacecraft observations of SEP events usually reveal that the first-arriving particles from large SEP events are not isotropic—distributions are beam-like and aligned mostly along the magnetic field—so the Parker equation is not suitable to describe these particles, and extensions of the equation must be considered. Nonetheless, after a few hours, many SEP events are observed to be nearly isotropic. This is especially true after the passage of shock waves associated with CMEs. Thus, the Parker equation is appropriate to model the intensity and spectra of SEPs for later times. We note that the assumption of isotropy is the only significant approximation in the Parker equation; thus, provided that the observed distributions are isotropic, the Parker equation is valid. This equation is widely used to model galactic cosmic-ray transport in the heliosphere and interstellar space, as well as the propagation of SEPs throughout the heliosphere. The Parker equation can also be used to solve for particle acceleration at shocks, as we discuss further below. For an expanded discussion of the Parker equation, see Giacalone (2010).

The Parker equation describes four main transport processes. These include: spatial diffusion due to the scattering of particles by fluctuations in the IMF associated with magnetic-field turbulence, advection with the solar wind, energy change, and drifts, such as gradient and curvature drifts due to variations in the large-scale heliospheric magnetic field. Energy change results from the particles moving against any electric field that is present, i.e., for a particle of mass *m* and charge *q* moving with velocity $\mathbf{w}$ and magnitude of momentum *p*, we have

4 $$\begin{aligned} {d\over dt}\bigg ({p^2\over 2m}\bigg ) = q\mathbf{w}\cdot \mathbf{E}. \end{aligned}$$

For the most commonly considered case—that of the motional electric field ($\mathbf{E}=-\mathbf{U}\times \mathbf{B}/c$) for a nearly isotropic distribution of particles, it can be demonstrated (see Jokipii 2012) that this reduces to the remarkably simple form:

5 $$\begin{aligned} {dp\over dt} = {p\over 3}\nabla \cdot \mathbf{U}, \end{aligned}$$

which is directly related to a term appearing in the Parker equation. This last form does not explicitly contain the electric field which ultimately gives rise to the particles’ energy change, but any changes in energy resulting from momentum diffusion can be added, if needed, although in many cases in the heliosphere this process is too slow to be important.

The energy change term in the Parker equation is proportional to the divergence in the bulk plasma flow velocity. The magnitude of this term can be quite large when the plasma undergoes a compression, such as at a shock wave, where there is a significant acceleration of the charged particles. Particle acceleration at shocks is discussed in detail in Sect. 7.2. However, this term can also lead to energy loss. In fact, for a purely radial solar wind moving with constant speed, $V_w, \nabla \cdot \mathbf{U}=2V_w/r$, which is finite and positive. This leads to the charged particles losing energy. This “adiabatic cooling” occurs for *any* charged particle moving in a constant, radial solar wind. This effect is especially important for SEPs that are accelerated close to the Sun, where adiabatic cooling can be quite large. The cooling time scale at 1 AU, for a typical value of $V_w=4\times 10^7\,\mathrm{cm/s}$, is about 6.5 days.

#### The spatial diffusion tensor

The diffusion tensor appearing in Parker’s Eq. (3), $\kappa _{ij}$, can be separated into components across the magnetic field, $\kappa _\perp$, and along it, $\kappa _\parallel$, where each are scalar functions of position and momentum. The diffusion tensor also includes particle drifts, since the form of the transport equation used does not explicitly separate them, which is often done in other treatments of cosmic-ray transport. The full tensor can be written (cf. Jokipii 1971; Giacalone 1998) as:

6 $$\begin{aligned} \kappa _{ij}=\kappa _\perp \delta _{ij}-{(\kappa _\perp -\kappa _\parallel )B_iB_j\over B^2}+\epsilon _{ijk}\kappa _A{B_k\over B}, \end{aligned}$$

where $B_i$ is the magnetic field vector, $\delta _{ij}$ is the Kronecker delta function, and $\epsilon _{ijk}$ is the Levi–Civita tensor. Determining $\kappa _\parallel$ and $\kappa _\perp$ has been the subject of considerable study and is discussed further below.

The antisymmetric diffusion coefficient appearing in Eq. (6) can be explicitly related to drifts caused by large-scale variations in the magnetic field, including those associated with its gradients and curvature of lines of force. By inserting the diffusion tensor into Eq. (3) and separating out the antisymmetric components, one obtains a new term given by, $\mathbf{V}_d\cdot \nabla f$, where $\mathbf{V}_d$ is the drift velocity. It can be shown rigorously that, by averaging the guiding-center drift velocity for any given charged particle (e.g., Northrop 1963) over an isotropic distribution, one obtains the following expression for the drift velocity: $\mathbf{V}_d = (cmw^2/q)\nabla \times (\mathbf{B}/B^2)$ (following Isenberg and Jokipii 1979, where *w* is the magnitude of the particle velocity). This term, representing charged-particle drifts, is commonly included in models of galactic cosmic-ray transport in the solar system. Such drift effects give rise to the 22-year cosmic-ray modulation cycle (Jokipii and Thomas 1981), but are often neglected in SEP transport. It can also be shown that the antisymmetric diffusion coefficient for most problems of interest, including GCRs, ACRs, and SEPs, can be written as $\kappa _A=(1/3)wr_g$, where $r_g$ is the ion Larmor radius (e.g., Giacalone 1999). The approximation only begins to break down for extremely turbulent magnetic fields.

It is generally accepted that drifts have important effects on GCRs, but have negligible effects on SEPs. Although it can be shown quantitatively that the magnitude of the drift term compared to the diffusion term is indeed small for the majority of SEPs, drifts can actually produce noticeable effects. For example, Jokipii (1970) showed that gradient and curvature drifts lead to a different timing in the peak intensities of electron and proton events, with ions lagging the electrons by up to several hours. Drifts are also likely to be more important at higher SEP energies (e.g., Marsh et al. 2013; Dalla et al. 2013).

As was discussed in Sect. 2.8, Mason et al. (2006) found that, in many SEP events observed at 1 AU, the time intensity profiles of O and Fe were much more similar to each other when compared at a “scaled” energy than at the same energy per nucleon, especially during the period when the intensities rise to a maximum. In particular, the time-intensity profile of 546 keV/nucleon O was nearly identical to that of 273 keV/nucleon Fe (and similarly for other energy pairs). These authors suggested that, although the matching time-intensity profiles were for species having a different energy per nucleon, the diffusion coefficient associated with their transport was the same. This can be understood in terms of the Parker equation if drifts are neglected and the particle source is a power law with each species having the same spectral exponent. For such a situation, the Parker equation is identical for the two different species. This means that the time-intensity distribution will be the same. This would change if drifts—which depend explicitly on speed, charge, and mass—were important. These observations provide an important constraint on the underlying acceleration mechanism and the transport process. They also clearly have important implications for the observed variability of the Fe/O ratio. In a follow-up study, Mason et al. (2012) employed a transport model that assumed, for simplicity, that all energetic particles were released impulsively at the Sun. Their model results showed reasonable agreement with the observations, but did not consider the effect of a continuous acceleration at a propagating shock, which clearly must also be occurring in these events.

#### Parallel diffusion

Charged particles execute a helical motion about a center of gyration, known as the gyro-center. In situations where the field is relatively smooth compared to the radius of this gyration, the particle moves along the field for many gyro-periods before it ultimately scatters when it encounters a fluctuation whose scale is comparable to its gyro-radius. The average distance between many such scatters is known as the parallel mean free path, $\lambda _\parallel$, and the average time between them is the scattering time, $\tau$. It is important that this *not* be confused with motion normal to the magnetic field, which is more appropriately discussed in terms of the spatial diffusion coefficient, discussed below, rather than a mean free path.

The cause of this scattering in the case of SEPs is fluctuations in the IMF. The IMF power spectrum has an approximate power-law dependence on wavenumber between the scales corresponding to the coherence scale measured to be about 0.01 AU for the random component of the IMF (Jokipii and Coleman 1968) and solar wind plasma turbulence (Jokipii and Hollweg 1970; Intriligator and Wolfe 1970), and the dissipation scale at about the thermal ion gyro-radius, about 100 km or so (Leamon et al. 1999). The spectral slope in this range, known as the inertial range, is $-5/3$, consistent with Kolmogorov’s theory for turbulence in an incompressible fluid (Kolmogorov 1941). The corresponding range of proton energies whose gyro-radii (in a 5 nT magnetic field) are comparable with the range in scales from the coherence scale to the dissipation scale is about 1 GeV to 1 keV, which covers the energy range of nearly all SEPs. Thus, the gyro-radii of almost all SEPs are smaller than the coherence scale of interplanetary magnetic turbulence and larger than the dissipation scale, meaning that the relevant part of the turbulence spectrum is the inertial range. Since most of the magnetic-field variance, or total power in the turbulent component of the magnetic field (typically one-third that of the average component) is contained at large-scale fluctuations, the field is relatively smooth on the scale of the gyro-radii of most SEPs, meaning the mean free path is typically much larger than the gyro-radii. This may not be the case near shocks where turbulence can be enhanced, most notably in the shocked plasma behind the shock.

SEP observations have been used extensively to examine charged-particle scattering in the IMF (see reviews by Palmer 1982; Bieber et al. 1994). As SEPs cross the Earth, a gradual rise to the maximum intensity is commonly observed, followed by an even more gradual decline, often lasting several days. This is suggestive of a diffusive process (e.g., Meyer et al. 1956). A common approach to determining the diffusion coefficient is to fit SEP intensity and anisotropy profiles seen by s/c to solutions of the Parker transport equation (or extensions of it that include variations in particle pitch angle). Since the average magnetic field is the well-known Parker spiral, this approach gives a reasonable estimate of $\kappa _\parallel$. A compilation of many studies of SEP (and other) events used to estimate $\kappa _\parallel$ was performed by Palmer (1982). He found that the range of values of the parallel mean free path, $\lambda _\parallel$ (${=}3\kappa _\parallel /w$), is generally between 0.08–0.3 AU for both electrons and protons covering a wide range of particle energies and rigidities.

The diffusion coefficients can also be determined from analytic theory using the well-known quasi-linear approximation (e.g., Jokipii 1966; Roelof 1968). In this approach, the equations of motion for charged particles moving in a turbulent magnetic field are solved and (typically) averaged over an ensemble of magnetic field realizations and initial particle velocities. The result gives an estimate of the pitch-angle diffusion coefficient, which can be related to the spatial diffusion coefficient (see, e.g., Jokipii 1966; Earl 1974; Luhmann 1976).

The observed (inferred) parallel mean free paths based on observations of SEPs are generally much larger than the estimates from quasi-linear theory (e.g., Palmer 1982). This topic was also reviewed recently by Giacalone (2013), who discussed other situations of unusually long parallel mean free paths, including energetic particles in the outer heliosphere.

One possible resolution of the discrepancy between theory and observations concerns the structure of the magnetic-field fluctuations. In early analyses, the so-called “slab” geometry was assumed, in which the wave vectors of the magnetic fluctuations are aligned with the average magnetic field. Such a situation leads to efficient particle scattering. It was noted by Bieber et al. (1994) that IMF fluctuations are likely to be more complex than the simple slab geometry and must have a significant component that does not readily scatter particles in pitch angle. Later, these authors concluded that the random component of the IMF is dominated by fluctuations whose wave vectors are normal to the average magnetic field, which do not efficiently scatter particles, with a smaller fraction of the total power in slab modes. The ratio of power in perpendicular modes to slab modes that best fits the observations is 80–20 % (Bieber et al. 1996).

#### Cross-field diffusion, including field-line random walk

Particle diffusion across the magnetic field is a topic of much significance to cosmic rays and SEPs. For cosmic rays entering the solar system from the outside, for example, the particles must cross the nearly transverse IMF to be observed at Earth. The topic is also important for SEPs, especially in light of recent observations of individual SEP events made by multiple s/c widely separated in solar longitude, as discussed in detail in Sect. 6.

Unfortunately, cross-field diffusion is not as well understood as parallel diffusion. There has been considerable work on this topic in the past decade or so, much of which has emphasized the importance of the dimensionality of the turbulence. Particularly, in models that contain at least one ignorable coordinate, cross-field diffusion is artificially inhibited (Jokipii et al. 1993; Jones et al. 1998), so that realistic models that lead to cross-field diffusion must be fully three dimensional (Giacalone and Jokipii 1994, 1999). Slab turbulence is a good example of a situation where there can be no cross-field diffusion. As discussed above, this turbulence model assumes that all wave vectors point in the direction of the mean magnetic field. In this situation, the components of canonical momentum of any charged particle moving in this field in the two other directions is conserved. One consequence of this is that the particles cannot move by more than one gyro-radius off the magnetic line of force from where they begin their motion.

Cross-field diffusion consists of two parts: (1) the motion of particles that move along meandering magnetic fields, which leads to significant deviations in their position relative to the *mean* magnetic field; and (2) the actual transfer of particles from one magnetic field line to the next. In most situations, the former effect dominates. This is because SEPs are of low enough energy that the field is relatively smooth on scales of the same order as the SEP gyro-radii, so that particles tend to propagate along the IMF lines more easily than across them. Motion across the local magnetic field requires some form of a scattering event. This can happen when particles scatter in pitch angle. When this happens, the particles may move normal to the local magnetic field by approximately one gyro-radius. Because the parallel mean free path is much larger than the gyro-radius, in most situations the relatively infrequent scattering events lead to inefficient cross-field transport. It is straightforward to show that the cross-field diffusion coefficient for this case—which is similar to the result obtained from hard-sphere scattering—is a small fraction of the parallel diffusion coefficient. In contrast, the cross-field diffusion coefficient resulting from charged-particle motion in meandering magnetic fields can be 2–5 % of the parallel diffusion coefficient, which is much larger. In a recent paper, Costa et al. (2013) noted that the frequent occurrence of a phenomenon known as “magnetic holes”, which are rather small pressure-balanced regions of the solar wind where the magnetic field decreases significantly from the background, can lead to a non-resonant process whereby particles are displaced normal to the average magnetic field.

As mentioned previously, it is important to make a distinction between the spatial diffusion tensor and the scattering mean free path, particularly with regard to charged-particle motion along and across the magnetic field. Generally, it is correct to relate the parallel diffusion coefficient and parallel mean free path of scattering by $\kappa _\parallel = (1/3)w\lambda _\parallel$, where $\lambda _\parallel$ is the average distance that a particle travels along the magnetic field before it is scattered, which can be thought of as the point where the particle is reflected and its motion is reversed. Upon this reflection, the particle can also move normal to the local magnetic field by a distance less than the gyro-radius, which happens because its magnetic gyro-phase becomes randomized upon scattering. When $\lambda _\parallel$ is large compared to the gyro-radii of the particles, the particles move freely along magnetic field lines with little or no motion across them. However, while not strictly incorrect, relating $\kappa _\perp$ to a perpendicular mean free path in the same way can lead to some confusion. Often we associate the mean free path with the amount of scattering. For the case of weak scattering, intuitively this means the particles move freely along the field for long distances (compared to the particle gyro-radii) between scattering events. However, this is also associated with very little motion across the local magnetic field. The confusion is that, even though the scattering is infrequent, if one chooses to define it this way, the perpendicular mean free path would be very small, which is counterintuitive.

To illustrate this, consider the special case of hard-sphere scattering in which particles move across the local magnetic field by one gyro-radius, $r_L$ (on average), each time they scatter. The ratio of the perpendicular to parallel diffusion coefficients for this case is given approximately by:

7 $$\begin{aligned} {\kappa _\perp \over \kappa \parallel } \approx \bigg ({r_L\over \lambda _\parallel }\bigg )^2. \end{aligned}$$

If we define $\lambda _\perp = 3\kappa _\perp /w$, where *w* is the particle speed, then through simple substitution and manipulation, we find that for situations in which the parallel mean free path largely exceeds the gyro-radius of the particles,

8 $$\begin{aligned} {\lambda _\perp \over r_L} \approx {r_L\over \lambda _\parallel } \ll 1. \end{aligned}$$

It makes little sense for the mean free path normal to the local magnetic field to be much less than the gyro-radius of the particles. This is counterintuitive and can lead to misconceptions about the diffusive nature of charged-particle transport across the magnetic field.

For SEPs propagating in the IMF, the dominant form of cross-field diffusion can be understood by tracking or following the meandering magnetic field lines. The origin of the field-line meandering is large-scale magnetic-field turbulence. One possible source of this is solar supergranulation (e.g., Jokipii and Parker 1968). Magnetic lines of force in the heliosphere are rooted in the plasma that originates at the Sun. Plasma motions transverse to the radial direction near the Sun, combined with the outward advection with the solar wind, lead to large-scale meandering.

Let $\mathbf{U}_{{ sg}}(\theta ,\phi )$ be the turbulent plasma motions associated with solar supergranulation (e.g., Leighton 1964) occurring at the source surface, $R_0$,^2^ where $\theta$ and $\phi$ are the polar angle and longitude, respectively. We assume a constant, radial solar wind with speed $U_w$ for any heliocentric distance $r{>}R_0$. By solving Faraday’s law, we find that the resulting magnetic field can be expressed as:

9 $$\begin{aligned} B_r(r)= & {} B_r(R_0)(R_0/r)^2, \nonumber \\ B_\theta (r,\theta ,\phi ,t)= & {} B_r(R_0)\bigg ({R_0\over r}\bigg ){U_{\theta ,sg}(\theta ,\phi ,t^\prime )\over U_w}, \nonumber \\ B_\phi (r,\theta ,\phi ,t)= & {} B_r(R_0)\bigg ({R_0\over r}\bigg ){-R_0\varOmega _\odot \sin \theta + U_{\phi ,sg}(\theta ,\phi ,t^\prime )\over U_w}, \end{aligned}$$

where $t^\prime = t - (r-R_0)/U_w$, and $\varOmega _\odot$ is the solar rotation rate. Note that the Parker spiral is recovered if $\mathbf{U}_{{ sg}}=0$.

**Fig. 60:**
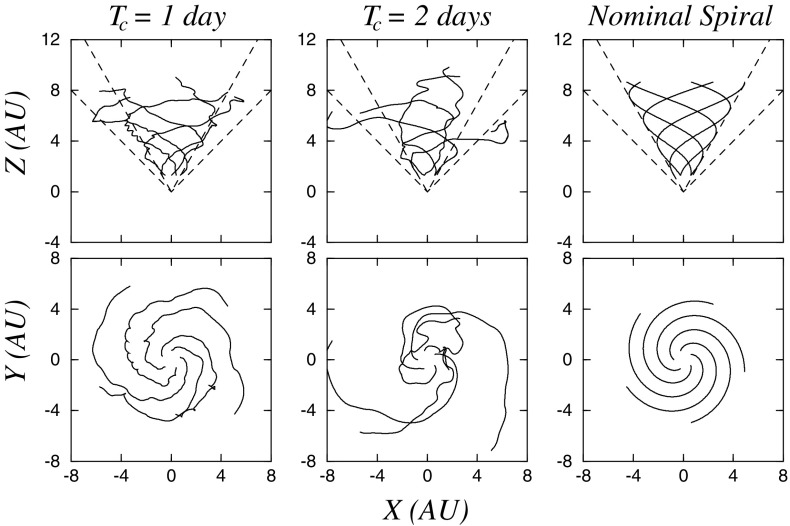
Interplanetary magnetic lines of force showing field-line meandering due to solar supergranulation, as discussed in the text. The coordinate system chosen has *Z* pointing normal to the heliographic equatorial plane. $T_c$ is the time scale associated with supergranulation that was chosen for each case. The *right column*s show the case of no transverse fluctuations at the source surface, and the result is the usual Parker spiral. Image reproduced with permission from Giacalone (2001), copyright by AGU

Figure 60 shows a plot of magnetic-field lines of force associated with Eq. (9). In the figure, $T_c$ refers to the typical lifetime associated with supergranulation (for details, see Giacalone 2001). To produce the left and middle panels, the rms speed of transverse plasma motions at the Sun was taken to be 2 km/s, which is larger than what is typically observed (0.6 km/s).

We can estimate the spreading of magnetic field lines between the Sun and 1 AU due to field-line meandering associated with supergranulation. Any given magnetic line of force is rooted at the source surface and executes a random walk across the source surface sphere. The amount of spreading of field lines at 1 AU relative to the nominal Parker spiral is equivalent to the spreading of footpoints, relative to an assumed initial location, over the time it takes the solar wind to propagate from the source surface to 1 AU. Since the motion of the footpoints is diffusive, the rms spread of field lines in the longitudinal direction at the heliographic equator, $\sqrt{\langle \varDelta \phi ^2\rangle }$, can be determined from

10 $$\begin{aligned} {R_0^2\langle \varDelta \phi ^2\rangle \over \varDelta t} = 2\kappa _{{ sg}}, \end{aligned}$$

where $\varDelta t = (1\,\mathrm{AU}-R_0)/U_w$ is the diffusion coefficient associated with supergranulation. Taking $\kappa _{{ sg}} \sim 1900\,\mathrm{km^{2}/s}$ as found by Giacalone and Jokipii (2004),^3^ we find that at 1 AU, the rms spread of field lines relative to the Parker spiral is about $3^{\circ }$, assuming a solar wind speed of 400 km/s and that $R_0$ is the solar radius $R_\odot$ (see also Wang and Sheeley 1994). This is a fairly small effect, but it can be much larger during more disturbed periods.

Borovsky (2008) has suggested that the interplanetary medium consists of many large-scale magnetic flux tubes that separate different turbulent plasmas. The walls of these flux tubes are magnetic discontinuities, and since the magnetic field is advected outwards with the solar wind, a (nearly) stationary observer would detect many such discontinuities, which is generally consistent with s/c observations. In this scenario, the magnetic field lines cannot cross the boundaries; however, the magnetic flux tubes can meander in space, and at least conceptually, the associated diffusion coefficient is similar to that associated with the random walk of field lines discussed above. Alternatively, the large number of discontinuities in the solar wind may be related to the dissipation of magnetic turbulence at small scales (e.g., Vasquez et al. 2007; Greco et al. 2008; Owens et al. 2011).

The influence of magnetic field-line random walk on the cross-field diffusion coefficient has received considerable attention in the past (e.g., Jokipii 1966; Jokipii and Parker 1968; Forman et al. 1974; Forman 1977; Bieber and Matthaeus 1997; Matthaeus et al. 1995; Gray et al. 1996; Barghouty and Jokipii 1996). Numerical simulations of cross-field diffusion in fully three-dimensional magnetic turbulence were first performed by Giacalone and Jokipii (1994, (1999), who found, rather surprisingly, that the simulation results did not agree with previous theories. In particular, the numerical simulations showed the importance of field-line random walk in enhancing the perpendicular diffusion coefficient to a value much larger than that obtained from hard-sphere scattering (mentioned above), but the numerically simulated value was considerably less than that predicted from previous theories, which included field-line random walk. The reason for this is that an important effect was missing in the theory: particles that move along meandering magnetic field lines and ultimately reverse their direction when they are scattered tend to re-trace their paths backwards along the same magnetic field lines. Quasi-linear theory (which includes field-line random walk), in contrast, essentially assumes that upon scattering, the particle will move along a new magnetic field line that is uncorrelated with the one along which the particle was previously moving. This is an oversimplification; in reality, the particles are not exactly moving along field lines due to gradient and curvature drifts or due to small deviations in the pitch angle and magnetic gyro-phase angle of the particles. The discrepancy between the numerical simulations and previous theories of perpendicular diffusion was addressed by Matthaeus et al. (2003), who suggested a new theory for particle transport. It is known as the non-linear guiding center or NLGC theory. A different assumption is used for the correlation of charged-particle trajectories relative to individual magnetic lines of force compared with that used in the quasi-linear theory. For the parameters chosen in that study, the NLGC theory agrees better with the numerical simulations presented in the Matthaeus et al. (2003) paper.

#### Beyond the diffusive approximation: focused transport

In cases where the anisotropies are large, other transport equations must be used (e.g., Roelof 1967; Ruffolo 1995; Isenberg 1997; Kóta 2000; Dröge et al. 2010; Zhang et al. 2009). The resulting transport equation is similar to the Parker equation in that it contains terms associated with advection with the plasma, energy change, drifts, and the parallel spatial diffusion coefficient being associated with pitch-angle scattering, but it also has terms that appear as a result of retaining the pitch-angle dependence of the distribution function. In large shock-associated events, the earliest arriving particles have large pitch-angle anisotropies. Thus, focused transport is particularly relevant for studying the early time behavior of large SEP events, but it is also important in studies that simultaneously solve for the generation of magnetic fluctuations due to the streaming of energetic particles accelerated by the shock, as discussed in Sect. 7.2.4. In addition, focused transport is important in situations in which charged particles undergo very weak pitch-angle scattering in regions where the magnetic field diverges strongly, e.g., near the Sun. In this case, the particles tend to be focused towards alignment with the IMF as it weakens in the expanding solar wind. This adiabatic focusing can influence the effective mean free path of the particles (cf. Kóta 2000; He and Schlickeiser 2014).

Solutions of the focused transport equation with simultaneous solutions to the magnetohydrodynamic (MHD) equations for CMEs were first performed by Kóta et al. (2005). Like the Parker equation discussed above, there are terms in the focused transport equation involving the fluid velocity and magnetic field where the results of MHD simulations of CMEs couple with particle transport. In the Kóta et al (ibid) study, the transport equation was solved along individual magnetic lines of force that were output from the MHD simulation of Manchester et al. (2005) at various times. Despite this simplification, their results revealed a reasonable qualitative agreement with the energy spectra and intensity of CME-related SEP events. This work has recently been expanded upon in much larger simulations, solving for the transport along many field lines at once (see Schwadron et al. 2010b; Kozarev et al. 2010, 2013; Dayeh et al. 2010).

#### Super-diffusion and sub-diffusion

The topic of super- and sub-diffusion has been discussed recently by a number of authors (see Klafter et al. 1987; Kóta and Jokipii 2000; Perri and Zimbardo 2008, 2009; Giacalone 2013). It is important to clarify what is meant by these terms. Particle diffusion arises from random changes in the velocity of charged particles as they encounter turbulent magnetic fields; however, depending on the level of turbulence, it takes time for an initially anisotropic distribution of particles to become isotropic. Diffusion generally applies to time scales that are large compared to the scattering time and length scales, which in turn, are large compared to the mean free path. However, for SEPs these conditions are only marginally satisfied. For example, in some SEP events, intermittent depletions or dropouts in the particle intensity are seen over very short time scales (Mazur et al. 2000; Giacalone et al. 2000a). For these events, the scattering time of the particles is comparable to the time between their observation and initial release into the heliosphere (Chollet and Giacalone 2011). Moreover, the particles move along meandering magnetic lines of force, which meander on scales much larger than the particle gyro-radii, but since the particles do not rapidly transfer between the field lines, their early time behavior is not necessarily diffusive (e.g., Laitinen et al. 2013a). It is important to understand the early time behavior of diffusion, especially as it applies to SEP transport.

For diffusion, the mean-square displacement of a particle relative to an initial reference point, $\langle \varDelta x^2\rangle$, increases *linearly* with time, *t*, i.e., $\langle \varDelta x^2\rangle \propto t$. Super-diffusion occurs when this quantity increases more rapidly. For purely ballistic motion, this would increase with $t^2$. Sub-diffusion occurs when $\langle \varDelta x^2\rangle$ either increases at a rate that is less than linear, or even decreases with time. Despite their names, neither of these are diffusive processes.

We have primarily emphasized the concepts of sub- and super-diffusion as they pertain to particle transport, but the same concepts can be applied to separation of magnetic field lines of force in turbulent magnetic fields (e.g., Ragot 2006a, b, 2011).

This topic may be relevant for understanding some SEP observations and warrants further study. For example, a common feature in SEP events associated with strong interplanetary shocks is that the energetic-particle intensity does not rise exponentially from the background to the peak intensity, which occurs at the shock (cf. Giacalone 2012). Moreover, Perri and Zimbardo (2008) showed that SEP events associated with CIRs were consistent with super-diffusive behavior.

### SEP acceleration at shocks

In this section we discuss general aspects of particle acceleration at shocks and emphasize the basic physics of shock-acceleration mechanisms, which can be applied to acceleration at CME-driven shocks—the primary focus of this review.

Before proceeding, it is important to make clear that we focus on the mechanisms of particle acceleration by shocks because there is considerable, even overwhelming, evidence that shocks are the most dominant contributors to the production of intense SEP events. In fact, it could credibly be argued that most high-energy charged particles observed in space, including galactic cosmic rays, have undergone acceleration at a shock wave at some point in their past. The largest SEP events are observed to be closely associated with strong CME-driven shocks. Galactic cosmic rays are widely believed to be accelerated by supernovae blast waves. However, while particle acceleration by shocks has been studied extensively since the early 1970s, recent observational data have presented serious challenges to our understanding of the detailed physics. While the association between enhanced intensities of energetic particles and interplanetary shocks is well documented in the literature, there is seldom a one-to-one agreement between the predictions of standard theory described in Sect. 7.2.1 and the s/c observations described earlier.

We begin with a simple derivation—the application of DSA theory at a planar shock. To achieve better agreement with s/c observations often requires extending the theory to more complicated shock geometries and including the evolution of the shock. This is especially true for SEPs accelerated by interplanetary shocks that undergo considerable variation as they move from near the Sun into the inner heliosphere.

#### The standard theory of diffusive shock acceleration, DSA

Charged particles can be accelerated by collisionless shocks provided they can move back and forth across the shock many times. They gain energy because the scattering centers are embedded in converging plasma flows across the shock (Krymsky 1977; Axford et al. 1978; Bell 1978; Blandford and Ostriker 1978). The electric field that ultimately accelerates the particles is the usual convective electric field $\mathbf{E}=-(1/c)\mathbf{U}\times \mathbf{B}$, where $\mathbf{U}$ is the plasma velocity vector and $\mathbf{B}$ is the magnetic field vector. For a collection of charged particles moving in the vicinity of a shock, the evolution of the phase-space distribution in space, momentum, and time, is given by Eq. (3), which, as previously mentioned, assumes that the pitch-angle distribution is nearly isotropic.

To obtain the so-called standard solution to DSA, one solves Eq. (3) for a shock-like discontinuity. The solution is best obtained in a frame moving with the shock, although it is important to realize that the momentum variable, *p*, in the Parker equation is defined relative to the local plasma frame. In the shock frame, consider the situation in which unshocked plasma moves opposite the unit normal to the shock front, towards the shock, with a speed $U_1$, and is then instantly decelerated to a speed $U_2$, now moving away from the shock in the downstream region. Suppose that we take the plasma motion in the shock frame to be in the *x* direction, with the shock front located at $x=0$. It is also convenient, although not strictly necessary, to assume that the spatial diffusion coefficient normal to the shock, $\kappa _{xx}$, does not depend on space.^4^ Solutions to Eq. (3) are obtained separately for the upstream and downstream regions and are matched at the shock with conservation relations. For the case of a steady one-dimensional planar shock with particle injection at the shock at a momentum $p_0$, it is readily shown that the steady-state solution to Eq. (3) is given by:

11 $$\begin{aligned} f(x,p) = Ap^{-3\zeta /(\zeta -1)}H(p-p_0) \left\{ \begin{array}{l l} \mathrm{exp}(U_1x/\kappa _{xx}(p)) &{} \quad x\le 0\\ 1 &{} \quad x > 0\\ \end{array} \right. \end{aligned}$$

where *A* is a normalization constant, $\zeta$ is the ratio of the downstream to upstream plasma density, and *H*(*p*) is the Heaviside step function. The downstream momentum spectrum depends only on the shock compression ratio.

**Fig. 61:**
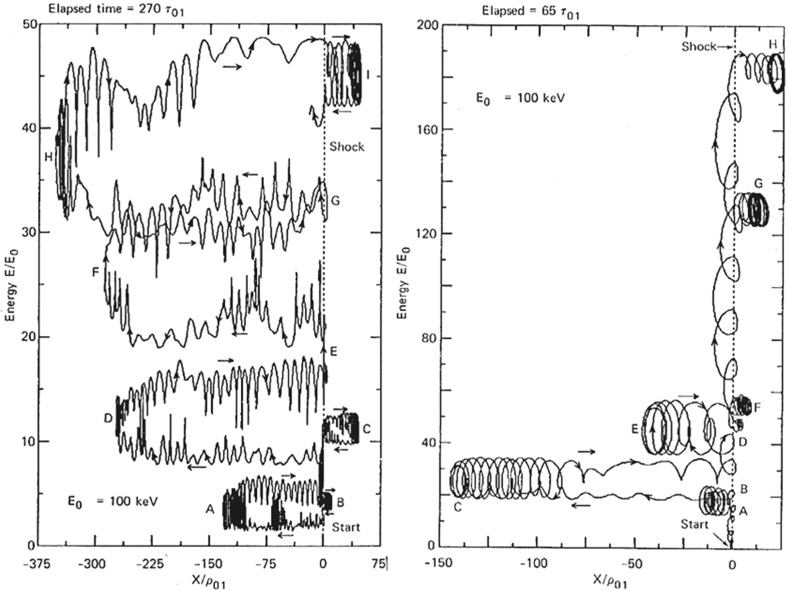
Numerical simulations of trajectories of individual charged particles moving through kinematically prescribed, turbulent electric and magnetic fields in the vicinity of two different shocks. The trajectories are displayed with kinetic energy along the vertical axis and position, relative to the position of the shock, which is fixed along the *horizontal axis*. In this frame, plasma flows from the upstream region at *left* to the downstream region at *right*. The average upstream magnetic field for the shock on the *left* makes an angle of $15^\circ$ relative to the shock normal. This is a nearly parallel shock. The shock on the *right* has an average upstream shock-normal angle of $60^\circ$, making it a quasi-perpendicular shock. The particle on the *left* was followed for 270 gyro-periods (using the average upstream magnetic field) and the one on the right for 65 gyro-periods. See the text for more details. Image reproduced with permission from Decker (1988), copyright by Kluwer, who adapted it from Decker and Vlahos (1986)

DSA contains acceleration at both parallel and perpendicular shocks, and at shocks with any shock-normal angle in between. In the case of acceleration at a shock other than purely parallel, particles can gain energy by drifting along the shock front due to the gradient in magnetic field across the shock, which is in the same direction as the convective electric field. This drift acceleration is contained in DSA. In the special case where there is no pitch-angle scattering, this form of acceleration is often called shock-drift acceleration (e.g., Armstrong et al. 1985). First-order Fermi acceleration is another term that is often used when describing particle acceleration at shocks. However, it is important to correct a common misconception: first-order Fermi acceleration is not the same as DSA. First-order Fermi acceleration (e.g., Fermi 1949) occurs when particles gain energy by scattering between converging magnetic clouds. This is analogous to particle acceleration at shocks, but it lacks drift acceleration that occurs directly at the shock. In fact, defined in this way, a strong case could be made that first-order Fermi acceleration likely occurs nowhere, since real shocks move through large-scale magnetic-field turbulence, which results in a non-zero angle between the local magnetic field and unit shock normal. Thus, there are always places along the shock where some drift acceleration occurs at the shock even when it is parallel on average (cf. Giacalone 2005a).

Figure 61 shows simulations of the trajectories of two charged particles encountering two different shocks, one that is nearly parallel (left plot), and one that is quasi-perpendicular (right plot). The particle on the left was followed more than four times longer than the particle on the right. The energy is measured relative to the shock frame of reference, which leads to increases and decreases in the energy, depending on whether it is moving along or opposite the motional electric field during its gyro-motion. Note that for the case of a parallel shock, this particle gains most of its shock-frame energy by scattering upstream of the shock. In fact, this particle loses shock-frame energy when it scatters downstream of the shock because the scattering centers, which are embedded in the plasma flow, are retreating from the shock. Even in this case, episodes of energy gain directly at the shock due to drift acceleration arise because the local magnetic field is oblique to the shock front, as discussed above. For the particle on the right, most of the energy gain comes by drifting along the shock. Although it is dangerous to draw far-reaching conclusions from inspection of individual particle trajectories, it is clear that acceleration at oblique shocks is faster than that at quasi-parallel shocks. This is discussed further below.

#### Relating predictions of the standard theory to SEP observations

In Sect. 3.3, we discussed studies that compared DSA theory predictions to SEP observations, and here we extend that discussion to relate some of the theory’s most basic predictions to general aspects of the observations. Perhaps the simplest of these is that the intensity of energetic particles increases in the upstream region and reaches a maximum at the shock, after which it remains constant in the downstream region. The particles are accelerated directly at the shock, which is the source, and then advected downstream of it, leading to the constant intensity. Thus, if DSA theory is correct, we expect that particles that are locally accelerated by a propagating shock will peak at the time of the shock passage. Indeed, energetic particles are commonly seen to be associated with heliospheric shocks. In the 1960s, the idea was put forth that high-energy charged-particle events seen at Earth were often associated with traveling interplanetary disturbances (Bryant et al. 1962; Rao et al. 1967). Spacecraft observations later revealed that these SEPs were often seen peaking at the same time s/c were overtaken by interplanetary shock waves (e.g., Armstrong et al. 1970; Sarris and Allen 1974; Gosling et al. 1981; Scholer et al. 1983; Decker 1983). High-energy charged particles are also commonly seen to be associated with CIRs (Barnes and Simpson 1976; McDonald et al. 1976). The classic exponential rise to the shock passage, followed by an essentially constant intensity downstream, is occasionally seen (Kennel et al. 1986; Giacalone 2012). For large SEP events, it is common to see an intensity enhancement at the time the shock crosses 1 AU (cf. the review by Reames 1999).

However, the DSA theory prediction of an exponential rise of the particle intensity to the shock, followed by a constant intensity downstream of it, is not commonly observed, even for low-energy SEPs. It is almost never seen for the highest-energy SEPs. On the one hand, as discussed in Sect. 3.1, part of the discrepancy can be attributed to the strength of the shock—low-energy SEP intensities look much more like the DSA prediction for the strongest interplanetary shocks. On the other hand, the standard theory of DSA discussed above assumes a steady-state distribution, and this is not the case for SEPs accelerated at IP shocks. The time dependence is critical and is discussed further below. We note that, in propagating shocks the energetic particle intensity must decay behind the shock, partly because of adiabatic cooling. Moreover, the IMF strength decreases with distance from the Sun, affecting the diffusion coefficient, which affects the rate at which particles are accelerated (see Sect. 7.2.4). Closer to the Sun, the acceleration rate is much higher than it is at 1 AU. Thus, high-energy particles can be accelerated close to the Sun by the shock; however, when the shock reaches 1 AU, it is no longer accelerating particles (efficiently) at these energies. This would lead to the particle intensity at very high energies peaking well before the shock, which is commonly observed.

Another important prediction of DSA is that of a power-law dependence of the differential intensity spectrum on energy. In many cases, SEPs exhibit power-law energy or momentum spectra with an index that varies over a small range from some low, initial energy to a higher energy where there is a transition to a roll-over or to a different, usually steeper, power law (due to losses or finite acceleration time). More than 40 years ago, Syrovatsky (1960) explicitly discussed this and pointed out that it was an important clue about the mechanism or mechanisms responsible for particle acceleration. This issue of a power-law spectrum remained unresolved until the discovery of DSA. DSA gives a power-law index that depends only on shock compression ratio [see Eq. (11)]. In practice, since the relevant shocks in the inner heliosphere are usually at least moderately strong, the distribution function is expected to be $f(p)\,p^{-4\,\mathrm{to}\,-6}$. In the situation of finite time or spatial scales, the spectrum is the asymptotic power law at lower momenta with a rollover above some higher energy. This suggests that DSA is involved in the acceleration of many, if not most, of the SEPs observed in space (also see Channok et al. 2005).

More recently, Fisk and Gloeckler (2006, (2008) reported that suprathermal particles are not only power laws in velocity (or momentum), but that the index of the power law is most often $-5$, and deviates within a range of 0.2–0.3 from this value (Decker et al. 2008). This could be the result of shock acceleration, but there is as yet no widely accepted explanation of this preferred power-law index.

It is also important to note that the calculated power-law spectra are hard enough that the particles themselves can modify shocks. Although this is usually not seen, it may occur at very fast CME shocks, such as the type recently reported by Russell et al. (2013), and possibly at the solar wind termination shock as reported by Florinski et al. (2009).

#### The rate of acceleration and maximum energy

Of particular importance to understanding the intensity and energy spectra of SEPs is the rate of acceleration, which is directly related to the maximum energy achievable by the mechanism of shock acceleration. We note that SEPs are commonly seen many minutes to hours after the occurrence of the associated solar flare or CME, and since it also takes time for the particles to reach 1 AU because of scattering in the IMF, the acceleration must occur quickly.

Analytic time-dependent solutions to Eq. (3) can be obtained for the simple case of a planar shock (e.g., Drury 1991). The result is that the energy spectra of accelerated particles behind the shock is a power law from some initial injection momentum $p_0$ up to a momentum $p_c$ where the spectrum rolls over to a steeper spectrum, which, for the simplest case, is exponential. The time it takes a one-dimensional planar shock with a unit normal pointing in the $-x$ direction to accelerate particles from $p_0$ to $p_c$ is given by (e.g., Drury 1983; Forman and Drury 1983):

12 $$\begin{aligned} \tau _A = {3\over U_1-U_2}\int _{p_0}^p {dp^\prime \over p^\prime }\bigg ({\kappa _{xx,1}(p^\prime )\over U_{x,1}}+{\kappa _{xx,2}(p^\prime )\over U_{x,2}}\bigg ), \end{aligned}$$

where the subscripts 1 and 2 refer to the upstream and downstream regions of the shock, and $U_x$ is the flow speed normal to the shock, in the shock frame of reference. $\kappa _{xx}$ is the particle diffusion coefficient defined in the direction normal to the shock and can be related to the components along and across the magnetic field, $\kappa _\parallel$ and $\kappa _\perp$, respectively, and the angle between the unit normal to the shock and average magnetic field vector, $\theta _{Bn}$, by

13 $$\begin{aligned} \kappa _{xx} = \kappa _\parallel \cos ^2\theta _{Bn}+\kappa _\perp \sin ^2\theta _{Bn}. \end{aligned}$$

By inspection of Eq. (12), we see that the time it takes to accelerate particles is directly related to the diffusion coefficient—the smaller the diffusion coefficient, the smaller the acceleration time. Given a certain amount of time over which to accelerate particles, a shorter acceleration time means that the maximum energy attainable is larger. Thus, high energies are achieved when the diffusion coefficient is small. This, of course, makes sense intuitively because the acceleration is most rapid when particles are confined near the shock.

It is also interesting to note that acceleration time depends on diffusion coefficients both upstream and downstream of the shock. The plasma behind shocks is well known to be turbulent, which generally means that the downstream diffusion coefficient is much smaller than the one upstream. Thus, the acceleration time is dominated by the upstream diffusion coefficient.

**Fig. 62:**
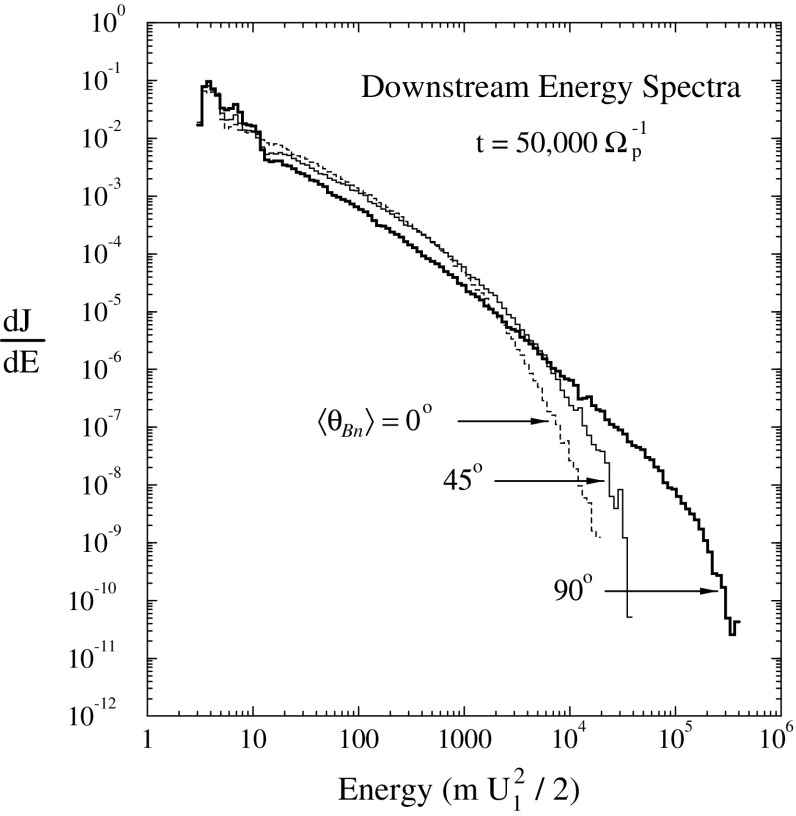
Distributions of charged particles averaged over the downstream region of three shocks that differ only in the the angle between the average upstream magnetic field and the unit shock normal direction. The distributions were obtained from test-particle orbit integrations of particles moving through kinematically prescribed electric and magnetic fields associated with strong collisionless shocks. Image reproduced with permission from Giacalone (2005a), copyright by AAS

We note from Eq. (13) that perpendicular shocks are more rapid accelerators of particles than parallel shocks because, typically, $\kappa _\perp \ll \kappa _\parallel$. Figure 62 shows the results from test-particle orbit integrations that illustrate this point. The simulation results (for details see Giacalone 2005a) shown in this figure are distributions downstream of three strong shocks with the same parameters, except that the average shock-normal angle is varied. All distributions are time dependent and obtained at the same time. As the figure shows, when the shock is perpendicular, on average, the distribution extends to the highest energies. The case shown is for relatively strong turbulence with a variance that is equal to the mean field squared. The disparity between parallel and perpendicular shocks is even larger if the turbulence is a bit weaker, as discussed in Giacalone (2005a).

**Fig. 63:**
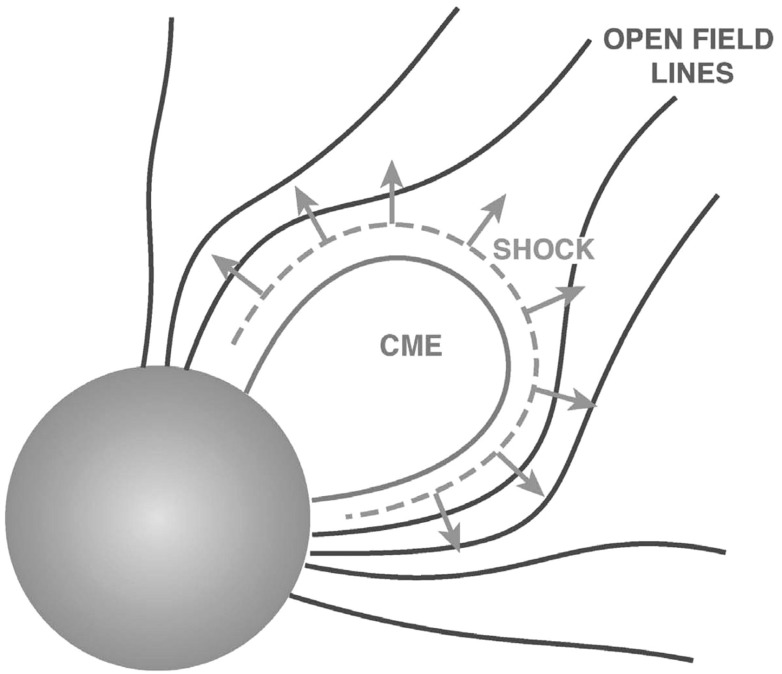
Cartoon illustration of a CME-driven shock expanding in the solar corona (courtesy of Allan Tylka)

This is likely important for understanding acceleration of SEPs by CME-driven shocks. As a CME-driven shock expands through the corona, there are places on the shock where the propagation direction is normal to the magnetic field. In these locations, the acceleration is most rapid and the highest energies are attained. Figure 63 illustrates a shock expanding in the solar corona and shows perpendicular locations at the flanks where efficient particle acceleration to high energies may occur.

The concept of the maximum energy must be considered carefully. In time-dependent diffusive shock acceleration for a planar shock, the spectrum of accelerated particles has a power-law dependence on momentum, *p*, up to a critical momentum, $p_c$. The value of $p_c$ increases with time according to the rate of acceleration discussed above. For $p>p_c$ the spectrum deviates from the power law and becomes exponential. In the context of DSA theory, we use two terms interchangeably, maximum energy and break-point energy, which refer to the point where the spectrum deviates from a pure power law at $p=p_c$. The true maximum energy cannot be easily determined because the process is diffusive, and there is a finite number of particles with momenta well beyond $p_c$. For heliospheric shocks, and likely others as well, the intensity at momenta well beyond $p_c$ can also be influenced by the existence of background high-energy particles, possibly produced by other phenomena not associated with the shock. For example, fast CMEs are almost always associated with solar flares, which can also accelerate particles to high energies but are not (likely) associated with the CME-driven shock seen at 1 AU.

It has long been thought that acceleration of particles to the highest energies must involve Bohm diffusion^5^. For instance, the maximum energy of GCRs accelerated at a supernova blast wave is obtained by setting the acceleration time given in Eq. (12) to the lifetime of the blast wave, assuming a parallel shock and Bohm diffusion ($\kappa _\parallel = \kappa _\mathrm{Bohm} = (1/3)wr_G$, where $r_G$ is the gyro-radii of the particles), and solving for the maximum energy (e.g., Lagage and Cesarsky 1983). Similarly, models of SEP acceleration at evolving CME shocks also assume Bohm diffusion (e.g., Zank et al. 2000). It is important to note that Bohm diffusion is *not* the smallest diffusion coefficient possible, since $\kappa _\perp$ can be smaller than $\kappa _\mathrm{Bohm}$ in many situations. This can be shown by determining the ratio of the acceleration time to that for Bohm diffusion. If we write $\kappa _\perp = \epsilon \kappa _\parallel$, where $\epsilon \ll 1$ and is independent of energy (e.g., Giacalone and Jokipii 1999), we find

14 $$\begin{aligned} {\tau _{A,\perp }\over \tau _{A,\mathrm{Bohm}}} = {\kappa _\perp \over (1/3)wr_G} = {\kappa _\perp \over \kappa _A} = \epsilon \eta , \end{aligned}$$

where $\eta = \lambda _\parallel /r_G$, which can be shown to decrease with energy according to quasi-linear theory. For reasonable parameters, ratio $\tau _{A,\perp }/\tau _{A,\mathrm{Bohm}}$ is smaller than unity for the highest-energy particles. Thus, perpendicular shocks are faster accelerators of particles than what was previously thought to be the most extreme case of Bohm diffusion. Moreover, Bohm diffusion results from particles moving in extremely turbulent magnetic fields that are seldom (if ever) observed in space, at least in-situ by s/c. Turbulence is known to be enhanced downstream of shocks, but as noted above, the acceleration time depends mostly on the diffusion coefficient upstream of the shock, and enhanced waves are only occasionally observed upstream of interplanetary shocks. Thus, perpendicular shocks likely play a major role in the acceleration of SEPs at CME-driven shocks.

**Fig. 64:**
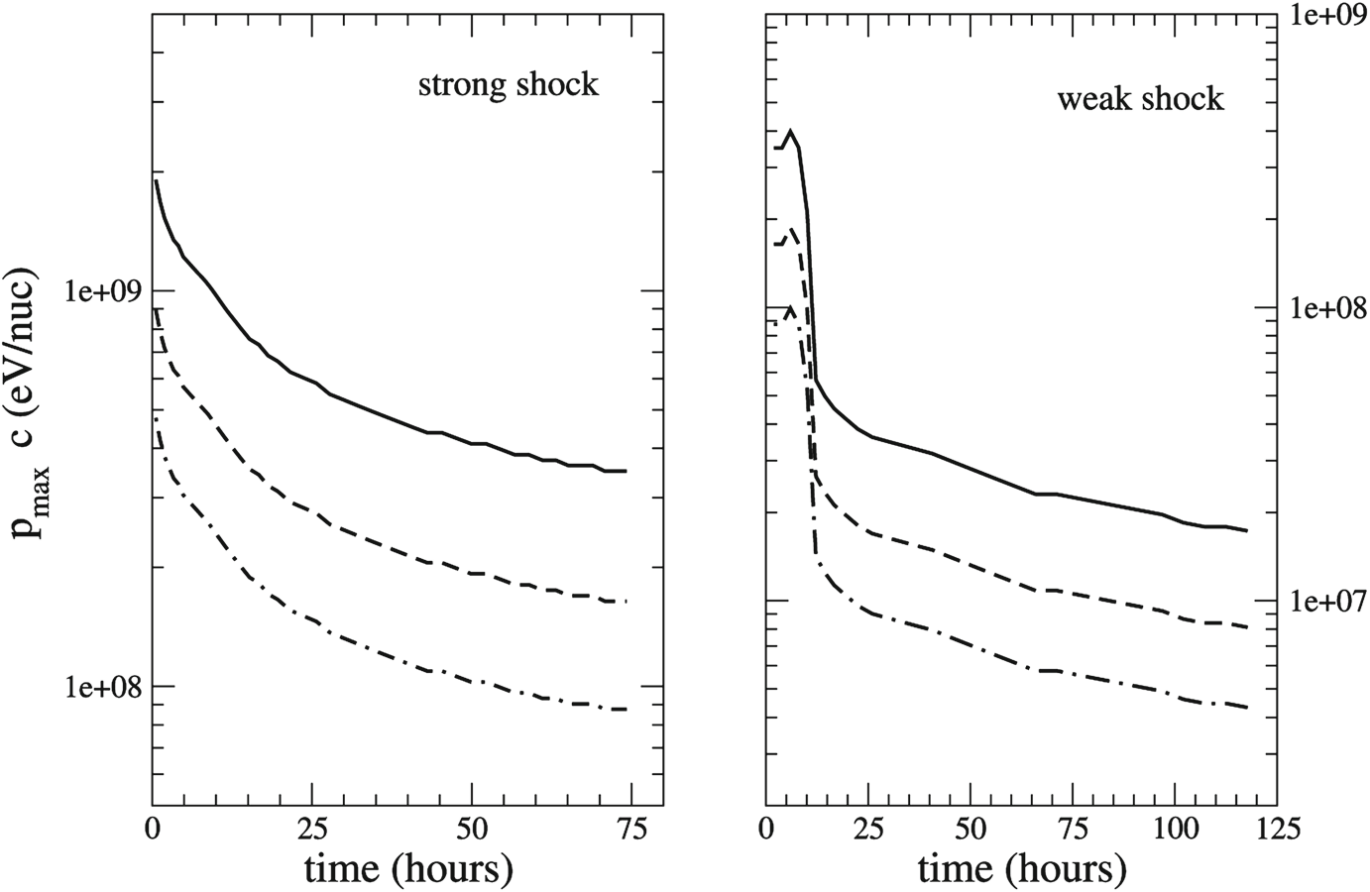
The maximum momentum from diffusive shock acceleration at two different CME-driven shocks, at the shock location, as a function of time as the shock moves outwards in the heliosphere. The curves are for protons (*solid*), CNO ions (*dashed*), and Fe ions (*dot-dashed*). Image reproduced with permission from Li et al. (2005), copyright by AGU

It is complicated to determine the maximum energy attainable from CME-driven shocks because of the various time scales involved. While the general characteristics of the DSA mechanisms are still applicable, there is no reason to expect that predictions of the simple steady-state theory should apply. For example, the shock itself changes with time since it weakens as it propagates outward. The heliospheric magnetic field also changes with distance from the Sun, causing the geometry of the shock and the relevant particle diffusion coefficients to change with time. Factors such as the age and speed of the shock and the strength of the local magnetic field largely determine the maximum energy attainable. Figure 64 shows the maximum energy at a CME-driven shock from a study by Li et al. (2005). It is interesting to note that the maximum energy decreases with heliocentric distance, despite the fact that the shock is moving outward and therefore older. The key reason for this decrease is that the stronger magnetic fields near the Sun lead to smaller spatial diffusion coefficients, which give an increased acceleration rate, and hence a higher maximum energy.

We have not addressed the question of how particles are “injected” into the mechanism of DSA. Some have argued that perpendicular shocks, while rapid accelerators, do not inject and accelerate low-energy thermal particles efficiently. In fact, in most situations applicable to the acceleration of SEPs at CME shocks—or most situations in heliospheric and astrophysical shocks in general—injection of lower-energy particles at perpendicular shocks may not be an issue. This is discussed in detail in Sect. 7.2.6.

#### Magnetic fluctuations excited by shock-accelerated ions

Shocks accelerate particles rapidly when the spatial diffusion coefficient is small; one way this situation arises is when the acceleration occurs at perpendicular shocks. For quasi-parallel shocks, however, the only way the acceleration can be rapid is when the magnetic turbulence, upstream and downstream of the shock, is sufficiently high. In fact, it has been known for some time that the energetic particles themselves can excite magnetic fluctuations that also act to scatter them (e.g., Bell 1978; Lee 1983, 2005; Lee et al. 2012).

We note that the spatial diffusion coefficient appearing in the Parker equation is related to the magnetic-field turbulence power spectrum. For instance, the parallel diffusion coefficient is related to the pitch-angle diffusion coefficient, $D_{\mu \mu }$, (e.g., Earl 1974; Luhmann 1976) by

15 $$\begin{aligned} \kappa _\parallel (v)={w^2\over 4}\int _0^1{(1-\mu ^2)^2d\mu \over D_{\mu \mu }}, \end{aligned}$$

where $D_{\mu \mu }$ can be obtained from quasi-linear theory (e.g., Jokipii 1966) and is related to the magnetic-field power spectrum by

16 $$\begin{aligned} D_{\mu \mu }={\pi \over 4}\varOmega _0(1-\mu ^2){k_{{ res}}P(k_{ res})\over B_0^2}, \end{aligned}$$

where $k_{ res}=|\varOmega _0/w\mu |$ is the resonant wave number, $B_0$ is the average magnetic field strength, and $\varOmega _0$ is the gyro-frequency. Note that in this case of a parallel shock, resonant scattering is the primary contributor to the spatial diffusion coefficient. Thus, resonant scattering also determines the critical momentum, $p_c$. The dependence of scattering on the resonant wave mode gives rise to an explicit dependence of $p_c$ on the gyro-frequency of the particles, which will determine the rigidity dependence of the spectral rollover (e.g., Tylka et al. 1999; Ng et al. 1999).

The magnetic-field power spectrum, *P*, in Eq. (16) may be composed of: (1) a part that is associated with the pre-existing magnetic-field turbulence generated in the solar corona or solar wind independent of the shock, and (2) a part that is due to an instability arising from the streaming of energetic particles relative to the background magnetic field upstream of the shock. Particles stream relative to the shock because of the gradient in their intensity from the background to a peak at the shock. As they scatter, they can impart some of their energy to the waves, leading to wave growth (e.g., Lee 1983, 2005; Gordon et al. 1999).

**Fig. 65:**
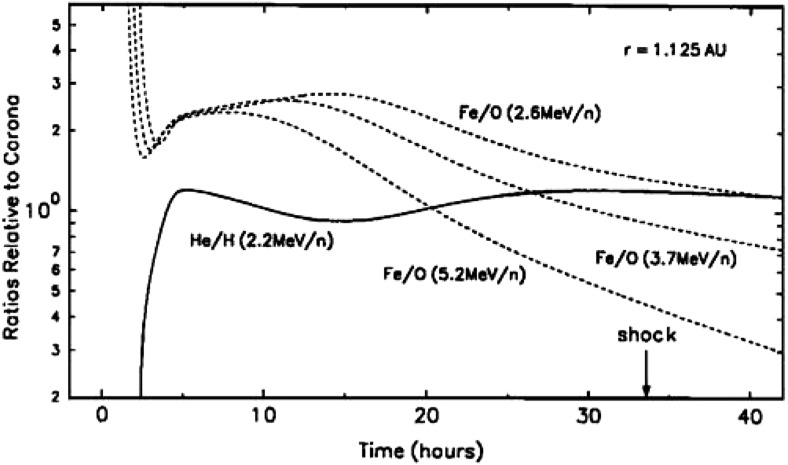
Elemental abundance ratios of various heavy ion nuclei associated with a large, gradual SEP event as a function of time seen by an observer located 1.125 AU from the source of the event. Time is measured relative to the start of the event. The shock arrival time is indicated by a *vertical arrow*. Image reproduced with permission from Ng et al. (1999), copyright by AGU

We note that variations in the elemental composition of large gradual SEP events may be related to the presence of self-excited waves (this was also discussed in Sects. 2.8, 2.9). For instance, Fig. 65 shows results of a model that includes SEP transport of various heavy ion nuclei in the presence of proton-generated magnetic fluctuations. Plotted are various elemental abundance ratios at a given energy-per-nucleon, as indicated, as a function of time. In this case, an observer is located at 1.125 AU from the Sun, and the shock arrives at the observer just over 30 h from the beginning of the simulated event, as indicated.

Consider the Fe/O ratio at 2.5 MeV/nucleon, for instance. The initially very large value is caused by the fact that Fe, having a larger gyro-radius than O at the same energy-per-nucleon, presumably has a larger mean free path away from the shock, in the ambient turbulent magnetic field. Thus, Fe ions of a given energy-per-nucleon reach the distant observer before O ions of the same energy-per-nucleon. Thus, the ratio of Fe/O is high. As the shock moves outward and closer to the observer, the O intensity increases, and the Fe/O ratio decreases. Then, a few hours after the start of the event, this ratio begins to increase gradually for several hours. Ng et al. (1999) interpreted this as resulting from the presence of proton-amplified magnetic fluctuations that presumably become important a few hours after the onset of the event (also see review by Reames 1999). The physical picture is that O ions have a rigidity that is closer to that of the protons, which excite the magnetic fluctuations, than the Fe ions. Hence, O is trapped more efficiently near the shock than Fe, leading to fewer of the O ions reaching the observer. Thus, the Fe/O ratio will increase once the proton-amplified waves become stronger. This lasts for several hours before the Fe/O ratio finally begins to decrease again. The decrease happens only after the self-excited waves are no longer intense.

It is important to note that multiple time scales can affect the temporal behavior of the heavy ion abundance ratios. These include: (1) the time scale of the growth of the waves, (2) the acceleration time scale of the ions, (3) the shock propagation time scale, and (4) the time scale associated with changes in the large-scale magnetic-field orientation. The physical picture described above is certainly plausible, but many issues remain open (see Sect. 8). One is that self-consistent kinetic simulations, such as the well-known hybrid simulation, reveal that the upstream waves do not agree well with theoretical predictions (e.g., Giacalone 2004). Moreover, the theory usually only considers a direct exchange in energy between the energetic particles and the magnetic fluctuations at the appropriate resonant wave mode (discussed above) but ignores non-linear steepening of the waves. Such hybrid simulations reveal that the upstream fluctuations contain a considerable, even dominant contribution from large-amplitude magnetic structures that result from steepening of the smaller-amplitude waves.

More recently, the equations that couple the growth of magnetic fluctuations and particle acceleration at shocks have been included in many numerical models of SEP acceleration at CME-driven shocks (e.g., Zank et al. 2000; Vainio 2003; Ng et al. 2003; Vainio and Laitinen 2007; Ng and Reames 2008; Li et al. 2009; Verkhoglyadova et al. 2009). One key result is that acceleration to the highest energies occurs—even up to and beyond a GeV—closer to the Sun, where the background magnetic field is large, and the intensity of self-excited waves is greatest. As a CME-driven shock moves outward towards 1 AU, the background field becomes much weaker, the spatial diffusion coefficient of the particles becomes much larger, the wave growth is not as rapid, and the waves are less intense. Thus, the waves that were strongest near the Sun get advected downstream of the shock. There should, however, remain some enhancement in wave power observed at IP shocks at 1 AU in the portion of the magnetic power spectrum with wave modes that are resonant with energetic particles seen enhanced at 1 AU (typically an MeV or less). There are some examples where these self-excited waves are seen upstream of shocks at 1 AU (e.g., see Fig. 39 of this review, and also Zank et al. 2006; Bamert et al. 2004; Tsurutani et al. 1983), but such observations are rare. Since such self-excited waves are rarely seen upstream of IP shocks near Earth, one science objective for upcoming inner heliospheric missions—Solar Probe Plus and Solar Orbiter—is to search for these types of waves as they get closer to the Sun, and understand how they affect SEP acceleration and transport (see Sect. 8).

#### The role of magnetic-field geometry

Because energetic charged particles move along the magnetic field more easily than normal to it, the geometry and temporal evolution of magnetic field lines play a crucial role in determining the resulting energetic particle spatial distribution, energy spectra, anisotropy, and composition. The longer the time that magnetic lines of force are connected to a region where efficient particle acceleration occurs, such as at a shock, the larger the particle intensity is expected to be. As soon as the field line is no longer connected to the shock, the particles on that field line are no longer accelerated. Moreover, curved shocks, and/or curved magnetic lines of force, can also lead to multiple points of connection of the field lines and shock (e.g., McComas and Schwadron 2006; Guo et al. 2010; Kóta 2010). Thus, the overall geometry of the magnetic field and shock plays an important role in determining the intensity at any given point along the shock.

**Fig. 66:**
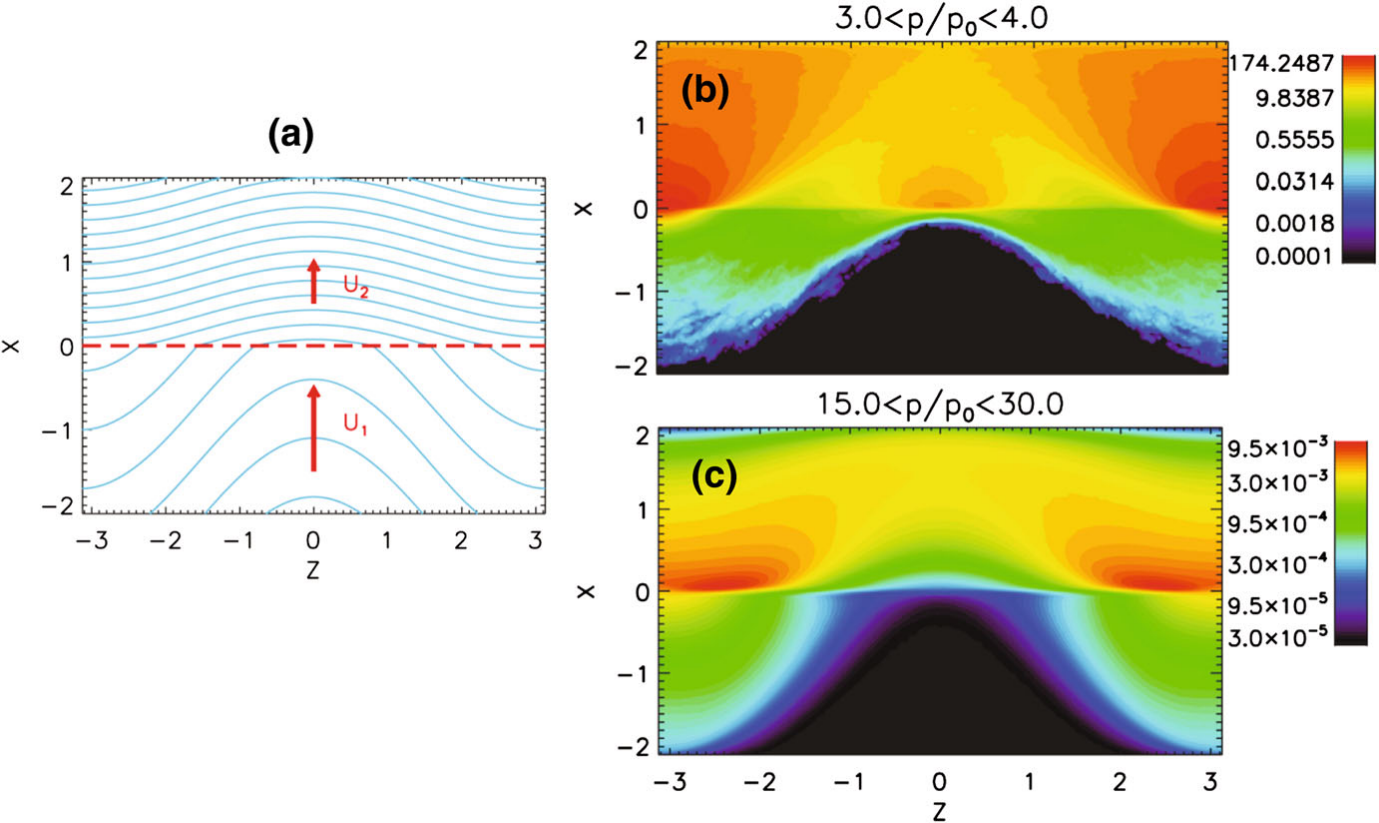
Solutions to the Parker equation for particles accelerated at a shock moving through a spatially dependent magnetic field whose lines of force connect to the shock in two places. **a** Geometry of the magnetic field lines in the upstream and downstream regions. *Color-coded* representations of the distribution function of particles of **b** low energy (3–5 times the injection momentum), and **c** high-energy particles (15–30 times the injection momentum), where *red* is the most intense and *black* is the least intense. Image reproduced with permission from Guo et al. (2010), copyright by AAS

Figure 66, from the study of Guo et al. (2010), shows a good example of this (see also Kóta 2010). These authors solved the Parker equation for energetic test particles that are accelerated at a planar shock moving through a spatially dependent magnetic field whose lines of force connect to the shock in two places, with the connection points either moving towards or away from each other. The geometry is shown in Fig. 66a. Figure 66b, c shows color-coded contours of the distribution function of accelerated particles throughout the two-dimensional domain. Figure 66b is the distribution of particles of a lower momentum than that of Fig. 66c. This is a steady-state calculation. The intensity of the highest-energy particles in Fig. 66c is largest towards the sides of the simulation domain, with a much lower intensity in the center. This is presumably related to the interpretation given in the preceding paragraph. The shock itself may be curved and produce similar effects. The acceleration of ACRs at a blunt-shaped termination shock is another example where such shock curvature effects may be important (e.g., McComas and Schwadron 2006; Kóta and Jokipii 2008; Schwadron et al. 2008; Senanayake and Florinski 2013).

Curved shocks and or quasi-planar shocks moving through large-scale, spatially varying magnetic fields can also have other important effects. For one, they affect the observed pitch-angle anisotropies. For example, as an IP shock approaches a s/c, it could enter a region where the magnetic field configuration connects the observer to the shock at two places, thus allowing the s/c to observe bi-directional pitch angle anisotropies (e.g., Decker 1990). In addition, variations in the geometry of interplanetary shocks as they evolve from the solar corona outward to 1 AU have also been used to explain unusual SEP abundance variations (e.g., Tylka et al. 2005; Tylka and Lee 2006; Sandroos and Vainio 2007).

#### The “injection problem”

Much recent progress has been made on the issue of how low-energy ions, including thermal particles, are accelerated by shocks, which is a process that cannot be described by Eq. (3). In particular, in numerical simulations of quasi-parallel shocks, a fraction of the incident plasma population is extracted out of the thermal pool by reflection at the shock layer to become seed particles for further acceleration (Quest 1988; Scholer 1990; Giacalone et al. 1992). This has also been shown to be true for quasi-perpendicular shocks, but requires sufficient pre-existing large-scale turbulence (Giacalone 2005a, b).

DSA applies when the Parker equation can be used, which is when the anisotropy of the particles is small. The anisotropy results from the particle intensity gradient upstream of the shock. The vector anisotropy is defined as:

17 $$\begin{aligned} \delta _i = {3S_i\over wf}, \end{aligned}$$

where *w* is the particle speed measured in the local plasma frame, and $S_i$ is the vector diffusive streaming flux. It is typically assumed that the particle speed is much larger than the flow speed. However, here we are concerned with situations in which this may not be the case, but for which the Parker equation may still be valid. Thus, we consider the diffusive streaming of particles as measured in the local plasma frame given by:

18 $$\begin{aligned} S_i = -\kappa _{i,j}{\partial f\over \partial x_j}, \end{aligned}$$

where $\kappa _{i,j}$ is the spatial diffusion tensor. If we were concerned with the streaming flux in the shock frame, we would also need to include another term associated with the so-called Compton–Getting effect.

For a planar shock moving in the $-x$ direction, the distribution function *f* is given by Eq. (11). Using the expression for *f* upstream of the shock and substituting it into Eqs. (17) and (18), and by writing the diffusion tensor in terms of components along and across the magnetic field and the average shock normal angle (Eq. 13), the magnitude of the anisotropy is given by:

19 $$\begin{aligned} \vert \delta _i\vert = {3U_1\over w}\bigg [1 + { (\kappa _A/\kappa _\parallel )^2\sin ^2\theta _{Bn}+ (1-\kappa _\perp /\kappa _\parallel )^2\sin ^2\theta _{Bn}\cos ^2\theta _{Bn} \over \big ((\kappa _\perp /\kappa _\parallel )\sin ^2\theta _{Bn}+\cos ^2\theta _{Bn}\big )^2}\bigg ]^{1/2}\ll 1, \end{aligned}$$

where $\kappa _A = wr_g/3$ is the antisymmetric component of the diffusion tensor. This equation first appeared in Giacalone and Jokipii (1999) and is in a slightly different form in Zank et al. (2006), who included the Compton–Getting term mentioned above.

It is instructive to examine the anisotropy in three extreme cases: weak scattering, a purely parallel shock, and a purely perpendicular shock (for other cases, see Giacalone and Jokipii 2005; Zank et al. 2006). For the case of very weak scattering for which $\kappa _\parallel \gg \kappa _\perp ,\kappa _A$, we obtain

20 $$\begin{aligned} \vert \delta _i\vert _{\mathrm{weak~scattering}} = {3U_1\sec \theta _{Bn}\over w}\ll 1. \end{aligned}$$

Since $U_1\sec \theta _{Bn}$ is the speed at which the intersection point of any given magnetic field line moves along the shock, this result can be understood intuitively. For the anisotropy to be small, particles must have sufficient speed to stay ahead of the shock. The resulting injection energy for this case (the *w* for which the anisotropy is small) is a strong function of $\theta _{Bn}$.

For the case of a parallel shock, Eq. (19) reduces to

21 $$\begin{aligned} \vert \delta _i\vert _{\theta _{Bn}\rightarrow 0} = {3U_1\over w}\ll 1. \end{aligned}$$

And for a perpendicular shock, it reduces to

22 $$\begin{aligned} \vert \delta _i\vert _{\theta _{Bn}\rightarrow {\pi /2}} = {3U_1\over w}\bigg [1 + \bigg ({\kappa _A\over \kappa _\perp }\bigg )^2\bigg ]^{1/2}\ll 1. \end{aligned}$$

The expression for the parallel shock is relatively easy to understand, but interpreting the expression for the perpendicular shock case is not as clear. In situations of rather weak turbulence (but not so weak that the weak-scattering limit applies), one might expect that $\kappa _A\gg \kappa _\perp$, for which simple manipulation of Eq. (22) leads to the result that the Parker equation is applicable when $\kappa _\perp U_1/r_G\gg 1$. This is the same condition derived by Jokipii (1992). However, for stronger turbulence in which the variance is comparable to the mean, $\kappa _\perp$ can exceed $\gg \kappa _A$. When this happens, a perpendicular shock has the *same* injection velocity as a parallel shock. Physically, this corresponds to the case where field-line meandering is sufficiently large that the motion of low-energy particles normal to the shock is enhanced enough that they can participate in DSA. As noted above, hybrid simulations of perpendicular shocks that include large-scale magnetic turbulence have revealed that even thermal plasma can be efficiently accelerated at a perpendicular shock, which provides justification of the analytic result we have derived.

**Fig. 67:**
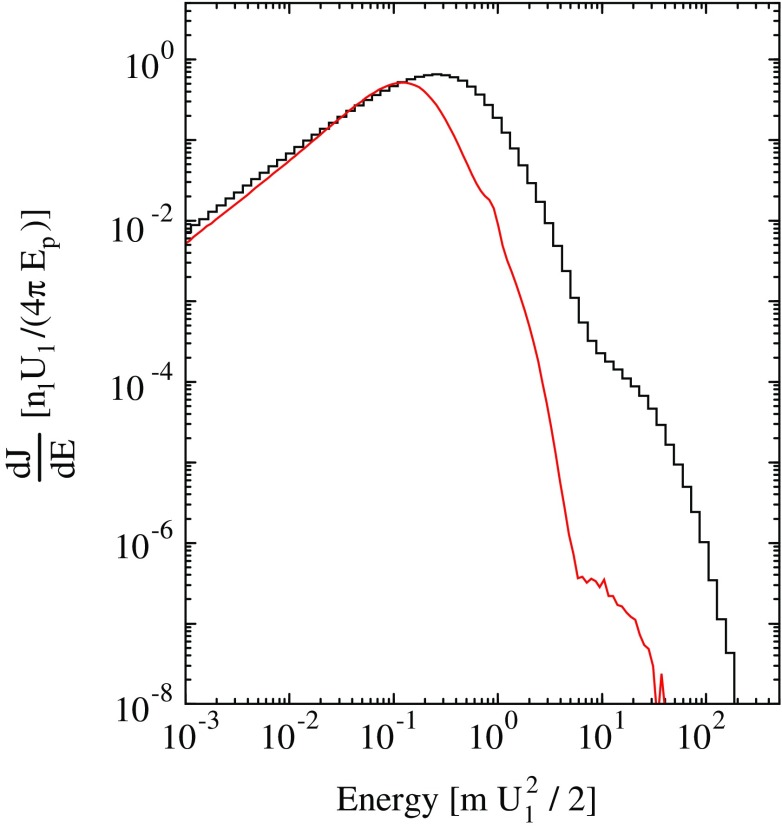
The distribution of thermal protons in the shock-heated plasma downstream of two collisionless shocks with similar Mach number and shock-normal angle, and initial thermal proton temperature. For the distribution shown in *red*, the upstream plasma consisted only of the thermal protons and electrons. The distribution in *black* includes thermal protons and electrons, as well as suprathermal pickup ions with density ${\sim }30\,\%$ that of the thermal protons. These distributions are discussed in greater detail in Giacalone (2005a) and Giacalone and Decker (2010)

As stated earlier, many studies have shown that the source of SEPs seems to be pre-existing suprathermal ions and not thermal solar wind. This suggests a possible link between the upstream distribution and that of the accelerated particles. Figure 67 shows an example of the importance of the nature of the upstream distribution on the acceleration efficiency of low-energy protons. Each case shows the incidences of the distributions of initially thermal solar wind protons on two different shocks, but with similar parameters. The distributions were obtained from simulations presented in two studies published separately (Giacalone 2005a; Giacalone and Decker 2010). The shocks in each case were of relatively high Mach number, and both were perpendicular shocks. The main difference between the two cases is that for the black-histogram distribution, the plasma incident on the shock contained only thermal protons and electrons and no pre-existing suprathermal particles, whereas for the red-histogram distribution, in addition to thermal protons and electrons, the incident plasma contained a significant number of freshly ionized pickup ions. In the latter case, the addition of the pickup ions, which are a suprathermal population, contained enough of the incident energy flux that the shock did not require thermal protons to be accelerated to provide the necessary shock dissipation (cf. Leroy et al. 1981; Winske 1985), i.e., specularly reflected ions were not necessary for this case. The result is that when pickup ions were present in sufficient numbers, the acceleration efficiency of thermal solar wind was significantly reduced, compared to the case where there were no pickup ions present. This likely has consequences for the observed compositional variations seen in SEPs.

In a recent study, Neergaard-Parker and Zank (2012) estimated the injection energy for a number of observed quasi-parallel interplanetary shocks. Assuming that the diffusive approximation was valid, they first theoretically determined the distribution of energetic particles behind the shock based on an assumed distribution upstream of the shock. The upstream distribution was based on the observed plasma temperature and density. They then compared the observed energy spectra at higher energies with their analytic forms of the downstream distribution to determine the injection energy. They found values ranging from 1 to 3 keV, which is consistent with the theoretical expectations discussed above. They recently extended their analysis to observed quasi-perpendicular shocks (Neergaard-Parker et al. 2014). Other recent studies have emphasized the importance of self-excited waves (as discussed in Sect. 7.2.4) on the acceleration of low-energy and/or thermal ions at shocks (e.g., Battarbee et al. 2013), as well as certain aspects of the shock micro-physics such as the cross-shock electric field (e.g., Zuo et al. 2013).

#### The effect of pre-existing large-scale turbulence

It is well known that the solar wind and IMF are turbulent. The level of turbulence varies, but often the variance in the fluctuations of quantities such as the magnetic field strength, plasma density, and plasma velocity are observed to be comparable to the means in those quantities. Thus, the shocks that accelerate particles cannot be considered to exist in isolation, and the effects of turbulence must be considered. It is now apparent that shocks are significantly affected by this pre-existing turbulence. For example, by combining observations from multiple s/c, Neugebauer and Giacalone (2005) showed that the fronts of interplanetary shocks are not planar, but instead are warped or rippled with a typical local radius of curvature comparable to the coherence scale of IMF turbulence (Jokipii and Coleman 1968; Matthaeus et al. 1986). It is likely that turbulence caused this rippling in the shock fronts. This can be clearly seen in numerical simulations of shocks moving through pre-existing turbulence (e.g., Giacalone 2005b; Giacalone and Neugebauer 2008; Giacalone and Jokipii 2007). The shock-surface rippling and pre-existing large-scale turbulence likely have significant affects on the observed time-intensity profiles of energetic particles (Neugebauer et al. 2006) as well as on their energy distributions (Giacalone and Neugebauer 2008). Indeed, Giacalone and Neugebauer (2008) used MHD simulations of shocks moving through large-scale turbulence, combined with solutions to the Parker equation describing the acceleration of particles at the shock, and showed that the resulting energy spectrum was essentially independent of location along the shock, despite the fact that the jump in plasma density across the shock varied along the shock face because of turbulence effects. One consequence of this is that observations of a single IP shock made by several s/c are likely to see similar energetic-particle energy spectra. The Giacalone and Neugebauer study showed three IP shocks that support this notion.

We also note that, in general, the power spectrum of magnetic fluctuations associated with pre-existing turbulence is directly related to the diffusion coefficients, as discussed in above in Sect. 7.1. Moreover, the diffusion coefficients are directly related to both the acceleration rate and injection efficiency, as discussed in Sect. 7.2. Therefore, the level of turbulence has a significant effect on both the rate and effiency of the acceleration. As one example, as a shjock moves through a plasma, it generates magnetic fluctuations which can lead to enhanced turbulence in the dostream plasma, behind the shock. If a second shock moves through this enhanced turbulence, it will likely accelerate particles faster, and more efficiently.

#### Acceleration at multiple shocks

During periods of high solar activity, shock waves from the Sun can occur at a frequency ranging from a few hours to a few days. During much of solar maximum, the Sun emits as many as 5 or more CMEs per day (Gopalswamy et al. 2007; Olmedo et al. 2008; Robbrecht and Berghmans 2004), a significantly higher number and frequency than during solar minimum. Simple models that treat CME-driven shocks in isolation are thus unlikely to be applicable to the vastly more turbulent conditions of solar maximum. Moreover, some of the largest SEP events observed, including one during the late October–early November 2003 period (Mewaldt et al. 2005a), as well as a large event seen in the summer of 2012 by STEREO A (Russell et al. 2013), were preceded by at least one other shock in somewhat close proximity to the one associated with the main SEP event.

DSA theory has been applied to particle acceleration by multiple shocks by Melrose and Pope (1993) and Schneider (1993). Their analyses considered that the shocks are identical and that the accelerated particles from one shock are injected into the next shock in the sequence. The result is that the spectrum downstream of each successive shock is flatter than the preceding one, but does not become harder than $f\propto p^{-3}$ (also see Sect. 2.5).

The effect of multiple shocks on particle acceleration could be better understood by using more realistic parameters for the shocks. Observations of very large events, as mentioned above, suggest quite strongly that acceleration at multiple shocks is important. In addition to the fact that energetic particles may encounter more than one shock, other consequences may also be just as important for producing high particle intensities at a shock that moves through plasma heated by a preceding shock. For example, since shocks are known to produce plasma and magnetic-field turbulence, the second shock will likely encounter higher magnetic-field turbulence variance, which efficiently traps particles near the second shock. This will lead to a more rapid acceleration at the shock and a higher maximum energy. Moreover, the hot plasma will contain more seed particles available for acceleration. A twin-CME model for producing GLE events, which includes these effects, was recently proposed by Li et al. (2012) and discussed in Sect. 2.5.

#### Particle acceleration at gradual plasma compressions

Any compression of the plasma, including a shock, may lead to particle acceleration. In the purely diffusive limit, for which the Parker equation (3) applies, the acceleration depends on the divergence of the plasma velocity. Thus, shock-like particle energization processes can occur even if the plasma gradually compresses over a scale larger than the thickness of a shock. In fact, the resulting distribution of charged particles undergoing compression acceleration is the same as that for shock acceleration in the limit

23 $$\begin{aligned} \varDelta _c {<} {1\over 2}{\varDelta U\over U} r, \end{aligned}$$

where $\varDelta _c$ is the thickness of the compressed plasma, *U* is the flow speed relative to the Sun, $\varDelta U$ is the change in *U* across the compression, and *r* is the heliocentric distance to the compression.

Non-shock, gradual plasma compressions are known to exist in the heliosphere, most notably in association with CIRs. Giacalone et al. (2002) explicitly discussed the acceleration of particles at plasma compressions associated with CIRs, finding that particles can be accelerated locally at the compressions. While CIRs are usually bound by forward and reverse shocks at large distances from the Sun, the shocks are not often observed at 1 AU, suggesting that the plasma compresses more gradually there. Some recent observations of energetic particles associated with CIR compression regions are consistent with local acceleration of charged particles in the vicinity of the gradual plasma compression, rather than with remote acceleration at the forward/reverse shocks, followed by subsequent transport to the observer as suggested by Fisk and Lee (1980).

For the case of a gradual compression associated with a CIR at 1 AU, using typical values of $U=800\,\mathrm{km/s}$ (fast solar wind), $\varDelta U=400\,\mathrm{km/s}$, and $r=1\,\mathrm{AU}$, we find from Eq. (23) that $\varDelta _c < 0.25\,\mathrm{AU}$ leads to the acceleration of particles in a manner consistent with diffusive shock acceleration. If we assume that the compression itself moves at, e.g., 400 km/s, the rise from slow to fast wind would occur over a timescale of ${\sim }24$ h. For the CIRs studied by Mason et al. (2008), the observed time scale was on the order of a few hours. Thus, such compressions are capable of accelerating particles locally (also see Bučík et al. 2009; Ebert et al. 2012a, b).

Gradual plasma compressions can also exist near the Sun, or in the inner heliosphere, in association with CMEs. These compressions may also accelerate SEPs, but as of the time of writing this review we are unaware of any published studies documenting this effect. One possible observational consequence of acceleration of SEPs at a gradual plasma compression near the Sun would be the lack of an associated type II radio burst emission with the SEP event. According to Table 1 of Cane et al. (2010), there are several SEP events with no associated type II bursts which might be interpreted as evidence of acceleration at a plasma compression and not at a shock (Gopalswamy et al. 2008).

In addition to acceleration at isolated plasma compressions, such as those associated with CIRs, acceleration may also occur at random, stochastic plasma compressions (e.g., Bykov 1982; Ptuskin 1988; Jokipii et al. 2003; Giacalone et al. 2005). This has been suggested as an acceleration mechanism occurring in the solar wind (Fisk and Gloeckler 2006, 2008) and heliosheath (Fisk and Gloeckler 2009) to explain the observed suprathermal tails of energetic particles in these regions. In the context of the Parker equation, we note that in the case of stochastic plasma compressions, the particles essentially undergo a random walk in the logarithm of the magnitude of the particle momentum, leading to a high-energy tail in momentum. The mechanism is likely too slow, and therefore not important, to overcome adiabatic cooling in the solar wind.

#### Acceleration of electrons

Energetic electrons are also commonly seen at shocks in the heliosphere. Because of their small gyro-radii, it is unclear what traps them near the shock, since there is little power in the turbulent IMF at these scales due to dissipation of turbulence below the proton gyro-radius (e.g., Leamon et al. 1999). However, electrons move very fast, and even mildly suprathermal electrons have speeds that are faster than most shocks. Jokipii and Giacalone (2007) explicitly pointed out that fast-moving electrons closely follow meandering magnetic field lines that intersect the shock, or any other plasma compression, many times. Guo and Giacalone (2010) extended this analysis to include the microphysics of the shock. Moreover, ripples in the shock front may also lead to similar effects (e.g., Burgess 2006).

Electrons are known to be efficiently accelerated in solar flares (Lin et al. 2003). While the mechanisms of particle acceleration in flares are not well understood, magnetic reconnection is certainly involved, and even shocks may play a critical role. Guo and Giacalone (2012) performed simulations of electron acceleration at a solar-flare termination shock that is thought to exist below (the sunward side of) the x-line of a magnetic reconnection event where the outflowing plasma exhaust encounters strong magnetic fields closer to the Sun. They found that the acceleration of electrons was extremely efficient. Moreover, Li et al. (2013) showed that the physical thickness of the shock—being of the order of the thermal ion gyro-radii, which is much larger than that of suprathermal electrons—can affect the resulting shock-accelerated energy spectrum and may even explain the observed spectral features.

### Other possible SEP acceleration mechanisms

This review has focused mostly on large gradual SEP events, and it is widely known that these events are most commonly associated with CME-driven shock waves. Therefore, our theoretical review has focused mostly on the mechanism of particle acceleration at shocks. However, other mechanisms have been proposed to explain SEPs, most of which relate to the acceleration of ions and electrons in solar flares. Of course, shock acceleration may also be a dominant mechanism in flares, since shocks are thought to exist in the solar-flare environment. It is instructive to mention some other mechanisms here, though there are no observations that convincingly show direct evidence for particle acceleration in interplanetary space associated with any of these mechanisms. In fact, although magnetic reconnection has been observed directly in the solar wind (Gosling et al. 2005b), no reports of associated energetic particles exist (Gosling et al. 2005a).

#### Swann’s mechanism

One of the earliest acceleration mechanisms to be discussed was that proposed by Swann (1933), who showed that a magnetic field that increased with time, like that associated with a sunspot when it appears on the Sun, has an induced electric field that can accelerate particles. Swann’s model for electron acceleration in sunspots assumed that the magnetic field extended radially outward from the solar photosphere and the induced electric field was normal, so that the electrons gained energy as they gyrated around the magnetic field. For a 1000 gauss magnetic field, typical of fields in large sunspots, electrons could reach energies of about 10 GeV in as little as 1 s. However, this model did not explain what would happen when the field eventually decreased, nor did it address the issue of how the electrons escaped. In both cases, the field experienced by a particle gets weaker, and the particle would lose much of the energy that it had previously gained.

This mechanism has recently been applied to acceleration of SEPs in solar flares through their interaction with collapsing magnetic traps (cf. the review by Grady et al. 2012). This is similar to the physics of particle acceleration within collapsing magnetic islands associated with magnetic reconnection, discussed below.

#### Second-order Fermi acceleration

Fermi (1949) later proposed a similar mechanism to that of Swann’s, but included the effect of particle scattering by magnetic fluctuations. This mechanism is currently known as second-order Fermi acceleration. As a charged particle is scattered by a fluctuating magnetic field, it either gains or loses energy, depending on whether the scattering center is moving towards or away from the particle. In the frame of reference moving with the magnetic fluctuations, there is no associated electric field, and the particle’s kinetic energy is conserved during the scattering process. But, in the inertial frame, the particle’s kinetic energy will change. Fermi noted that there are statistically more head-on collisions, leading to a gain in the particle’s kinetic energy, compared to those in which the particle must catch up to the fluctuations and lose energy upon scattering. Thus, there is a net increase in the kinetic energy of the particles.

The process is diffusive in the magnitude of particle momentum. Thus, second-order Fermi acceleration can be included in the transport equation discussed in Sect. 7.1 by adding a term representing momentum diffusion, and introducing the momentum diffusion coefficient $D_{{ pp}}$ given by:

24 $$\begin{aligned} {1\over p^2}{\partial \over \partial p} \bigg (p^2D_{{ pp}} {\partial f\over \partial p}\bigg ) - {f\over \tau _\mathrm{loss}} = 0, \end{aligned}$$

where $\tau _\mathrm{loss}$ is the timescale associated with the loss of particles from the system.

The usual assumption in second-order Fermi acceleration is that $D_{{ pp}} \approx (v_A/w)^2 p^2/\tau _\mathrm{scat}$, where $v_A$ is the Alfvén speed, *w* is the particle speed, and $\tau _\mathrm{scat}$ is the scattering time.

We consider a relatively simple situation to illustrate a key point concerning the importance of this mechanism in the acceleration of SEPs. Although this is an unlikely situation, suppose that both $\tau _\mathrm{scat}$ and $\tau _\mathrm{loss}$ are independent of momentum. In this simple case, Eq. (24) yields a solution having a power-law dependence of *f* on *p*. The resulting power-law index can be shown to depend on $\tau _\mathrm{loss}, \tau _\mathrm{scat}$, and $v_A$. These can all vary considerably depending on the application, leading to a significant variation in the power-law spectral index from one event to the next. As noted above, power-law distributions are indeed common in SEP events, but the spectral index typically varies by less than a factor of 2. For this reason, Syrovatsky (1960) noted that this mechanism cannot explain the observed power-law distributions of energetic particles. In addition, solutions to the momentum diffusion equation above do not necessarily yield a power law when more reasonable forms for the scattering and loss time scales are included.

Second-order Fermi acceleration can be made more general by considering types of magnetic fluctuations other than those considered by Fermi. For instance, resonant stochastic acceleration considers particle acceleration by various types of plasma waves and relies on a specific resonance condition associated with the frequency and wavelength of a specific type of wave and the momentum of the particle. $D_{{ pp}}$ in Eq. (24) is determined through the resonance condition appropriate to a particular plasma wave type. In the case of solar flares, a common approach is to assume that large magnetic loops in the lower solar corona contain hot, turbulent plasma consisting of various plasma waves. The transport equation is solved by assuming the appropriate form of $D_{{ pp}}$ (and, possibly other terms involving the pitch-angle cosine $\mu$, such as $D_{p\mu }$, etc., if the distribution also depends on $\mu$, e.g., Petrosian and Liu 2004; Miller 2000; Emslie et al. 2004). This mechanism is thought to be responsible for the large enhancement of $^{3}$He relative to $^{4}$He seen in energetic particles associated with impulsive solar flares (Fisk 1978).

#### Acceleration in magnetic islands associated with reconnection

Another statistical acceleration mechanism associated with magnetic reconnection in solar flares involves the interaction of charged particles with magnetic “islands” that exist between layers of current that separate oppositely directed magnetic fields (e.g., Drake et al. 2006; Oka et al. 2010). Numerical simulations of an individual current layer of width, *L*, reveal that multiple islands of closed magnetic field structures are created during the onset of magnetic reconnection. These islands can grow to *L*. Islands can also contract, and this leads to particle acceleration as particles move along the closed magnetic fields whose lengths decrease as the islands contract, leading to an increase in the ion’s parallel kinetic energy. The energy gained by moving within a single magnetic island is relatively small; thus, to get significant energy gain, the particles must undergo acceleration within many contracting islands. Power-law energy spectra may result if the acceleration is also balanced by losses from the system.

#### Acceleration in random plasma compressions

Another statistical mechanism is described by Fisk and Gloeckler (2006, (2008) to explain the commonly observed $f \sim p^{-5}$ spectrum discussed in Sect. 5. This mechanism involves the acceleration of particles by random compressions of the plasma. Note that in the Parker transport equation, energy change results from a divergence in the plasma velocity vector. Thus, any compression of the plasma, and not just a shock, can accelerate particles. In fact, Giacalone et al. (2002) showed that, when the fast solar wind overtakes the slow SW at a CIR, a shock does not form instantly, and yet particles can be accelerated by the gradual compression of the plasma between the two different types of solar wind. This can be generalized to random compressions, as discussed by Giacalone et al. (2005) and is also similar to the mechanism discussed by Fisk and Gloeckler (2006, (2008). However, quantitative analyses involving these mechanisms only explain the shape of the resulting energy spectrum, and do not make any specific predictions about the time-intensity variations of the energetic particles. Since shocks are also capable of producing the observed power-law spectra, and energetic particles are known to be enhanced at shock fronts and not necessarily in the turbulence behind them, most attention has been focused on the mechanisms of shock acceleration. Furthermore, since these other mechanisms are unlikely to play a major role in SEP acceleration, they have received little attention.

#### Acceleration in direct electric fields associated with reconnection

In addition to statistical mechanisms, direct electric fields associated with magnetic reconnection may also accelerate particles. In the standard model of magnetic reconnection, an electric field exists near the magnetic null, or X point, which points normal to the external magnetic field. This electric field may accelerate particles (e.g., Litvinenko 1996) in such a way as to produce a power law, depending on how close to the X point the particles get. The highest energy particles are the ones that are closest to the X point. Litvinenko (2006) found that SEPs with energies of a few hundred MeV can be achieved by acceleration within a three-dimensional fan-model for magnetic reconnection, using typical parameters near the Sun.

This mechanism is conceptually straightforward in a steady-state, two-dimensional solar-flare model. However, recent work on magnetic reconnection has emphasized its complexity. Many models reveal the existence of magnetic islands and small-scale plasma turbulence. Three-dimensional magnetic reconnection is likely quite different from the two-dimensional picture in the standard model. It is not clear whether direct electric fields play a major role in the acceleration of energetic particles in solar flares. And, as noted, there is no evidence for particle acceleration at magnetic reconnection events seen in the solar wind (Gosling et al. 2005a).

## Key open questions and future missions

**Fig. 68:**
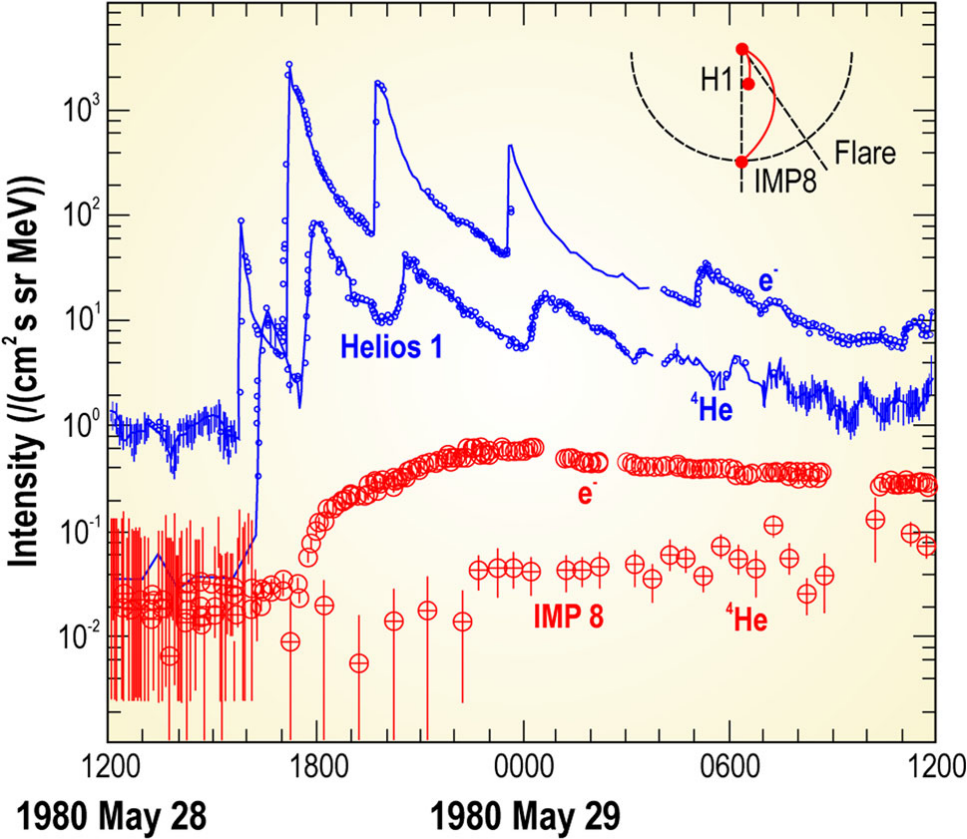
Electron (e) and He ($\alpha$) time profiles from Helios 1 (0.3 AU) and IMP 8 (1 AU) during five SEP events in 1980. Magnetic connections to the flare site are indicated at *upper right*. Helios 1 observed five separate injections, while IMP 8 observed only one. Future missions, SPP and SolO, will enable us to separate the effects of transport by making key near-Sun measurements where SEP acceleration takes place. Image adapted from Wibberenz and Cane (2006)

While recent modeling and observations have substantially improved our understanding of the origin, acceleration, and transport mechanisms that govern the behavior of SEPs observed at 1 AU, many puzzles remain unsolved. This occurs primarily because testing, discriminating, and refining SEP acceleration models using 1 AU data alone is difficult due to mixing from different sources and smearing during transport (see Sect. 2.8). Helios data demonstrated the clear advantages of venturing closer to the Sun to investigate SEP processes near their origin (see Fig. 68). Figure 68 provides a powerful example of the need for inner heliospheric observations. Here the Helios 1 s/c located at ${\sim }0.4\,\mathrm{AU}$ detected at least five separate impulsive-like injections of electrons and He ions, while IMP 8 at 1 AU observed dramatically different time-intensity profiles. This indicates that scattering and diffusion during transport between 0.4 AU to Earth orbit smeared out the time profiles at 1 AU, thereby registering a single particle event rather than ${>}5$, as seen at Helios 1.

Important unanswered questions regarding SEP events observed at 1 AU are:

1. What causes the large event-to-event variations in key SEP properties such as peak intensity, maximum energy, temporal and spectral profiles, event-integrated fluences, etc.? In particular, we need to quantify the relative contributions of seed populations, co-temporal flares, jets, and CME shocks to large gradual SEP events. We also need to determine whether, and under what conditions, the associated flares contribute high-energy particles directly or in the form of suprathermal seed populations to large SEP events. Finally, we need to identify the origins of the seed populations and determine how their inherent temporal and spatial variations affect SEP properties.
2. Do self-excited, proton-generated Alfvén waves exist and how do they affect SEP properties such as spectral breaks and roll over energies? Specifically, we need to understand the relative roles of ambient turbulence/waves and self-generated waves in the trapping and escape of SEPs during acceleration and transport.
3. How does scattering during transport modify SEP spectra, abundances, and temporal profiles? Here we need to quantify the roles of diffusion and scattering during near-Sun acceleration and propagation through the inner heliosphere and link these physical effects to SEP properties observed in the distant heliosphere, e.g., at 1 AU.
4. How do coronal and interplanetary magnetic field configurations affect the energization and escape of SEPs from their acceleration regions? To address this question, we need to understand the structure and dynamics of coronal and interplanetary magnetic field topologies and the formation and evolution of CMEs and their shocks, and how these are related to specific SEP properties.
5. Where are the highest energy SEP protons accelerated during GLEs? We need to quantify the relative roles of flare-related magnetic reconnection-driven acceleration processes, and CME-shock associated DSA and other mechanisms. Specifically, we need to determine the magnetic field geometry and the conditions under which flare-accelerated SEPs can escape out into the interplanetary medium and understand the complex coronal environments in which CMEs can form shocks in the lower solar atmosphere below ${\sim }4\,R_{S}$.

To address Question 1, recent numerical simulations and analytical models have incorporated particle acceleration at dynamically evolving IP shocks. While these are highly promising (e.g., Li et al. 2003; Lee 2005), much work still needs to be done to achieve closure between the theory and observations. One source of uncertainty is the identity of the seed particles and their related effects. These include understanding the exact manner in which particles are injected into the acceleration processes and how their inherent variability affects the SEP properties. During the 1980s, it was presumed that since CMEs propagate through the solar wind, their shocks would accelerate the ambient solar wind material. In addition to the solar wind, prior SEP events were also proposed as possible candidates for supplying the source populations for IP shocks (e.g., Forman and Webb 1985; Tsurutani and Lin 1985; Tan et al. 1989). However, instruments flown during that era lacked the sensitivity to measure small compositional differences, and so the question of the origin of the seed particles for CME shocks remained unanswered. Instruments on ACE, Wind, and Ulysses showed that, in addition to the SW peak at low energies, particle fluences measured at 1 AU also exhibited a continuous presence of an ST tail extending out to cosmic ray energies (e.g., Gloeckler 2003). In addition, contemporary instruments also provided compelling evidence that seed particles for CME shocks originate from the ST tail rather than from the more abundant solar wind peak (e.g., Desai et al. 2006a). However, the numerous controversies (see Sect. 5) surrounding the origin of the ST tails themselves need to be resolved. Venturing closer into the inner heliosphere and making simultaneous measurements of the ST tail densities and the accelerated SEPs as functions of radial distance and solar activity cycle will provide definitive clues about the origin of the ST tail material and help quantify the role of their variability in SEP events.

**Fig. 69:**
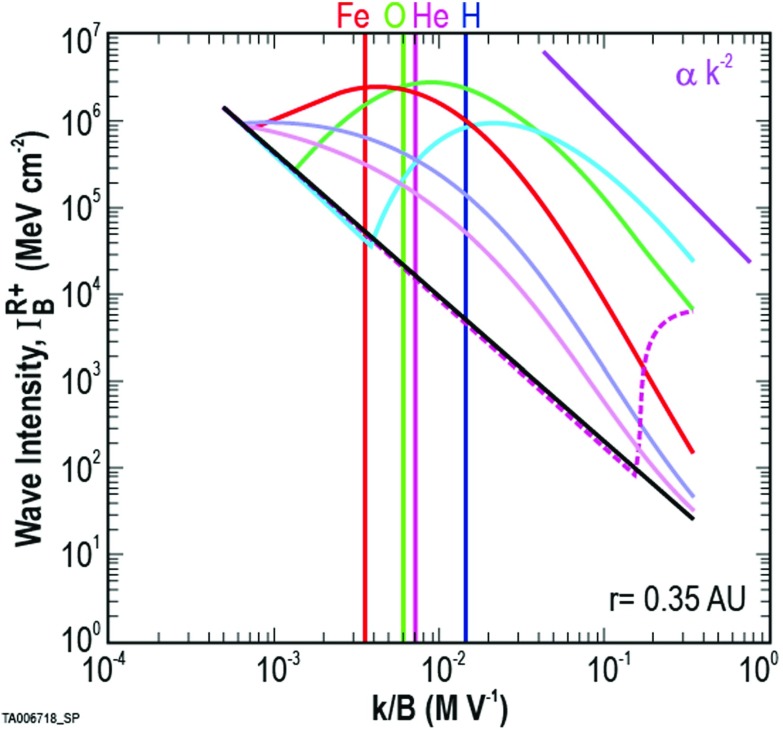
Simulations of proton-amplified Alfvén-wave spectra during a large SEP event. Accelerated ions resonate at lower wave numbers (from *right* to *left*), and once they pass the peak, the acceleration efficiency and slope of the spectrum decrease. Solar Probe Plus and Solar Orbiter will enable measurements of such wave spectra inside 0.5 AU in multiple CME shock-associated events and determine how they affect SEP properties such as temporal evolution of heavy ion composition and spectral breaks over a broad energy interval. Image reproduced with permission from Ng et al. (2003), copyright by AAS

Regarding Question 2 above, we remark that, although DSA is thought to be the primary mechanism that accelerates particles at CME-driven shocks, several unresolved issues continue to hamper the development of theoretical models of large gradual SEP events. These include: (1) existence and effects of proton-amplified Alfvén waves near quasi-parallel shocks, (2) conditions that affect the acceleration efficiencies of CME shocks, and (3) roles played by shock geometry on the injection thresholds. According to DSA models, proton-amplified Alfvén waves trap ions near quasi-parallel shocks, greatly increasing acceleration efficiency (Lee 1983), and perhaps occasionally resulting in Q/M-dependent breaks in the heavy ion spectra between ${\sim }1$ and ${\sim }30\,\mathrm{MeV}$ (Mewaldt et al. 2005c; Cohen et al. 2005b; Li et al. 2009). While such waves are difficult to observe near Earth and are seldom seen (e.g., Bamert et al. 2004), significantly greater wave intensities are expected near the Sun (see Fig. 69), where sudden decreases in wave intensities around wave numbers corresponding to the spectral break-point rigidities, e.g., ${\sim }0.005\,\mathrm{MV}^{-1}$ in Fig. 69, would provide decisive observational confirmation of the roles of diffusion and wave amplification in SEP events.

**Table 3:** Open questions, possible causes, and contributions from future inner heliospheric missions

Open questions	Possibilities/effects	SPP and SolO contributions
What causes event-to-event variations in SEPs?	Seed populations, Twin CMEs, shock properties, flare contributions	Identify variations in seed populations and determine how they affect CME shock acceleration efficiency and SEPs
Do self-excited proton-generated Alfvén waves exist, and how do they affect SEPs?	Q/M-dependence of low-energy spectral flattening; radial and energy dependence of peak intensities	Study properties of events with self-excited waves, and correlate with associated increases in streaming-limited peak intensities and the possible lack of spectral flattening
How does scattering during transport modify SEPs?	Rigidity-dependent scattering and associated variations or direct flare contributions	Identify and quantify the contributions of flares to large SEP events as transport-related time variations diminish
How do coronal and interplanetary magnetic field configurations affect SEPs?	SEP acceleration and transport in the presence of CMEs, shocks, and other large-scale structures in the low corona and interplanetary medium	Determine CME shock formation and propagation, properties of evolving CMEs, shocks, and other large-scale coronal and IP structures and their relationships with ambient turbulence spectra and SEP properties
Where are the highest energy SEP protons accelerated?	CME shocks in the low corona or flares	Use onset-time analyses to reduce uncertainties and identify source regions in individual SEP events

Similar breakthroughs can also be expected for Questions 3–5. Clearly then, the next decade promises to revolutionize our understanding of SEP acceleration and transport by exploration of the solar corona and inner heliosphere with state-of-the-art sensors on board upcoming missions such as Solar Probe Plus, SPP (http://solarprobe.jhuapl.edu; McComas et al. 2014) and Solar Orbiter, SolO (Müller et al. 2013). While Helios provided SEP observations from unique vantage points, the particle instruments only measured protons, alphas, and electrons in limited energy ranges, as shown in Fig. 68. The inclusion of heavy ions and the extension to much higher and lower energy ranges means that the two s/c probing the inner heliosphere will acquire a more comprehensive SEP dataset than did the two Helios s/c. In particular, both SolO and SPP will make in-situ measurements of the solar wind plasma, fields, waves, and suprathermal and energetic particles between ${\sim }10\,R_{s}$ and Earth orbit, simultaneously, with imaging and spectroscopic observations of the SEP source regions on the Sun from multiple vantage points. In tandem, these two historic missions with their unprecedented inner-heliospheric perspectives will be combined with data from other missions to address key questions regarding large SEP events. For instance, SEP-related goals of the SPP mission will address the following:

- Origin: What are the seed populations and physical conditions necessary for energetic particle acceleration?
- Acceleration: What are the roles of evolving shocks, reconnection, waves, and turbulence in the acceleration of energetic particles?
- Transport: How are energetic particles released from their sources and transported radially and across magnetic field lines from the corona to the heliosphere?

Table 3 summarizes the five key questions discussed above, lists possible physical processes that could play important roles in causing these effects, and how SolO and SPP observations will advance our current knowledge and understanding of SEPs.

## Concluding remarks

Studying SEP origins and acceleration continues to spark the interests of the Solar and Heliospheric community. While significant progress has been achieved recently, a complete physical understanding prevents us from developing reliable and accurate predictive models. In the next decade, SPP and SolO promise to fill many of these gaps, but they will also open up new questions and yield new discoveries that challenge existing paradigms; only through simultaneous observations at strategically dispersed locations can we achieve closure with theoretical and model predictions. Moreover, as long as we are economically limited to a few in-situ observations of SEP events, we will always lack the big picture that remote imaging can provide for other studies. For example, CME studies are substantially bolstered by tracking type II radio bursts that provide evidence of coronal shocks, establishing the magnetic connection between a particular s/c and the solar source region or surface, and then using global models and imagery to understand the coronal conditions and magnetic field topology at source locations. Consequently, during the 2019–2025 timeframe, when both SPP and SolO are providing the much needed inner heliospheric observations, simultaneous near-Earth in-situ and remote sensing observations will provide the ground-truth observations that are critical for closure with models and for ensuring maximum science return from these missions.

## Footnotes

1 Note that, in this review, the term “transport” includes both acceleration and propagation.

2 In this analysis, the complex nature of magnetic fields extending from the solar photosphere, where supergranulation is seen to occur and the source surface where the SW is assumed to be fully accelerated to the speed, $U_w$, is ignored. It is assumed that transverse random plasma motions at the photosphere also occur at the source surface.

3 As was noted in the Giacalone and Jokipii (2004) paper, the value of $\kappa _{{ sg}}\sim 1900\,\mathrm{km^{2}/s}$ is larger than that found previously by Smithson (1973), Schrijver and Martin (1990), and Wang et al. (2002), and more recently Hathaway et al. (2010). The cause of this discrepancy is still not clear, but may be related to the assumption of a divergence-free plasma velocity associated with solar supergranulation, as assumed by Giacalone and Jokipii (2004).

4 Increased magnetic-field turbulence in the shocked plasma behind the shock, which is commonly observed, will likely reduce the diffusion coefficient. This can easily be included in the analysis, but is not necessary since the steady-state solution for *f* does not depend on the downstream diffusion coefficient.

5 Bohm diffusion, in the context of our discussion, is the limit in which the particle scattering frequency is equal to the cyclotron frequency. This is also the limit in which the parallel mean free path is equal to the particle gyro-radius.
